# Vaccination with SARS-CoV-2 spike protein lacking glycan shields elicits enhanced protective responses in animal models

**DOI:** 10.1126/scitranslmed.abm0899

**Published:** 2022-04-06

**Authors:** Han-Yi Huang, Hsin-Yu Liao, Xiaorui Chen, Szu-Wen Wang, Cheng-Wei Cheng, Md. Shahed-Al-Mahmud, Yo-Min Liu, Arpita Mohapatra, Ting-Hua Chen, Jennifer M. Lo, Yi-Min Wu, Hsiu-Hua Ma, Yi-Hsuan Chang, Ho-Yang Tsai, Yu-Chi Chou, Yi-Ping Hsueh, Ching-Yen Tsai, Pau-Yi Huang, Sui-Yuan Chang, Tai-Ling Chao, Han-Chieh Kao, Ya-Min Tsai, Yen-Hui Chen, Chung-Yi Wu, Jia-Tsrong Jan, Ting-Jen Rachel Cheng, Kuo-I Lin, Che Ma, Chi-Huey Wong

**Affiliations:** 1 Genomics Research Center, Academia Sinica, Taipei 11529, Taiwan.; 2 GIP-TRIAD Master’s Program in Agro-Biomedical Science, National Taiwan University College of Medicine, Taipei 100233, Taiwan.; 3 Institute of Biochemical Sciences, National Taiwan University, Taipei 10617, Taiwan.; 4 Institute of Biological Chemistry, Academia Sinica, Taipei 11529, Taiwan.; 5 Biomedical Translation Research Center, Academia Sinica, Taipei 11529, Taiwan.; 6 Institute of Molecular Biology, Academia Sinica, Taipei 11529, Taiwan.; 7 Department of Clinical Laboratory Sciences and Medical Biotechnology, National Taiwan University College of Medicine, Taipei 100233, Taiwan.; 8 Department of Laboratory Medicine, National Taiwan University Hospital and National Taiwan University College of Medicine, Taipei 100233, Taiwan.; 9 Institute of Biomedical Sciences, Academia Sinica, Taipei 11529, Taiwan.; 10 Department of Chemistry, Scripps Research, La Jolla, CA 92037, USA.

## Abstract

A major challenge to end the pandemic caused by severe acute respiratory syndrome coronavirus 2 (SARS-CoV-2) is to develop a broadly protective vaccine that elicits long-term immunity. As the key immunogen, the viral surface spike (S) protein is frequently mutated, and conserved epitopes are shielded by glycans. Here, we revealed that S protein glycosylation has site-differential effects on viral infectivity. We found that S protein generated by lung epithelial cells has glycoforms associated with increased infectivity. Compared to the fully glycosylated S protein, immunization of S protein with N-glycans trimmed to the mono-GlcNAc–decorated state (S_MG_) elicited stronger immune responses and better protection for human angiotensin-converting enzyme 2 (hACE2) transgenic mice against variants of concern (VOCs). In addition, a broadly neutralizing monoclonal antibody was identified from S_MG_-immunized mice that could neutralize wild-type SARS-CoV-2 and VOCs with subpicomolar potency. Together, these results demonstrate that removal of glycan shields to better expose the conserved sequences has the potential to be an effective and simple approach for developing a broadly protective SARS-CoV-2 vaccine.

## A sugar-free vaccine

Vaccines that elicit broadly neutralizing antibodies against severe acute respiratory syndrome coronavirus 2 (SARS-CoV-2) will be essential for curbing the ongoing coronavirus disease 2019 (COVID-19) pandemic. Here, Huang *et al*. evaluated the immunological impact of enzymatically removing glycan shields from the SARS-CoV-2 spike (S) protein. The authors found that immunizing hamsters or mice with S protein lacking glycan shields, termed S_MG_, elicited robust and broadly reactive immune responses. Vaccinated mice were protected from challenge with the alpha, gamma, and delta variants of concern. Antibodies isolated from vaccinated animals could also neutralize the omicron variant. Together, these data support further investigation into S_MG_ as a candidate SARS-CoV-2 vaccine.

## Teaser

A vaccine developed by removing glycan shields in the SARS-CoV-2 S protein conferred protection against infection with variants of concern.

## INTRODUCTION

Spike (S) protein, the main focus of vaccine development for severe acute respiratory syndrome coronavirus 2 (SARS-CoV-2), contains 22 N-linked and at least 2 O-linked glycosylation sites per monomer (*1*–*3*). These sites are important for S protein folding and processing as well as for evading immune recognition by shielding specific epitopes, thus hindering the efficacy of the vaccine (*2*, *3*). Understanding the glycosylation of S protein can uncover the role in which glycans play and guide rational vaccine design (*2*, *3*). The glycan profiles of S protein expressed from various cell sources have been reported, revealing a conserved pattern of 8 specific N-glycosylation sites harboring at least 30% underprocessed high mannose–type and hybrid-type N-glycans, with the remaining 14 sites predominantly of the complex type (*2*, *4*). It was shown that a trimeric S protein with its original complex type–dominant glycoform is more efficient in receptor recognition and viral entry than the high-mannose variants derived either from *N*-acetylglucosaminyltransferase I (GnTI)¯ human embryonic kidney (HEK) 293 (*5*) or alpha-1,3-mannosyl-glycoprotein 2-beta-*N*-acetylglucosaminyltransferase (MGAT1)¯ HEK293T cells (*6*).

In this study, we investigated the differential influences of overall and site-specific glycosylation of the S protein on SARS-CoV-2 infectivity. We further analyzed the glycosylation profile of S protein expressed from lung epithelial cells, the primary cell type infected by SARS-CoV-2, to evaluate the correlation between S protein glycosylation, protein conservation, and glycan shielding. The results led us to generate a mono-GlcNAc–decorated S protein (S_MG_) as a candidate vaccine immunogen to expose the conserved glycan-shielded epitopes on the protein surface. In vitro and in vivo studies of the S_MG_ immunogen as a vaccine against SARS-CoV-2 and variants of concern (VOCs), as well as the isolation of a cross-neutralizing antibody m31A7, suggested that the vaccine design with removal of glycan shields is a simple strategy to develop a broadly reactive coronavirus disease 2019 (COVID-19) vaccine.

## RESULTS

### Glycosylation affects pseudovirus S protein interacting with ACE2 on several cell types

**Fig. 1.**
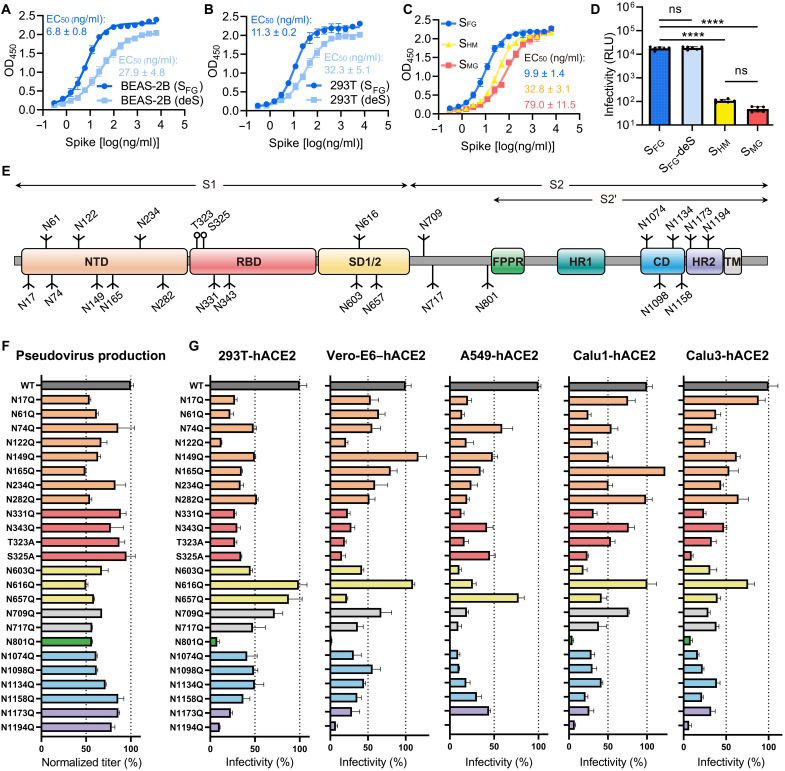
S protein glycosylation impacts ACE2 receptor binding and SARS-CoV-2 infection. (**A** to **C**) Binding avidity of ACE2 was measured for differently glycosylated S protein ectodomains (S_FG_; original fully glycosylated, blue; deS, nonsialylated, light blue; S_HM,_ high-mannose, yellow; and S_MG_, mono-GlcNAc, red) from BEAS-2B (A), HEK293T (B), and HEK293S (GnTI¯) cells without or with Endo H digestion (C). Data of three technical replicates are shown as means ± SD, and curves fit by nonlinear regression for EC_50_ values. (**D**) Viral infectivity was measured for pseudoviruses carrying differently glycosylated S protein with the same input amount (0.3 μg/ml of p24 equivalent) colored accordingly as in (C). RLU, relative luminescence unit. Data of six technical replicates shown as means ± SD and analyzed with ordinary one-way ANOVA test followed by Tukey’s multiple comparisons test. ns, not significant; ****P < 0.0001. (**E**) A schematic view of SARS-CoV-2 S protein [wild type (WT)] is shown colored by domain, including N-terminal domain (NTD; 14–306; orange), receptor binding domain (RBD; 319–541; red), two subdomains (SD1/2; 542–685; yellow), fusion peptide proximal region (FPPR; 816–856; green), heptad repeat 1 (HR1; 912–984; teal), connecting domain (CD; 1063–1162; blue), heptad repeat 2 (HR2; 1163–1211; purple), and transmembrane domain (TM; 1214–1234; white). N-glycan (drawn as branches) and *O-*glycan (circles) sites are marked with residue number. S1 and S2 domains are shown above. (**F**) Viral titers are shown for pseudoviruses carrying WT S protein or mutants with glycans removed at each shown glycosite, normalized by p24 quantification, and colored accordingly as in (E). (**G**) Infectivity of the same panel of pseudoviruses as in (F) tested in five hACE2-expressing cell lines. Values in (F) and (G) are normalized against WT values (defined as 100%, colored in dark gray) with means ± SD of three independent experiments.

To understand the importance of glycosylation, we expressed S protein from lung epithelial cells, the primary cells for infection, and found that sialylation of S protein is required for higher avidity to the receptor (Fig. 1A). A similar pattern was also observed for HEK293T cell–generated S protein (Fig. 1B), and the avidity was also reduced for the S protein with only high-mannose glycans or in the glycoform with all N-glycans trimmed to a single N-acetylglucosamine (GlcNAc) (Fig. 1C). The impact of its glycosylation was further tested by pseudovirus infection in human angiotensin-converting enzyme 2 (hACE2)–expressing HEK293T cells, revealing a consistent trend, when the same amount of virus was applied (Fig. 1D and fig. S1). This allowed us to conclude that complex-type glycans and sialylation are functionally important for S protein–mediated infectivity. A full panel of 24 lentivirus-based pseudovirus variants (comprising the 22 N- and 2 *O*-glycosites) were also generated for evaluating the viral entry efficiency in five hACE2-expressing cell lines, including HEK293T, Vero-E6, and three human lung cell lines, A549, Calu-1, and Calu-3 cells (Fig. 1, E to G). These pseudoviruses were based on the S construct with C-terminal 19 amino acid deletion, which produced the highest viral titer (fig. S2). Pseudovirus production was quantified by a p24 immunoassay, and results were normalized against the titer of each mutant strain (Fig. 1F). Every N-glycosite asparagine (Asn) was substituted to glutamine (Gln) to minimize the structural influence because of their chemical similarity, and each *O*-glycosite threonine (Thr) or serine (Srn) was substituted to alanine (Ala). Because the mutagenesis did change the amino acids, the resulting change in infectivity would come from collective factors, including the glycosylation-related conformational shifts that affect receptor engagement and the surface abundance of S protein affected by protein expression, folding, and trafficking. Results showed that disruption of S protein glycosylation reduced infectivity, which is similar to the findings using HEK293T cells (*7*). A substantial reduction was observed for two mutations in the receptor binding domain (RBD), N331Q and N343Q, as well as for mutations of the two *O*-glycosites (T323A and S325A), despite the low occupancy of the latter (Fig. 1G) (*2*). In addition, deletion of N122 glycosylation in the N-terminal domain (NTD) resulted in reduced infectivity and low protein expression (Fig. 1G and fig. S3A). Two NTD mutations, N149Q and N165Q, increased infectivity in Vero-E6 and Calu-1 cells, respectively, although decreased infections were observed in other cells (Fig. 1G). Note that the glycans attached to this N165 residue are structurally proximal to the neighboring RBD in the trimeric S protein (*2*), and its mutation reduced ACE2 binding probably because of the conformational shift of RBD toward the “down” state (*8*). We identified two mutants, N801Q and N1194Q (Fig. 1F), that universally abolished virus infectivity in all five cells. The glycosite N801 is located near the fusion peptide proximal region (FPPR), and N1194 is near the center of heptad repeat 2 (HR2) helix and is the last N-glycosite preceding the transmembrane domain (Fig. 1E and fig. S3B). These mutations both caused low-yield expression (fig. S3A). The N801Q mutant was more prone to degradation, and the N1194Q mutant disrupted S protein trimerization (fig. S3, C and D), which could be part of the explanation for the reduction of infectivity by pseudoviruses carrying these mutants.

### S protein from lung epithelial cells contains more sialylated complex-type glycans

**Fig. 2.**
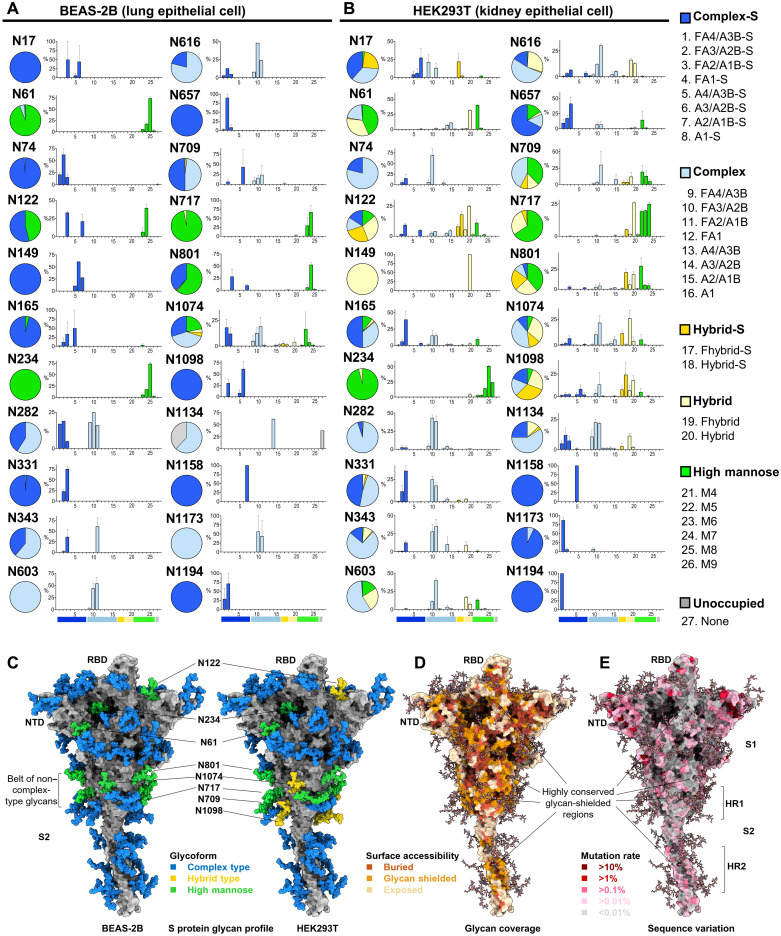
S protein glycan profiles demonstrate differences in two cell lines and correlate with sequence conservation. (**A** and **B**) A comparison of the N-glycosylation profile of recombinant S protein expressed from BEAS-2B lung epithelial cells (A) and HEK293T kidney epithelial cells (B) is shown. Glycans are grouped and colored accordingly: complex-S (sialylated complex type; dark blue), complex (non–sialylated complex type; light blue), hybrid-S (sialylated hybrid type; dark yellow), and hybrid (nonsialylated hybrid type; light yellow), high mannose (green), and unoccupied (gray). The percentage of each group is shown for each glycosite in a pie chart, and the proportion of each glycoform (nos. 1 to 27) in a bar chart. The bar graphs represent the means ± SD of three biological replicates. Detailed structure and percentage of each glycoform can be found in tables S1 to S3 and figs. S4 to S6. Fhybrid indicates fucosylated hybrid-type glycans. (**C**) Glycan profiles from (A) and (B) were mapped on the 3D structure of S ectodomain (modeled from 6VSB). Glycans are colored by the highest-abundance group for BEAS-2B (left) or HEK293T (right) data as labeled (complex type, blue; hybrid type, yellow; and high mannose, green). Non–complex-type N-glycosites are labeled with residue number. (**D**) Mapping of relative surface accessibility (RSA) on modeled S structure protein is shown, with buried residues colored in dark yellow, glycan shielded in yellow, and exposed in light yellow. (**E**) Mapping of sequence variation on modeled S protein structure is shown, colored in a heatmap, with darker red indicating higher mutation rates. Several highly conserved glycan-shielded regions are highlighted. More details for (D) and (E) can be found in fig. S9 and data file S1.

The glycan profile analysis of S protein revealed a higher abundance of complex-type glycans (78%), and fewer hybrid-type glycans (less than 1%) for S protein were produced in the human lung epithelial cell line BEAS-2B (Fig. 2A and table S1) as compared with S protein produced in the human kidney epithelial cell line, HEK293T (61 and 23%, respectively) (Fig. 2B and table S2). Among the high mannose–type glycans, the N-linked mannose-5 glycan (man5) was the predominant type found across the HEK293T-expressed S protein, although it is only seen at site N61 from BEAS-2B cells (figs. S4 and S5). In addition, the complex-type glycans at sites N74, N149, N282, and N1194 from BEAS-2B cells were more diversely processed (multiple antennae, galactosylation, fucosylation, or sialylation) than those from HEK293T cells. In contrast, the glycans at sites N122, N331, N1098, and N1134 were less diverse (figs. S4 and S5). Furthermore, N149 and N17 harbored no core fucose in BEAS-2B. We observed an overall higher degree of sialylation on all 22 N-glycosites from BEAS-2B (53%) than that from HEK293T (35%), HEK293E (26%) (tables S1 to S3 and fig. S6), or the previously reported HEK293F cells (15%) (*2*). Particularly, the two N-glycosites (N331 and N343) of RBD are more sialylated in BEAS-2B (99 and 39%) than in HEK293T (49 and 15%) (tables S1 and S2). Despite the differences, the S protein from all cell types contains a non–complex-type glycan belt located around the middle section of the S2 domain (Fig. 2C and fig. S7), where the N-glycosite N801 is critical for infection (Fig. 1G), N1074 contains diverse glycans (figs. S4 to S6), and N717 is essential for S protein expression (fig. S3A).

### Highly conserved epitopes in S protein are largely shielded by glycans

From the modeled SARS-CoV-2 S protein structure and the glycan profile from BEAS-2B cells, we conducted structural analysis of glycan coverage over protein surface areas and overlaid with multiple alignment results using 1,117,474 S protein sequences (*9*). It revealed several regions that were highly conserved, yet shielded by glycans, including the lower flank of RBD, the S2 stem region with the non–complex-type glycan belt, and the C-terminal part of S2 involving the connecting domain (CD) and HR2 (Fig. 2, D and E). On the primary sequence level, these regions were shown as conserved epitopes (fig. S8). Sequence conservation analysis also showed that most of the glycosite regions were highly conserved (fig. S9A), and the most conserved ones (mutation rate lower than 0.02%) included those in the NTD (N61, N122, N165, and N234), the RBD (S325, N331, and N343), the subdomain 1/2 (SD1/2) (N603 and N657), the stem region of subunit 2 (S2) (N709, N1098, N1134, N1158, N1173, and N1194), and N801 near the FPPR. Most of these regions contain around 20 to 40% of conserved surface residues (fig. S9B), and among them, a certain percentage of residues were shielded by glycans, 36% in the RBD and about 50% in other regions (fig. S9C). Although harboring no N-glycosites, the HR1 region had 69% of its conserved surface residues being covered by glycans stemmed from adjacent domains (fig. S9C). These results highlighted the importance of S protein glycosylation, both structurally and evolutionarily, leading to the thought that exposing glycan-shielded conserved regions may elicit immune responses against conserved epitopes.

### S_MG_ was developed as a vaccine

**Fig. 3.**
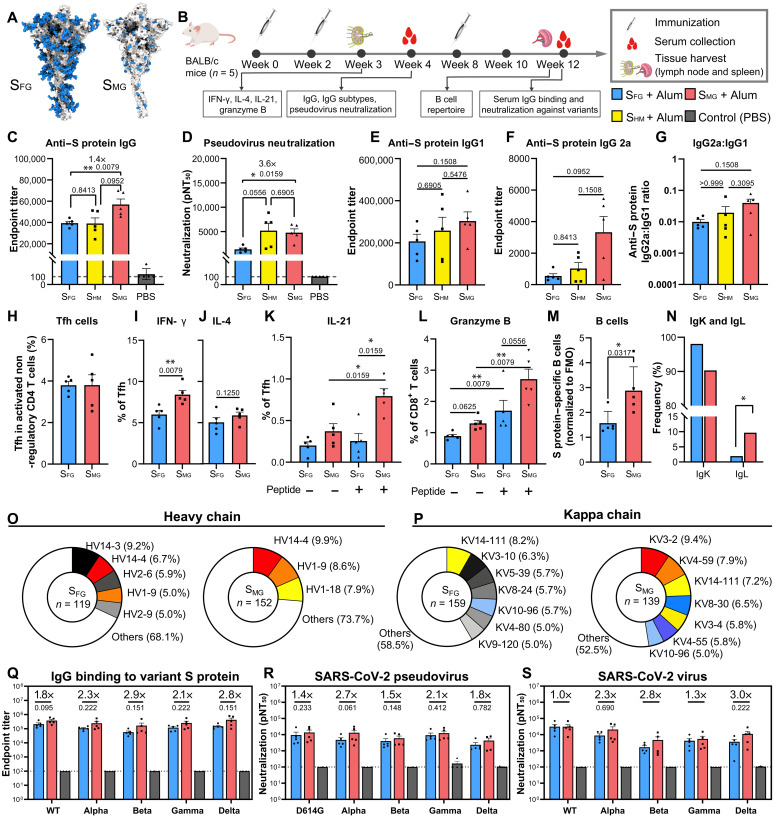
S_MG_ vaccination elicits stronger humoral and cellular immune responses than S_FG_ in BALB/c mice. (**A**) Structural models of S_FG_ and S_MG_ protein vaccine are shown (according to Fig. 2C). Blue: glycans; gray: protein. S_FG_ was expressed by HEK293E without further modification. S_MG_ was obtained by enzymatic digestion to truncate all N-glycans of S_HM_ expressed by HEK293S GnTI¯ to single GlcNAc, whereas *O*-glycans were unmodified. (**B**) Immunization schedule using proteins as in (A) as immunogens in BALB/c mice (*n* = 5 in each experiment). S_FG_ (blue), S_HM (_yellow), S_MG_ (red), and control (gray). Alum, aluminum hydroxide. (**C**) Anti–S protein IgG titers of serum samples were analyzed by ELISA. (**D**) Neutralization titers of serum samples were measured using pseudovirus with WT S protein. (**E** to **G**) IgG subtype analysis of sera, including IgG1 (E), IgG2a (F), and the IgG2a:IgG1 ratio (G). (**H** to **K**) The percentage of Tfh in activated nonregulatory CD4 T cells (H) and the percentages of IFN-γ (I)–, IL-4 (J)–, and IL-21 (K)–expressing Tfh cells (CD4^+^CD19^−^CD44^hi^Foxp3^−^PD-1^+^CXCR5^+^) in the lymph nodes (LNs) of BALB/c mice by flow cytometry. (**L**) The percentage of granzyme B−producing CD8^+^ T cells (CD3^+^ B220^−^CD8^+^ CD49b^−^) in the LN of BALB/c mice analyzed by flow cytometry. (**M**) The ratio of S protein–specific B cells (CD3^−^CD19^+^S protein^+^) (percentage) normalized to fluorescence minus one (FMO) control staining (stained without S protein) (percentage) in the spleen is shown. (**N**) Kappa and lambda light chain usage is shown. (**O** and **P**) Heavy (O) and kappa (P) chain distribution of B cell repertoire analysis. Less than 5% usage is shown in white. (**Q** to **S**) Anti−S protein IgG titers (Q), pseudovirus neutralization titers (R), and authentic virus neutralization titers (S) are shown for serum isolated from BALB/c mice after three doses of indicated vaccines against SARS-CoV-2 WT (or D614G) and variants (number above each bar indicate fold of increase of S_MG_ compared to S_FG_ group). pNT_50_ represents the reciprocal dilution achieving 50% neutralization. The dotted line in bar charts represents the lower limit of detection. Data are shown as means ± SEM and analyzed by two-sided Mann-Whitney *U* test to compare two experimental groups, except in (N), where five samples were pooled together and a chi-squared test was used. *P* values shown above each bar. *P < 0.05; **P < 0.01.

Our initial attempt to mutate multiple glycosites led to a markedly reduced expression of S protein (fig. S3A). Yet, when we expressed it from GnTI¯ HEK293S cells (fig. S10, A and B), we were able to produce a high-mannose glycoform S protein (S_HM_) with good yield and purity (fig. S10C). We then trimmed the glycans using endoglycosidase H (Endo H) to a single GlcNAc at each N-glycosite (fig. S10, A and B) (*10*, *11*), generating a soluble trimmer mono-GlcNAc–decorated S protein, which we called S_MG_ (Fig. 3A and fig. S10, D and E). S_MG_ was confirmed by mass spectrometry that all N-glycosites mostly occupied with single GlcNAc (fig. S10F), and the occupancies of untreated *O*-glycans were too low to be detected. This modified S_MG_, and S_HM_, as well as the original fully glycosylated S protein (S_FG_) were mixed with aluminum hydroxide (alum) as an adjuvant and were then used to immunize BALB/c mice (*n* = 5) by intramuscular injection (Fig. 3B). The S_FG_ used for comparison in this study was expressed by HEK293E cells and contained diverse glycans; this is similar to the immunogens used in many current COVID-19 vaccines that are either approved or in clinical trials, including the insect cell–expressed S protein vaccines from Sanofi and Novavax (*1*), the Chinese hamster ovary (CHO) cell–expressed recombinant S vaccine from Medigen (*12*), the adenovirus-based vaccines from AstraZeneca and Johnson & Johnson, and the mRNA vaccines from Pfizer-BioNTech and Moderna (*1*). Both S_FG_ and S_MG_ proteins demonstrate essentially the same trimeric structure in solution by negative staining analysis (fig. S10, G and H).

### S_MG_ vaccine elicited better immune response using different antibody subclasses

Mice immunized with S_MG_ induced superior humoral immune response after second immunization as compared with S_FG_, with a 1.44-fold significantly higher immunoglobulin G (IgG) titer against S protein (end point titer: S_FG_, 39,408 ± 1,619; S_MG_, 56,957 ± 5,091; *P* = 0.0079) (Fig. 3C) and 3.6-fold stronger antibody neutralization potency based on the inhibition of SARS-CoV-2 pseudovirus infection (reciprocal half maximal neutralization titer pNT_50_: S_FG_, 1346 ± 285; S_MG_, 4791 ± 767; *P* = 0.0159) (Fig. 3D), whereas S_HM_-immunized group shows similar anti–S IgG titers (39,086 ± 11,654) and no difference in pNT_50_ titer compared with the S_FG_ group. The analysis of IgG subtype titer and interferon-γ (IFN-γ) or interleukin-4 (IL-4) production by T follicular helper (Tfh) cells revealed that S_MG_ vaccine induced more IgG2a, which is the marker for T helper 1 cell (T_H_1) lymphocytes in BALB/c mice, a more balanced T_H_1/T_H_2 response, and more IFN-γ–expressing Tfh cells compared with the S_FG_- and S_HM_-vaccinated groups (Fig. 3, E to J). Furthermore, the S_MG_ vaccine induced higher frequency of IL-21^+^ Tfh cells (Fig. 3K) and an elevated frequency of granzyme B–producing CD8^+^ T cells (Fig. 3L). These data indicated that a more potent humoral and cellular adaptive immune response was elicited by S_MG_, as compared with that induced by S_FG_. We then examined the frequency of S protein–specific B cells (CD3^−^CD19^+^S^+^) from the spleen of mice immunized after the third dose of S_FG_ or S_MG_ (Fig. 3A) and found that mice immunized with S_MG_ generated more S protein–specific B cells (Fig. 3M and fig. S11, A and B). The B cell repertoire analysis from S_FG_- and S_MG_-immunized mice (*n* = 5) indicated that more lambda light chain genes were used in the S_MG_ group compared with that in the S_FG_ group (S_FG_, 1.92%; S_MG_, 9.68%) (Fig. 3N). In addition, antibodies derived from several specific loci of the Ig heavy chain variable region (*IGHV*) (Fig. 3O and fig. S11C) and the Ig kappa chain variable region (*IGKV*) genes (Fig. 3P and fig. S11D) were overrepresented in the S_MG_ group than in the S_FG_ group, especially the *IGHV1-18* gene (Fig. 3O). This finding suggested that B cell epitopes may be processed differently in these two groups, and it remains to be further explored whether and why this difference is immunologically beneficial. In addition, the three-dose S_MG_ vaccination elicited higher end point titer IgG than the two-dose vaccination against wild-type (WT) S protein (end point titer: S_FG_, 208,911 ± 50,092; S_MG_, 376,410 ± 80,873). We observed differences between the S_MG_ and S_FG_ groups in serum IgG-binding curves measured by enzyme-linked immunosorbent assay (ELISA) against the S protein from SARS-CoV-2 VOCs (*13*), including alpha (B.1.1.7; *P* = 0.0488), beta (B.1.351; *P* = 0.0010), gamma (P.1; *P* = 0.0068), and delta (B.1.617.2; *P* = 0.0068) (fig. S12A), but no statistical differences in end point titers analysis (Fig. 3Q). Differences in neutralizing antibody responses against VOCs by pseudovirus neutralization curve, including alpha (*P* = 0.0156), beta (*P* = 0.0156), and delta (*P* = 0.0078) (fig. S12B), were also observed, but no differences were observed in the pNT_50_ titer values for pseudovirus or authentic virus neutralization (Fig. 3, R and S; and fig. S12, C and D), as compared with S_FG_. These VOCs are of particular concern because they have shown resistance to several therapeutic antibodies and convalescent serum and because they have exhibited reduced protection by many approved vaccines (*14*–*18*).

### S_MG_ vaccine provided superior protection against SARS-CoV-2 and variants in vivo

**Fig. 4.**
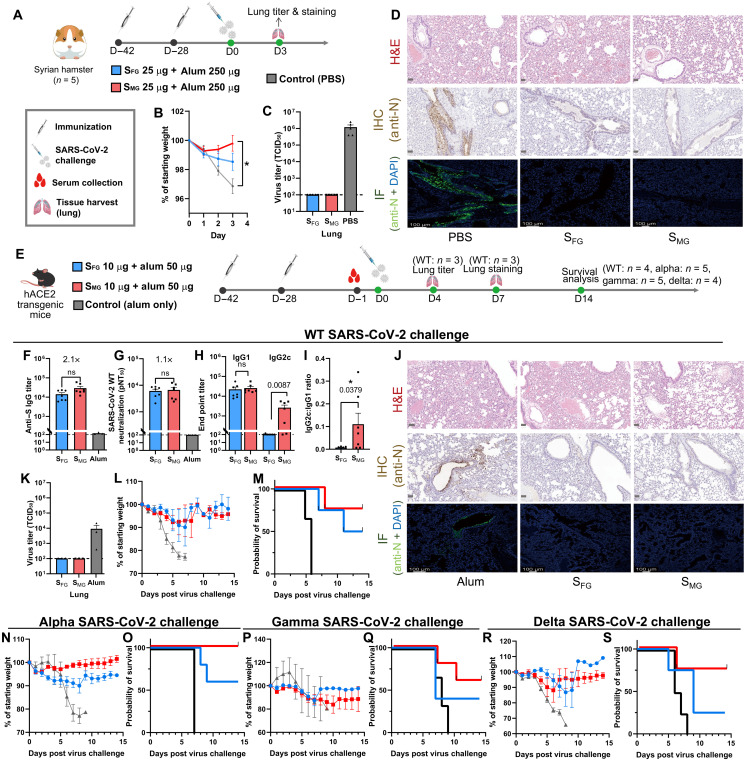
S_MG_ vaccination provides enhanced protection against SARS-CoV-2 infection in vivo. (**A**) The immunization schedule for Syrian hamsters is shown. S_FG_ (blue), S_MG_ (red), and control (gray). (**B**) Weight change was measured in Syrian hamsters after WT SARS-CoV-2 challenge. (**C**) Lung virus titers of challenged hamsters are shown. The dashed line indicates the lower limit of detection. (**D**) Representative images shown histopathology, immunohistochemistry, and immunofluorescence of the lungs from an infected hamster (3 dpi). First row: Hematoxylin and eosin (H&E) staining; scale bar, 50 μm. Second row: Immunohistochemistry (IHC) staining; scale bar, 50 μm. Third row: Immunofluorescence (IF) staining; scale bar, 100 μm. SARS-CoV-2 N-specific polyclonal antibodies were used for virus detection as brown dots in IHC and green dots in IF staining. Blue: 4,6-diamidino-2-phenylindole (DAPI). (**E**) The immunization schedule for CAG-hACE2 or K18-hACE2 transgenic mice is shown. (**F** to **I**) Anti−S IgG titers (F), SARS-CoV-2 WT microneutralization titers (G), and subtype IgG analysis, including IgG1, IgG2c (H), and IgG2c:IgG1 ratio (I), are shown for serum samples collected from immunized CAG-hACE2 transgenic mice (*n* = 7). (**J**) Representative histopathology, immunohistochemistry, and immunofluorescence of the infected mouse lungs (7 dpi) are shown. Scale bars are the same as in (D). (**K**) Lung virus titers of the infected CAG-hACE2 mice (*n* = 3). The dashed line indicates the lower limit of detection. (**L** and **M**) Weight change (L) and survival analysis (M) are shown for WT-SARS-CoV-2–challenged CAG-hACE2 transgenic mice (*n* = 4). (**N** and **O**) Weight change (N) and survival analysis (O) are shown for SARS-CoV-2 alpha variant–challenged CAG-hACE2 transgenic mice (*n* = 5). (**P** and **Q**) Weight change (P) and survival analysis (Q) are shown for SARS-CoV-2 gamma variant–challenged CAG-hACE2 transgenic mice (*n* = 5). (**R** and **S**) Weight change (R) and survival analysis (S) are shown for SARS-CoV-2 delta variant–challenged K18-hACE2 transgenic mice (*n* = 4). Data are shown as means ± SEM and analyzed by two-sided Mann-Whitney *U* tests to compare two experimental groups. ns, not significant; *P < 0.05.

To evaluate the in vivo protective efficacy of S_MG_ vaccine against SARS-CoV-2, we first carried out WT SARS-CoV-2 challenge in Syrian hamsters vaccinated with S_MG_ or S_FG_ (Fig. 4A). S_MG_-vaccinated hamsters (*n* = 5) showed less reduction in body weight as compared with the S_FG_ and phosphate-buffered saline (PBS) groups (Fig. 4B), whereas similar virus titer reductions were observed in the lungs of both S_FG_- and S_MG_-vaccinated hamsters (Fig. 4C). In addition, according to histopathological staining and anti–nucleocapsid (N) protein immunostaining data, fewer lesions were observed in the lungs of immunized hamsters (Fig. 4D and fig. S13). Because hamsters only showed mild to moderate sickness upon SARS-CoV-2 infection, we then used severe disease models, the highly susceptible CAG-hACE2 (*19*) or K18-hACE2 (*20*) transgenic mice (Fig. 4E). The analysis of anti–S IgG binding titer, neutralizing titers, anti–S subtype IgG, and IgG2c:IgG1 ratio (Fig. 4, F to I) in CAG-hACE2 mice all showed similar results to the BALB/c mice (Fig. 3, C to G). Following challenge with WT SARS-CoV-2 intranasally, virus was not detectable in the lungs of both S_FG_- and S_MG_-vaccinated CAG-hACE2 mice (*n* = 3) by anti-N staining at 7 days postinfection (dpi) (Fig. 4J) or median tissue culture infectious dose (TCID_50_) assay at 4 dpi (Fig. 4K), whereas a viral titer of over 1,000 TCID_50_ was observed in the control group (Fig. 4K). The S_MG_ group (*n* = 4) exhibited better (75%) survival rate than the S_FG_ (50%) at 14 dpi (Fig. 4, L and M). We then evaluated the degree of protection conferred by S_MG_ vaccination against challenge with the alpha variant in CAG-hACE2 mice (*n* = 5). We found that S_MG_ vaccination provided 100% survival rate until 14 dpi (Fig. 4, N and O). S_MG_-vaccinated mice also showed a 60% survival rate in the gamma variant challenge in CAG-hACE2 mice (*n* = 5) (Fig. 4, P and Q) and a 75% survival rate in the delta variant challenge in K18-hACE2 mice (*n* = 4) (Fig. 4, R and S), whereas less than 50% S_FG_-vaccinated mice survived until 14 dpi in the gamma and delta variant challenge (Fig. 4, Q to S). The improved in vivo protection conferred by S_MG_ vaccination provides further evidence that removal of glycan shields from an immunogen is an advantageous strategy to elicit superior immune response.

### A broadly neutralizing antibody was isolated from B cells of mice immunized with S_MG_

**Fig. 5.**
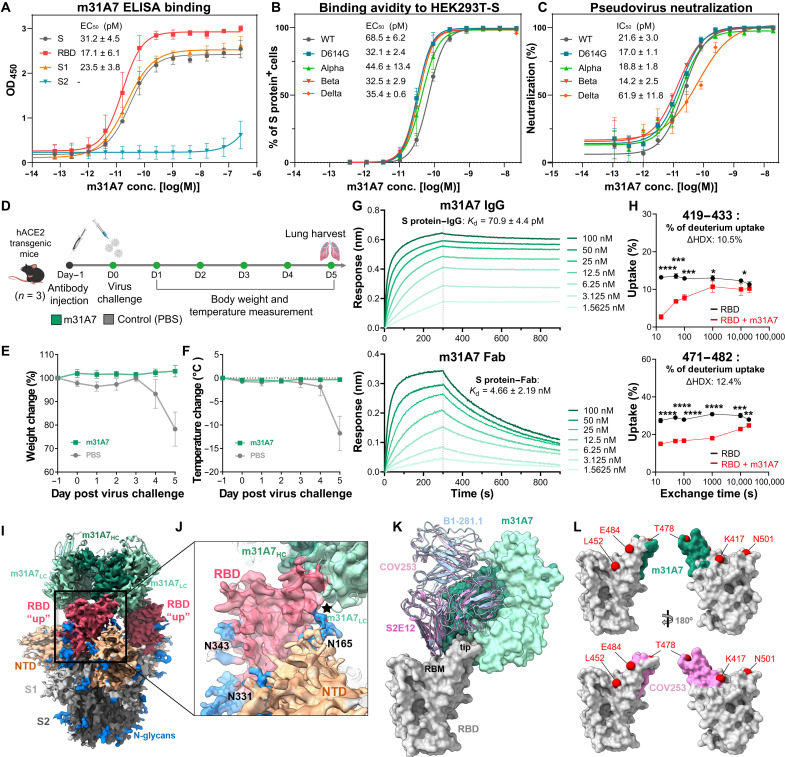
Functional, prophylactic, and structural characterization of antibody m31A7 elicited by S_MG_ vaccination indicates cross-neutralizing capacity. (**A**) ELISA binding of m31A7 to S1, S2, RBD, or the entire S ectodomain. (**B**) Flow cytometry analysis of m31A7 binding to HEK293T cells expressing S protein of SARS-CoV-2 WT and variants. (**C**) Neutralization activity of m31A7 against pseudoviruses carrying WT or variant S proteins. Data of three technical replicates for (A), (B), and (C) are shown as means ± SD and curves fit by nonlinear regression for EC_50_ values. (**D**) Antibody injection and challenge schedule for K18-hACE2 transgenic mice (*n* = 3) is shown. (**E** and **F**) Weight change (E) and body temperature change (F) are shown for mice treated with m31A7 or PBS. Data are presented as means ± SEM. (**G**) Binding kinetics of m31A7 IgG and Fab to S protein are presented, with dissociation constants (*K*_d_) shown above. (**H**) Epitope mapping by HDX-MS of m31A7 is shown in a time course revealing two peptide candidates, 419–433 and 471–482, with greater than 10% ΔHDX at 15 s. Data are shown as means ± SD and analyzed by multiple t tests at each time point. *P < 0.05; **P < 0.01; ***P < 0.001; ****P < 0.0001. (**I**) The cryo-EM map fitted with m31A7-Fab/S protein complex structure is shown. Heavy chain, dark green; light chain, light green; RBD, red; NTD, orange; the rest of S1, light gray; S2, dark gray; and N-glycans, blue. (**J**) An enlarged view of RBD-m31A7 interface is shown. The star marks the vicinity between m31A7 light chain and N165-glycan. (**K**) Superimposition of previously reported mAbs S2E12 (magenta), COV253 (pink), and B1-182.1 (light blue) (PDB 7BEN, 7K4N, and 7MLZ) onto the m31A7-bound RBD (gray). The receptor binding motif and RBD tip are highlighted. (**L**) A footprint comparison of COV253 (pink) and m31A7 (green) on RBD (gray) shows similarity, with residues of VOCs labeled and drawn as red spheres.

The sorting of S protein–specific B cells from S_MG_-immunized mice led to the identification of a monoclonal antibody (mAb) m31A7 from the *IGHV1-18* amplified clones, a subset that is uniquely abundant in S_MG_-immunized B cell repertoire (Fig. 3O and fig. S11B). This mAb interacts with the full-length S protein, S1, and RBD, but not S2 (Fig. 5A), and binds to HEK293T cells that express the S protein from different SARS-CoV-2 variants (Fig. 5B). In addition, m31A7 was shown to neutralize various pseudovirus variants (WT, D614G, alpha, beta, and delta) at subpicomolar half-maximal inhibitory concentration (IC_50_) up to 1000-fold higher than the reported human mAb EY6A (Fig. 5C and fig. S14) (*21*). A prophylactic study also demonstrated good in vivo efficacy of m31A7 in K18-hACE2 mice (*n* = 3) challenged with WT SARS-CoV-2 (Fig. 5D). Prophylactically treated mice maintained both body weight and temperature (Fig. 5, E and F). Biolayer interferometry (BLI) analysis was used to measure the dissociation constants of m31A7 and its Fab binding to S protein at 70.9 pM and 4.66 nM, respectively (Fig. 5G). Epitope mapping by hydrogen-deuterium exchange mass spectrometry (HDX-MS) revealed its potential binding regions on RBD (Fig. 5H and fig. S15), which overlapped with the observed epitope in the crystal structure of RBD in complex with m31A7-Fab (fig. S16). The cryo–electron microscopy (EM) structure further clarified the binding of m31A7 only to RBD in the “up” state (Fig. 5I), with the N165-glycan from neighboring NTD in the vicinity of the RBD-m31A7 interface (Fig. 5J). The footprint of m31A7 on the RBD is similar to that of human *VH1-58* class mAbs (Fig. 5K) (*14*, *22*, *23*), but it approaches the RBD from a different angle, with shifted local contact areas on the tip loop, bypassing most of key mutated residues of VOCs such as E484 and K417, but not T478 (Fig. 5L) (*22*, *23*). The detailed RBD-m31A7 interface and the inhibitory mechanism of this S_MG_-elicited mAb are under further investigation.

## DISCUSSION

Two years into the current global pandemic, a next-generation, broadly protective COVID-19 vaccine remains urgently needed. Although numerous studies have been published since the outbreak evaluating S protein glycosylation as well as different strategies for vaccine development, our work on a glycoengineered vaccine combines these two fields. The clinical translation of the current findings is promising, as protein-based vaccine platforms have been widely accepted with proven efficacy (*24*). Furthermore, the enzymatic digestion process to remove unwanted glycans is straightforward and could be readily incorporated into current manufacturing procedures. In a parallel effort, a glycoengineered mRNA vaccine recently developed by our group also showed impressive results, supporting our design concept that uncovering glycan-shielded epitopes led to a better vaccine with improved cross-protection (*25*).

A major concern at the present time is the inevitable emergence of new SARS-CoV-2 variants that carry immune-escape mutations (*14*, *26*). Not a single N-glycosite mutation has been observed in any SARS-CoV-2 VOC identified so far except for the moderately conserved N17 site; this is true even for the omicron variant, which has more than 30 mutations in the S protein (*27*), again highlighting the importance of S protein glycosylation during viral evolution. In addition, an interesting comparison can be made between our S_MG_ vaccine and other RBD-based vaccines, because S protein RBD, in its isolated form, is also barely covered by glycans (with the major coverage from N343). Nonetheless, the RBD contains several variation hotspots that are under stringent selective pressure, whereas our S_MG_ vaccine targets the entire S protein ectodomain, stimulating the elicitation of both RBD and non–RBD-neutralizing antibodies that are critical for cross protection. Moreover, the improved CD4^+^ and CD8^+^ T cell responses elicited by S_MG_ vaccination represent an additional advantage for this protein-based vaccine, especially given the increasing evidence that T cell immunity plays a central role against SARS-CoV-2 infection (*28*).

A matter that may be raised about the glycan removal design is that antibodies elicited by the S_MG_ vaccine may fail to bind to the original glycosylated immunogen because of marked changes at the protein surface. This may have been the case in previous studies of HIV (*2*, *29*), because neutralizing antibodies that target Env protein gp120 (with 480 residues per protomer and about 25 N-glycosites) are facing a heavily glycosylated surface with few accessible protein epitopes. However, S_MG_ vaccines have been shown to be advantageous for both influenza virus and SARS-CoV-2, probably because influenza hemagglutinin (H1N1 HA; with 560 residues per protomer and about 6 to 8 N-glycosites) (*10*) and S protein (with 1270 residues per protomer and 22 N-glycosites) are both low to moderately glycosylated. In addition, these glycan chains are likely flexible enough to allow antibody binding even to the glycan-shielded, conserved epitopes.

We also recognized that there are some limitations of our present study. First, our experiments analyzing the impact of glycosites of S protein on interacting with ACE2 and the glycan profiling of S protein were done in human cell lines. It may be closer to the real scenario if primary lung epithelial cells, lung organoids, or other primary tissues expressing ACE2 can be used. Second, a comprehensive analysis of the differential T cell repertoire remains to be identified, particularly of the CD8 T cell repertoire elicited by S_FG_ and S_MG_ vaccination. It will reveal further information on whether and how glycans on S protein may affect antigen presentation. Third, although we have portrayed the differential *IGHV*, *IGKV*, and *IGLV* locus usage by S_FG_ and S_MG_ vaccination, the importance of those differentially elicited B cell repertoires contributing to the potent protection by S_MG_ vaccination deserved further study. Other mAbs, particularly those belonging to the *IGHV1-18* family, are worthwhile to be cloned and investigated. Last, the strategy has only been validated in animal models and remains to be confirmed in clinical trials.

In conclusion, SARS-CoV-2 S protein glycosylation has major influence on virus infection, protein integrity, and immune responses. The S protein from lung epithelial cells contained more sialylated complex-type glycans to facilitate receptor binding, and glycosites N801 and N1194 were shown to be essential for S protein folding and viral infection. The analysis of cell-specific glycoform distribution, sequence conservation, glycan shielding, and their mutual correlations led to the design of S_MG_ vaccine, in which essentially all glycan shields are removed. This made the conserved epitopes better exposed to the immune system so that more effective and broadly protective B cell and T cell responses could be elicited against the virus and variants (*30*). As illustrated by the broadly neutralizing m31A7 antibody in this study and more to be identified in the future, the conserved epitopes targeted by such antibodies could be used for next-generation vaccine development. In an effort to develop vaccines against the currently circulating and future SARS-CoV-2 variants (*31*–*34*), the impact of glycosylation on viral infection, protein integrity, immune response, and vaccine design as demonstrated in this study should be considered together with other parameters.

## MATERIALS AND METHODS

### Study design

This work aims to analyze the impact of glycosylation of SARS-CoV-2 S protein and investigate the efficiency of a glycoengineered S_MG_ as a vaccine candidate. ACE2 binding ELISA and glycan-engineered pseudovirus neutralization assays were used to understand the impact of glycosylation on receptor binding and viral infectivity. Liquid chromatography–tandem mass spectrometry (LC-MS/MS) was applied to analyze and compare glycan profiles of S protein expressed from different cell lines. Computational modeling and bioinformatics analysis were used to visualize these findings on the three-dimensional (3D) structure in the context of evolutionary conservation. The S_MG_ protein was designed, expressed, purified, characterized for glycan profiling, and injected into BALB/c mice for immune response analyses, including serum titers against SARS-CoV-2 WT and variants by ELISA and neutralization assay, T cell and B cell responses by flow cytometry, and B cell repertoire analysis by single B cell screening assay. The in vivo protection was studied in both hamsters and hACE2 transgenic mice by weight monitoring, virus detection (TCID_50_ and histopathology and immunostaining analysis), and survival analysis. A broad-spectrum ultrapotent antibody m31A7 was identified from S_MG_-immunized mice, with its efficiency analyzed by ELISA, flow cytometry, and pseudovirus neutralization, and its protection tested in transgenic mice, binding affinity by BLI, epitope mapping by HDX-MS, and structure determination by cryo-EM and x-ray crystallography. Mouse or hamster group sizes were determined on the basis of animal availability and power analyses using data from our pivotal experiments, and animals were randomly distributed between groups. No blinding was used throughout all studies, and no outliers were skewed to any one group. The selection of end points was made before the start of each study and decided based on the primary objective of investigating the immune responses to S_MG_ vaccination referring to previous studies (*12*, *19*, *20*). The number of technical or biological replicates varied between experiments as described in the figure legends. These altogether serve as a foundation for further investigation and development of the S_MG._

### SARS-CoV-2 S protein production and characterization

The production of S protein was modified from previous studies (*10*, *35*, *36*), with details in the Supplementary Materials and Methods. pcDNA3.1 was used for construction and expression in HEK293T cells [American Type Culture Collection (ATCC) CRL-3216] or BEAS-2B cells (*Homo sapiens*, lung) (ATCC CRL-9609). The pTT vector was used for large-scale expression in HEK293 EBNA (ATCC CRL-10852) or HEK293S GnTI¯ (ATCC CRL-3022) suspension cells for vaccine studies. Purified S_HM_ (from HEK293S GnTI¯ cells) was further treated with Endo H [New England Biolabs (NEB)] overnight at 25°C in a ratio of 50:1 (w/w) to produce S_MG_. S protein purity was monitored by SDS–polyacrylamide gel electrophoresis, with its binding avidity to hACE2 evaluated by ELISA, its glycan profile analyzed by LC-MS/MS [glycoform categorization followed a previous report (*2*)], and its structural conformation confirmed by negative staining EM (fig. S10, G and H).

### Pseudovirus production with glycan-engineered S protein and glycosite-specific S mutants

The production of glycan-engineered pseudoviruses in (Fig. 1D) followed previous studies (*2*, *6*). For production of glycosite-specific S mutant pseudoviruses (Fig. 1F), HEK293T cells were transiently transfected with pVax-nCoV-SΔ19 construct carrying mutations at each glycosite and luciferase-expressing HIV-1 genome plasmid (pNL4-3.luc.RE). Details of pseudovirus production and infectivity assay can be found in the Supplementary Materials and Methods.

### Informatics analysis of SARS-CoV-2 S protein

A total of 1,117,474 S protein sequences of all available SARS-CoV-2 strains were extracted from the GISAID (Global Initiative on Sharing Avian Influenza Data) database (version: 18 April 2021) (*9*). The S protein 3D structure modeling was constructed by CHARMM-GUI (*37*) and OpenMM (*38*) based on the Protein Data Bank (PDB) file 6VSB_1_1_1 (*39*), with the most abundant glycoform of BEAS-2B data as representative glycan profile (fig. S4). *O*-glycans (T323 and S325) used Neu5Ac (α2,3)Gal (β1,3)GalNAc (α1) as representative (*8*). The definition of transmembrane region was according to UniProt (P0DTC2), and other parameters in CHARMM-GUI were according to Woo *et al*. (*39*). Scripts, parameters, and preoptimized models generated by CHARMM-GUI were used as the input for OpenMM. The protein secondary structure was determined by majority voting in the Dictionary of Secondary Structure of Proteins (DSSP) program (*40*, *41*) and 2Struc web server (*42*). The relative solvent accessibility (RSA) of S protein was calculated by FreeSASA program (*43*) and averaged at each site of residues from three chains. The probe radius in FreeSASA was set to 7.2 Å to approximate the average size of hypervariable loops of antibody complementarity-determining regions (CDRs) (*44*). Residues with RSA above 5% were regarded as exposed, otherwise as buried (*45*). All 3D structure figures were drawn by UCSF ChimeraX (*46*).

### Animal immunization, virus challenge, and prophylactic study of m31A7

For mice vaccination with a two-dose schedule, female 6- to 8-week-old BALB/c mice (*n* = 5) were immunized intramuscularly with 10 μg of purified S_FG_, S_HM,_ or S_MG_ mixed with aluminum hydroxide (50 μg) at days 0 and 14. The serum was collected at day 28 after the first vaccination for evaluation of anti–S IgG abundance, IgG subtype, and neutralizing titers (described in the Supplementary Materials and Methods). The lymph nodes of S_FG_- or S_MG_-immunized mice were collected at day 21 after the first vaccination for T cell response analysis (described in the Supplementary Materials and Methods). For B cell repertoire analysis and serum titers against variants, female 6- to 8-week-old BALB/c mice (*n* = 5) were immunized intramuscularly with 20 μg of purified S_FG_ or S_MG_ mixed with aluminum hydroxide (20 μg) at days 0, 14, and 56; mice were euthanized at day 84 to collect whole blood for anti–S IgG and neutralizing titer evaluation and spleens for sorting of S protein–specific B cells (described in the Supplementary Materials and Methods).

For hamster vaccination and virus challenge study, male 6- to 7-week-old golden Syrian hamsters (*n* = 5) were immunized intramuscularly with 25 μg of purified S_FG_ or S_MG_ mixed with aluminum hydroxide (250 μg) at days 0 and 14. Four weeks after the second immunization, each hamster was intranasally challenged with 1 × 10^4^ TCID_50_ of SARS-CoV-2 (hCoV-19/Taiwan/4/2020) in 100 μl of PBS. Body weight was recorded daily after infection. On day 3 after challenge, hamsters were euthanized by carbon dioxide. The superior lobe of the left lung was fixed in 10% paraformaldehyde for histopathological examination, and the rest of the lung was collected for viral load determination (TCID_50_ assay) as described in the Supplementary Materials and Methods.

For transgenic mouse vaccination and virus challenge study, male 6- to 8-week-old CAG-hACE2 transgenic mice (*19*) or male 12-week-old K18-hACE2 transgenic mice (*20*) (purchased from the Jackson Laboratory) were immunized intramuscularly with 10 μg of purified S_FG_ or S_MG_ mixed with aluminum hydroxide (50 μg) at days 0 and 14. CAG-hACE2 transgenic mice were challenged intranasally 4 weeks after the second immunization with 1 × 10^3^ TCID_50_ of WT SARS-CoV-2. In the first trial (*n* = 3), all mice were euthanized at 7 dpi for histopathological examination of superior lobe of the left lung; in the second trial (*n* = 7), three mice were euthanized at 4 dpi for lung virus titer, and four mice were kept until 14 dpi for survival analysis. Serum was collected 1 day before virus challenge.

For challenge studies using VOCs, CAG-hACE2 mice were challenged with 1 × 10^3^ TCID_50_ of the alpha variant (hCoV-19/Taiwan/792/2020) (*n* = 5) or the gamma variant (hCoV-19/Taiwan/906/2021) of SARS-CoV-2 in 50 μl of PBS per mouse. In addition, K18-hACE2 mice were challenged intranasally 4 weeks after the second immunization with 1 × 10^4^ TCID_50_ of the delta SARS-CoV-2 (hCoV-19/Taiwan/1144/2021) (*n* = 4) in 50 μl of PBS per mice. For all SARS-CoV-2 variant challenge models, body weight for each mouse was recorded daily until 14 dpi.

For prophylactic protection test of antibody, male 8-week-old K18-hACE2 transgenic mice (*n* = 3) were injected intraperitoneally with m31A7 (15 mg/kg) or PBS 1 day before being intranasally challenged with 1 × 10^3^ TCID_50_ of WT SARS-CoV-2 (hCoV-19/Taiwan/4/2020). Body weight and body temperature were recorded daily until 5 dpi. All animal experiments were evaluated and approved by the Institutional Animal Care and Use Committee of Academia Sinica (approval nos. 21-10-1716, 18-12-1272, and 20-10-1522).

### m31A7 isolation by single B cell screening assay and characterization

Primers were designed on the basis of a previous publication (*47*). Polymerase chain reaction (PCR) was performed at 50°C for 30 min, 95°C for 15 min, followed by 40 cycles of incubation at 94°C for 30 s, 50°C for 30 s, and 72°C for 1 min, with a final extension at 72°C for 10 min. Seminested second-round PCR was performed using KOD One PCR master mix (TOYOBO) with 1 μl of unpurified first-round PCR product at 98°C for 2 min, followed by 45 cycles of incubation at 98°C for 10 s, 55°C for 10 s, and 68°C for 10 s, with a final extension at 68°C for 1 min. PCR products were then analyzed by electrophoresis and sequencing. The Ig V and L genes were identified on the international ImMunoGeneTics information system (http://imgt.org/IMGT_vquest/input). Genes were then amplified from second-round PCR product with single gene-specific V and L gene primers containing restriction sites for cloning into the vectors containing human IgH or IgL expression backbone. The chimeric IgH and IgL expression constructs were cotransfected into Expi293 for antibody production. After m31A7 was isolated, the antibody was subsequently evaluated for S protein binding by ELISA and fluorescence-activated cell sorting, pseudovirus neutralization potency, binding kinetics, epitope mapping, and structure determination. Details are in the Supplementary Materials and Methods.

### Statistical analysis

Raw data can be found in data file S1. All data were expressed as the means ± SEM or SD as mentioned individually. In Fig. 1D, ordinary one-way analysis of variance (ANOVA) test followed by Tukey’s multiple comparisons was used to compare data. In Fig. 5H, multiple *t* tests with the two-stage step-up method of Benjamini, Krieger, and Yekutieli multiple comparisons were used to compare each time point. For B cell repertoire analysis, a chi-square test was used. For all serum antibody titer analyses, *P* values were obtained from two-sided Mann-Whitney *U* tests to compare two experimental groups. Curves were fit by nonlinear regression using GraphPad Prism 9.0, and comparisons were performed by Wilcoxon matched pairs signed rank test (two-tailed). *P* < 0.05 was considered statistically significant. *P < 0.05; **P < 0.01; ***P < 0.001; ****P < 0.0001.

## Supplementary Material

20220103-1

## Supplementary Materials

### This PDF file includes:

Materials and Methods Figs. S1 to S16 Tables S1 to S3 References (*48*–*52*)

### Other Supplementary Material for this manuscript includes the following:

Data file S1

MDAR reproducibility checklist

## Notes

View/request a protocol for this paper from *Bio-protocol*.
