# Supplementary material for: Vaccination with SARS-CoV-2 spike protein lacking glycan shields elicits enhanced protective responses in animal models
Source: Sci Transl Med. 2022 Apr 6;14(639):eabm0899. doi: 10.1126/scitranslmed.abm0899 (PMC9802656; doi:10.1126/scitranslmed.abm0899)
Supplement: 20220103-1 [file scitranslmed.abm0899.v1.pdf]

Cite as: H.-Y. Huang *et al.*, *Sci. Transl. Med.*
10.1126/scitranslmed.abm0899 (2022).

CORONAVIRUS

Vaccination with SARS-CoV-2 spike protein lacking glycan shields elicits enhanced protective responses in animal models

Han-Yi Huang[1,7]†, Hsin-Yu Liao[1]†, Xiaorui Chen[1]†, Szu-Wen Wang[1,8]†, Cheng-Wei Cheng[1], Md. Shahed-Al-Mahmud[1], Yo-Min Liu[1], Arpita Mohapatra[1], Ting-Hua Chen[1], Jennifer M. Lo[1], Yi-Min Wu[9], Hsiu-Hua Ma[1], Yi-Hsuan Chang[1,8], Ho-Yang Tsai[1,8], Yu-Chi Chou[10], Yi-Ping Hsueh[3], Ching-Yen Tsai[3], Pau-Yi Huang[3], Sui-Yuan Chang[4,5], Tai-Ling Chao[4], Han-Chieh Kao[4], Ya-Min Tsai[4], Yen-Hui Chen[6], Chung-Yi Wu[1], Jia-Tsrong Jan[1], Ting-Jen Rachel Cheng[1], Kuo-I Lin[1*],Che Ma[1*] and Chi-Huey Wong[1,2*]

[1]Genomics Research Center, Academia Sinica, Taipei 11529, Taiwan [2]Department of Chemistry, Scripps Research, La Jolla, CA 92037, USA [3]Institute of Molecular Biology, Academia Sinica, Taipei 11529, Taiwan [4]Department of Clinical Laboratory Sciences and Medical Biotechnology, National Taiwan University College of Medicine, Taipei 100233, Taiwan [5]Department of Laboratory Medicine, National Taiwan University Hospital and National Taiwan University College of Medicine, Taipei 100233, Taiwan

[6]Institute of Biomedical Sciences, Academia Sinica, Taipei 11529, Taiwan [7]GIP-TRIAD Master's Program in Agro-biomedical Science, National Taiwan University College of Medicine, Taipei 100233, Taiwan [8]Institute of Biochemical Sciences, National Taiwan University, Taipei 10617, Taiwan [9]Institute of Biological Chemistry, Academia Sinica, Taipei 11529, Taiwan [10]Biomedical Translation Research Center, Academia Sinica, Taipei 11529, Taiwan

†These authors contributed equally to this work

***Corresponding author: Che Ma (cma@gate.sinica.edu.tw), or Kuo-I Lin (kuoilin@gate.sinica.edu.tw), or Chi-Huey Wong (wong@scripps.edu)**

A major challenge to end the pandemic caused by severe acute respiratory syndrome coronavirus 2 (SARS-CoV-2) is to develop a broadly protective vaccine that elicits long-term immunity. As the key immunogen, the viral surface spike (S) protein is frequently mutated, and conserved epitopes are shielded by glycans. Here, we revealed that S protein glycosylation has site-differential effects on viral infectivity. We found that S protein generated by lung epithelial cells has glycoforms associated with increased infectivity. Compared to the fully glycosylated S protein, immunization of S protein with N-glycans trimmed to the mono-GlcNAc-decorated state (S_{MG}) elicited stronger immune responses and better protection for human angiotensin converting enzyme 2 (hACE2) transgenic mice against variants of concern (VOCs). In addition, a broadly neutralizing monoclonal antibody was identified from S_{MG} immunized mice that could neutralize wild type (WT) SARS-CoV-2 and VOCs with sub-picomolar potency. Together, these results demonstrate that removal of glycan shields to better expose the conserved sequences has the potential to be an effective and simple approach for developing a broadly protective SARS-CoV-2 vaccine.

INTRODUCTION

Spike (S) protein, the main focus of vaccine development for severe acute respiratory syndrome coronavirus 2 (SARS-CoV-2), contains 22 N-linked and at least two *O*-linked glycosylation sites per monomer (*1*–*3*). These sites are important for S protein folding and processing as well as for evading immune recognition by shielding specific epitopes, thus hindering the efficacy of the vaccine (*2*, *3*). Understanding the glycosylation of S protein can uncover the role in which glycans play and guide rational vaccine design (*2*, *3*). The glycan profiles of S protein expressed from various cell sources have been reported, revealing a conserved pattern of 8 specific N-glycosylation sites harboring at least 30% underprocessed high-mannose-type and hybrid-type N-glycans, with the remaining 14 sites predominantly of the complex-type (*2*, *4*). It was shown that a trimeric S protein with its original complex-type-dominant glycoform is more efficient in receptor recognition and viral entry than the high-mannose variants derived either from N-acetyl glucosaminyl transferase I $(GnTI)^-$ HEK293 (*5*) or Alpha-1,3-Mannosyl-Glycoprotein 2-Beta-N-Acetyl glucosaminyl transferase $(MGAT1)^-$ HEK293T cells (*6*).

In this study, we investigated the differential influences of overall and site-specific glycosylation of the S protein on SARS-CoV-2 infectivity. We further analyzed the glycosylation profile of S protein expressed from lung epithelial cells, the primary cell type infected by SARS-CoV-2, to evaluate the correlation between S protein glycosylation, protein conservation, and glycan shielding. The results led us to generate a mono-GlcNAc-decorated S protein (S_{MG}) as a candidate vaccine immunogen to expose the conserved glycan-shielded epitopes on the protein surface. In vitro and in vivo studies of the S_{MG} immunogen as a vaccine against SARS-CoV-2 and variants of concern (VOCs), as well as the isolation of a cross-

neutralizing antibody m31A7, suggested that the vaccine design with removal of glycan shields is a simple strategy to develop a broadly-reactive COVID-19 vaccine.

RESULTS

Glycosylation impacts pseudovirus S protein interacting with ACE2 on several cell types.

To understand the importance of glycosylation, we expressed S protein from lung epithelial cells, the primary cells for infection, and found that sialylation of S protein is required for higher avidity to the receptor (**Fig. 1A**). A similar pattern was also observed for HEK293T-cell-generated S protein (**Fig. 1B**), and the avidity was also reduced for the S protein with only high-mannose glycans or in the glycoform with all N-glycans trimmed to a single GlcNAc (**Fig. 1C**). The impact of its glycosylation was further tested by pseudovirus infection in human angiotensin converting enzyme 2 (hACE2)-expressing HEK293T cells, revealing a consistent trend, when the same amount of virus was applied (**Fig. 1D, fig. S1**). This allowed us to conclude that complex-type glycans and sialylation are functionally important for S protein-mediated infectivity. A full panel of 24 lentivirus-based pseudovirus variants (comprising the 22 N- and 2 *O*-glycosites) were also generated for evaluating the viral entry efficiency in five hACE2-expressing cell lines, including HEK293T, Vero-E6, and three human lung cell lines, A549, Calu-1 and Calu-3 cells (**Fig. 1E to G**). These pseudoviruses were based on the S construct with C-terminal 19 amino-acid deletion, which produced the highest viral titer (**fig. S2**). Pseudovirus production was quantified by a p24 immunoassay and results were normalized against the titer of each mutant strain (**Fig. 1F**). Every N-glycosite asparagine (Asn) was substituted to glutamine (Gln) to minimize the structural influence due to their chemical similarity, and each *O*-glycosite threonine (Thr) or serine (Srn) was substituted to alanine (Ala). Since the mutagenesis did change the amino acids, the resulting change in infectivity would come from collective factors, including the glycosylation-related conformational shifts that affect receptor engagement and the surface abundance of S protein affected by protein expression, folding, and trafficking. Results showed that disruption of S protein glycosylation reduced infectivity, which is similar to the findings using HEK293T cells (*7*). A substantial reduction was observed for two mutations in the receptor binding domain (RBD), N331Q and N343Q, as well as for mutations of the two *O*-glycosites (T323A and S325A), despite the low occupancy of the latter (**Fig. 1G**) (*2*). Additionally, deletion of N122 glycosylation in the N-terminal domain (NTD) resulted in reduced infectivity and low protein expression (**Fig. 1G and fig. S3A**). Two NTD mutations, N149Q and N165Q, increased infectivity in Vero-E6 and Calu-1 cells, respectively, although decreased infections were observed in other cells (**Fig. 1G**). It is interesting to note that the glycans attached to this N165 residue are structurally proximal to the neighboring RBD in the trimeric S protein (*2*), and its mutation reduced ACE2 binding probably due to the conformational shift of RBD toward the "down" state (*8*). Most importantly, we identified two mutants, N801Q and N1194Q (**Fig. 1F**) that universally abolished virus infectivity in all five cells. The glycosite N801 is located near the fusion-peptide proximal region (FPPR), and N1194 is near the center of heptad repeat 2 (HR2) helix and is the last N-glycosite preceding the transmembrane domain (**Fig. 1E, fig. S3B**). These mutations both caused low-yield expression (**fig. S3A)**. The N801Q mutant was more prone to degradation, and the N1194Q mutant disrupted S protein trimerization (**fig. S3C and D**), which could be part of the explanation for the reduction of infectivity by pseudoviruses carrying these mutants.

S protein from lung epithelial cells contains more sialylated complex-type glycans.

The glycan profile analysis of S protein revealed a higher abundance of complex-type glycans (78%) and fewer hybrid-type glycans (less than 1%) for S protein produced in the human lung epithelial cell line BEAS-2B **(Fig. 2A, table S1)** as compared to S protein produced in the human kidney epithelial cell line, HEK293T (61% and 23%, respectively) **(Fig. 2B, table S2)**. Among the high-mannose-type glycans, the N-linked mannose-5 glycan (man5) was the predominant type found across the HEK293T-expressed S protein, although it is only seen at site N61 from BEAS-2B cells **(fig. S4 and S5)**. In addition, the complex-type glycans at sites N74, N149, N282, and N1194 from BEAS-2B cells were more diversely processed (multiple antennae, galactosylation, fucosylation or sialylation) than those from HEK293T cells. In contrast, the glycans at sites N122, N331, N1098 and N1134 were less diverse (**fig. S4 and S5**). Further, N149 and N17 harbored no core fucose in BEAS-2B. We observed an overall higher degree of sialylation on all 22 N-glycosites from BEAS-2B (53%) than that from HEK293T (35%), HEK293E (26%) **(table S1 to S3, fig. S6)** or from the previously reported HEK293F cells (15%) (*2*). Particularly, the two N-glycosites (N331 and N343) of RBD are more sialylated in BEAS-2B (99% and 39%) than in HEK293T (49% and 15%) (**table S1 and S2).** Despite the differences, the S protein from all cell types contains a non-complex-type glycan belt located around the middle section of the S2 domain (**Fig. 2C, fig. S7**), where the N-glycosite N801 is critical for infection (**Fig. 1G**), N1074 contains diverse glycans (**fig. S4 to S6**) and N717 is essential for S protein expression (**fig. S3A**).

Highly conserved epitopes in S protein are largely shielded by glycans.

From the modeled SARS-CoV-2 S protein structure and the glycan profile from BEAS-2B cells, we conducted structural analysis of glycan coverage over protein surface areas and overlaid with multiple alignment results using 1,117,474 S protein sequences (*9*). It revealed several regions that were

highly conserved, yet shielded by glycans, including the lower flank of RBD, the S2 stem region with the non-complex-type glycan belt, and the C-terminal part of S2 involving the connecting domain (CD) and HR2 (**Fig. 2D and E**). On the primary sequence level, these regions were shown as conserved epitopes (**fig. S8**). Sequence conservation analysis also showed that most of the glycosite regions were highly conserved (**fig. S9A**), and the most conserved ones (mutation rate lower than 0.02%) included those in the NTD (N61, N122 and N165 and N234), the RBD (S325, N331 and N343), and the subdomain 1/2 (SD1/2) (N603 and N657) and the stem region of subunit 2 (S2) (N709, N1098, N1134, N1158, N1173 and N1194), as well as N801 near the fusion peptide proximal region (FPPR). Most of these regions contain around 20 to 40% of conserved surface residues (**fig. S9B**) and among them a certain percentage of residues were shielded by glycans, 36% in the RBD and about 50% in other regions (**fig. S9C**). Although harboring no N-glycosites, the heptad repeat 1 (HR1) region had 69% of its conserved surface residues being covered by glycans stemmed from adjacent domains (**fig. S9C**). These results highlighted the importance of S protein glycosylation both structurally and evolutionarily, leading to the thought that exposing glycan-shielded conserved regions may elicit immune responses against conserved epitopes.

Mono-GlcNAc-decorated S protein (S_{MG}) was developed as a vaccine.

Our initial attempt to mutate multiple glycosites led to dramatically reduced expression of S protein (**fig. S3A**). Yet, when we expressed it from GnTI$^-$ HEK293S cells (**fig. S10A and B**), we were able to produce a high-mannose-glycoform S protein (S_{HM}) with good yield and purity (**fig. S10C**). We then trimmed the glycans using Endoglycosidase H (Endo H) to a single N-acetylglucosamine (GlcNAc) at each N-glycosite (**fig. S10A and B**) (*10*, *11*), generating a soluble trimmer mono-GlcNAc-decorated S protein, which we called S_{MG} (**Fig. 3A**, **fig. S10D and E**). S_{MG} was confirmed by mass spectrometry that all N-glycosites mostly occupied with single GlcNAc (**fig. S10F**) and the occupancies of untreated *O*-glycans were too low to be detected. This modified S_{MG}, and S_{HM}, as well as the original fully glycosylated S protein (S_{FG}), were mixed with aluminum hydroxide (alum) as an adjuvant and were then used to immunize BALB/c mice (n=5) by intramuscular injection (**Fig. 3B**). The S_{FG} used for comparison in this study was expressed by HEK293E cells, and contained diverse glycans; this is similar to the immunogens used in many current coronavirus disease 2019 (COVID-19) vaccines that are either approved or in clinical trials, including the insect-cell expressed S protein vaccines from Sanofi and Novavax (*1*), the CHO-cell expressed recombinant S vaccine from Medigen (*12*), the adenovirus-based vaccines from AstraZeneca and J&J, and the mRNA vaccines from Pfizer-BioNTech and Moderna (*1*). Both S_{FG} and S_{MG} proteins demonstrate essentially the same trimeric structure in solution by negative staining analysis (**fig. S10G and H**).

S_{MG} vaccine elicited better immune response utilizing different antibody subclasses.

Mice immunized with S_{MG} induced superior humoral immune response after second immunization as compared to S_{FG}, with a 1.44-fold significantly higher IgG titer against S protein (endpoint titer: S_{FG}, 39408 ± 1619; S_{MG}, 56957 ± 5091, p=0.0079) (**Fig. 3C**) and 3.6-fold stronger antibody neutralization potency based on the inhibition of SARS-CoV-2 pseudovirus infection (reciprocal half maximal neutralization titer pNT_{50}: S_{FG}, 1346 ± 285; S_{MG}, 4791 ± 767, p=0.0159) (**Fig. 3D**), whereas S_{HM} immunized group shows similar anti-S IgG titers (39086 ± 11654) and no difference in pNT_{50} titer compared to the S_{FG} group. The analysis of IgG subtype titer and interferon (IFN)-γ or interleukin (IL)-4 production by T follicular helper (Tfh) cells revealed that S_{MG} vaccine induced more IgG2a, which is the marker for Th1 lymphocytes in BALB/c mice, a more balanced Th1/Th2 response and more IFN-γ expressing Tfh cells comparing to the S_{FG} and S_{HM} vaccinated groups (**Fig. 3E to J**). Furthermore, the S_{MG} vaccine induced higher frequency of IL-21$^+$ Tfh cells (**Fig. 3K**) as well as an elevated frequency of granzyme B-producing CD8$^+$ T cells (**Fig. 3L**). These data indicated that a more potent humoral and cellular adaptive immune response was elicited by S_{MG}, as compared with that induced by S_{FG}. We then examined the frequency of S protein-specific B cells (CD3$^-$CD19$^+$S$^+$) from the spleen of mice immunized after the third dose of S_{FG} or S_{MG} (**Fig. 3A**) and found that mice immunized with S_{MG} generated more S protein-specific B cells (**Fig. 3M, fig. S11A and B**). The B cell repertoire analysis from S_{FG} and S_{MG} immunized mice (n=5) indicated that more lambda light chain genes were used in the S_{MG} group compared to that in the S_{FG} group (S_{FG}, 1.92%; S_{MG}, 9.68%) (**Fig. 3N**). Additionally, antibodies derived from several specific loci of the immunoglobulin heavy chain variable region (*IGHV*) (**Fig. 3O, fig. S11C**) and the immunoglobulin kappa chain variable region (*IGKV*) genes (**Fig. 3P, fig. S11D**) were overrepresented in the S_{MG} group than those in the S_{FG} group, especially, the *IGHV1-18* gene (**Fig. 3O**). This finding suggested that B cell epitopes may be processed differently in these two groups, and it remains to be further explored whether and why this difference is immunologically beneficial. In addition, the 3-dose S_{MG} vaccination elicited higher endpoint titer IgG than the 2-dose vaccination against WT S protein (endpoint titer: S_{FG}, 208911 ± 50092; S_{MG}, 376410 ± 80873). We observed differences between S_{MG} and S_{FG} groups in serum IgG binding curves measured by enzyme-linked immunosorbent assay (ELISA) against the S protein from SARS-CoV-2 VOCs (*13*), including Alpha (B.1.1.7) (p=0.0488), Beta (B.1.351) (p=0.0010), Gamma (P.1) (p=0.0068) and Delta (B.1.617.2) (p=0.0068) (**fig. S12A**), but no statistical differences in endpoint titers analysis (**Fig.**

3Q). Differences in neutralizing antibody responses against VOCs by pseudovirus neutralization curve, including Alpha ($p=0.0156$), Beta ($p=0.0156$), and Delta ($p=0.0078$) **(fig. S12B)** were also observed, but no differences were observed in the pNT_{50} titer values for pseudovirus or authentic virus neutralization (**Fig. 3R and S, fig. S12C and D**), as compared to S_{FG}. These VOCs are of particular concern because they have shown resistance to several therapeutic antibodies and convalescent serum and because they have exhibited reduced protection by many approved vaccines (*14–18*).

S_{MG} vaccine provided superior protection against SARS-CoV-2 and variants in vivo.

To evaluate the in vivo protective efficacy of S_{MG} vaccine against SARS-CoV-2, we first carried out WT SARS-CoV-2 challenge in Syrian hamsters vaccinated with S_{MG} or S_{FG} **(Fig. 4A)**. S_{MG} vaccinated hamsters ($n = 5$) showed less reduction in body weight as compared to the S_{FG} and PBS groups **(Fig. 4B)**, whereas similar virus titer reductions were observed in the lungs of both S_{FG} and S_{MG} vaccinated hamsters **(Fig. 4C)**. Additionally, according to histopathological staining and anti-nucleocapsid (N) protein immunostaining data, fewer lesions were observed in the lungs of immunized hamsters **(Fig. 4D, fig. S13)**. Since hamsters only showed mild-to-moderate sickness upon SARS-CoV-2 infection, we then used severe disease models, the highly susceptible CAG-hACE2 (*19*) or K18-hACE2 (*20*) transgenic mice **(Fig. 4E)**. The analysis of anti-S IgG binding titer, neutralizing titers, anti-S subtype IgG, and IgG2c:IgG1 ratio **(Fig. 4F to I)** in CAG-hACE2 mice all showed similar results to the BALB/c mice (**Fig. 3C to G**). Following challenge with WT SARS-CoV-2 intranasally, virus was not detectable in the lungs of both S_{FG} and S_{MG} vaccinated CAG-hACE2-mice ($n=3$) by anti-N staining at 7 days post infection (dpi) **(Fig. 4J)** or median tissue culture infectious dose ($TCID_{50}$) assay at 4 dpi **(Fig. 4K)**, whereas a viral titer of over 1000 $TCID_{50}$ was observed in the control group (**Fig. 4K**). Importantly, the S_{MG} group ($n=4$) exhibited better (75%) survival rate than S_{FG} (50%) at 14 dpi **(Fig. 4L and M)**. We then evaluated the degree of protection conferred by S_{MG} vaccination against challenge with the Alpha variant in CAG-hACE2 mice ($n=5$). We found that S_{MG} vaccination provided 100% survival rate until 14 dpi **(Fig. 4N and O)**. S_{MG} vaccinated mice also showed a 60% survival rate in Gamma variant challenge in CAG-hACE2 mice ($n=5$) **(Fig. 4P and Q)** and a 75% survival rate in Delta variant challenge in K18-hACE2 mice ($n=4$) **(Fig. 4R and S)**, whereas less than 50% S_{FG} vaccinated mice survived until 14 dpi in Gamma and Delta variant challenge **(Fig. 4Q to S)**. The improved in vivo protection conferred by S_{MG} vaccination provides further evidence that removal of glycan shields from an immunogen is an advantageous strategy to elicit superior immune response.

A broadly neutralizing antibody was isolated from B cells of mice immunized with S_{MG}.

The sorting of S protein-specific B cells from S_{MG} immunized mice led to the identification of a monoclonal antibody (mAb) m31A7 from the *IGHV1-18* amplified clones, a subset that is uniquely abundant in S_{MG} immunized B cell repertoire **(Fig. 3O, fig. S11B)**. This mAb interacts with the full-length S protein, S1 and RBD, but not S2 **(Fig. 5A)** and binds to HEK293T cells that express the S protein from different SARS-CoV-2 variants (**Fig. 5B**). In addition, m31A7 was shown to neutralize various pseudovirus variants (WT, D614G, Alpha, Beta, and Delta) at sub-picomolar half maximal inhibitory concentration (IC_{50}), up to 1000-fold higher than the reported human mAb EY6A (*21*) **(Fig. 5C and fig. S14)**. A prophylactic study also demonstrated good in vivo efficacy of m31A7 in K18-hACE2 mice ($n=3$) challenged with WT SARS-CoV-2 **(Fig. 5D)**. Prophylactically treated mice maintained both body weight and temperature **(Fig. 5E and F)**. Bio-layer interferometry (BLI) analysis was used to measure the dissociation constants of m31A7 and its Fab binding to S protein at 70.9 pM and 4.66 nM, respectively (**Fig. 5G**). Epitope mapping by hydrogen-deuterium exchange mass spectrometry (HDX-MS) revealed its potential binding regions on RBD (**Fig. 5H, fig. S15**), which overlapped with the observed epitope in the crystal structure of RBD in complex with m31A7-Fab (**fig. S16**). The cryo-electron microscopy (EM) structure further clarified the binding of m31A7 only to RBD in the "up" state (**Fig. 5I**), with the N165-glycan from neighboring NTD in the vicinity of the RBD-m31A7 interface (**Fig. 5J**). The footprint of m31A7 on the RBD is similar to that of human *VH1-58* class mAbs (**Fig. 5K**) (*14*, *22*, *23*), but it approaches the RBD from a different angle, with shifted local contact areas on the tip loop, bypassing most of key mutated residues of VOCs such as E484 and K417, but not T478 **(Fig. 5L)** (*22*, *23*). The detailed RBD-m31A7 interface the inhibitory mechanism of this S_{MG}-elicited mAb is under further investigation.

DISCUSSION

Two years into the current global pandemic, a next-generation, broadly protective COVID-19 vaccine remains urgently needed. Although numerous studies have been published since the outbreak evaluating S protein glycosylation as well as different strategies for vaccine development, our work on a glycoengineered vaccine combines these two fields. The clinical translation of the current findings is promising, as protein-based vaccine platforms have been widely accepted with proven efficacy (*24*). Further, the enzymatic digestion process to remove unwanted glycans is straightforward and could be readily incorporated into current manufacturing procedures. In a parallel effort, a glycoengineered mRNA vaccine recently developed by our group also showed impressive results, supporting our design concept that uncovering

glycan-shielded epitopes led to a better vaccine with improved cross-protection (*25*).

A major concern at the present time is the inevitable emergence of new SARS-CoV-2 variants that carry immune-escape mutations (*14*, *26*). Notably, not a single N-glycosite mutation has been observed in any SARS-CoV-2 VOC identified so far; this is true even for the Omicron variant, which has more than 30 mutations in the S protein (*27*), again highlighting the importance of S protein glycosylation during viral evolution. Additionally, an interesting comparison can be made between our S_{MG} vaccine and other RBD-based vaccines, because S protein RBD, in its isolated form, is also barely covered by glycans (with the major coverage from N343). Nonetheless, the RBD contains several variation hotspots that are under stringent selective pressure, whereas our S_{MG} vaccine targets the entire S protein ectodomain, stimulating the elicitation of both RBD and non-RBD neutralizing antibodies that are critical for cross protection. Moreover, the improved $CD4^+$ and $CD8^+$ T cell responses elicited by S_{MG} vaccination represent an additional advantage for this protein-based vaccine, especially given the increasing evidence that T cell immunity plays a central role against SARS-CoV-2 infection (*28*).

A matter that may be raised about the glycan-removal design is that antibodies elicited by the mono-GlcNAc-decorated protein vaccine may fail to bind to the original glycosylated immunogen due to dramatic changes at the protein surface. This may have been the case in previous studies of HIV (*2*, *29*), as neutralizing antibodies that target Env protein gp120 (with 480 residues per protomer and about 25 N-glycosites) are facing a heavily glycosylated surface with few accessible protein epitopes. However, mono-GlcNAc-decorated protein vaccines have been shown to be advantageous for both influenza virus and SARS-CoV-2, probably because influenza hemagglutinin (H1N1 HA, with 560 residues per protomer and about 6 to 8 N-glycosites) (*10*) and S protein (with 1270 residues per protomer and 22 N-glycosites) are both low-to-moderately glycosylated. Additionally, these glycan chains are likely flexible enough to allow antibody binding even to the glycan-shielded, conserved epitopes.

We also recognized that there are some limitations of our present study. First, our experiments analyzing the impact of glycosites of S protein on interacting with ACE2, and the glycan profiling of S protein were done in human cell lines. It may be closer to the real scenario if primary lung epithelial cells, lung organoids, or other primary tissues expressing ACE2 can be employed. Second, a comprehensive analysis of the differential T cell repertoire remains to be identified, particularly of the CD8 T cell repertoire elicited by S_{FG} and S_{MG} vaccination. It will reveal further information on whether and how glycans on S protein may affect antigen presentation. Third, although we have portrayed the differential *IGHV, IGKV,* and *IGLV* locus usage by S_{FG} and S_{MG} vaccination, the importance of those differentially elicited B cell repertoires contributing to the potent protection by S_{MG} vaccination deserved further study. Other mAbs, particularly those belonging to *IGHV1-18* family, are worthwhile to be cloned and investigated. Finally, the strategy has only been validated in animal models, and remains to be confirmed in clinical trials.

In conclusion, SARS-CoV-2 S protein glycosylation has major influence on virus infection, protein integrity, and immune responses. The S protein from lung epithelial cells contained more sialylated complex-type glycans to facilitate receptor binding, and glycosites N801 and N1194 were shown to be essential for S protein folding and viral infection. The analysis of cell-specific glycoform distribution, sequence conservation, glycan shielding, and their mutual correlations, led to the design of S_{MG} vaccine, in which essentially all glycan shields are removed. This made the conserved epitopes better exposed to the immune system so that more effective and broadly protective B cell and T cell responses could be elicited against the virus and variants (*30*). As illustrated by the broadly neutralizing m31A7 antibody in this study and more to be identified in the future, the conserved epitopes targeted by such antibodies could be used for next-generation vaccine development. In an effort to develop vaccines against the currently circulating and future SARS-CoV-2 variants (*31*–*34*), the impact of glycosylation on viral infection, protein integrity, immune response and vaccine design as demonstrated in this study should be considered together with other parameters.

MATERIALS AND METHODS

Study design

This work aims to analyze the impact of glycosylation of SARS-CoV-2 S protein and investigate the efficiency of a glycoengineered Mono-GlcNAc-decorated S protein (S_{MG}) as a vaccine candidate. ACE2 binding ELISA and glycan-engineered pseudovirus neutralization assays were used to understand the impact of glycosylation on receptor binding and viral infectivity. Liquid chromatography coupled with tandem mass spectrometry (LC-MS/MS) was applied to analyze and compare glycan profiles of S protein expressed from different cell lines. Computational modelling and bioinformatic analysis were employed to visualize these findings on the 3D structure in the context of evolutionary conservation. The S_{MG} protein was designed, expressed, purified, characterized for glycan profiling, and injected into BALB/c mice for immune response analyses, including serum titers against SARS-CoV-2 WT and variants by ELISA and neutralization assay, T cell and B cell responses by flow cytometry, and B cell repertoire analysis by single-B cell screening assay. The in vivo protection was studied in both hamsters and hACE2 transgenic mice by weight monitoring, virus detection ($TCID_{50}$ and

histopathology and immunostaining analysis) and survival analysis. A broad-spectrum ultrapotent antibody m31A7 was identified from S_{MG} immunized mice, with its efficiency analyzed by ELISA, flow cytometry and pseudovirus neutralization, its protection tested in transgenic mice, binding affinity by BLI, epitope mapping by HDX-MS, and structure determination by cryo-EM and X-ray crystallography. Mouse or hamster group sizes were determined based on animal availability and power analyses using data from our pivotal experiments, and animals were randomly distributed between groups. No blinding was used throughout all studies, and no outliers were skewed to any one group. The selection of end points was made before the start of each study and decided based on the primary objective of investigating the immune responses to S_{MG} vaccination referring to previous studies (*12*, *19*, *20*). The number of technical or biological replicates varied between experiments as described in figure legends. These altogether serve as a foundation for further investigation and development of the $S_{MG.}$

SARS-CoV-2 S protein production and characterization

The production of S protein was modified from previous studies (*10*, *35*, *36*) with details in the supplementary materials and methods. pcDNA3.1 was used for construction and expression in human epithelial kidney (HEK) 293T cells (American Type Culture Collection (ATCC) CRL-3216) or BEAS-2B cells (*Homo sapiens*, lung) cells (ATCC CRL-9609). pTT was used for large-scale expression in HEK293 EBNA (ATCC CRL-10852) or HEK293S GnTI$^-$ (ATCC CRL-3022) suspension cells for vaccine studies. Purified S_{HM} (from HEK293S GnTI$^-$ cells) was further treated with Endo H (New England Biolabs, NEB) overnight at 25°C in a ratio of 50:1 (w/w) to produce S_{MG}. S protein purity was monitored by SDS-PAGE, with its binding avidity to hACE2 evaluated by ELISA, its glycan profile analyzed by LC-MS/MS (glycoform categorization followed a previous report (*2*)), and its structural conformation confirmed by negative staining EM (**fig. S10G and H**).

Pseudovirus production with glycan-engineered S protein and glycosite-specific S mutants

The production of glycan-engineered pseudoviruses in (**Fig. 1D**) followed previous studies (*2*, *6*). For production of glycosite-specific S mutant pseudoviruses (**Fig. 1F**), HEK-293T cells were transiently transfected with pVax-nCoV-S△19 construct carrying mutations at each glycosite and luciferase-expressing HIV-1 genome plasmid (pNL4-3.luc.RE). Details of pseudovirus production and infectivity assay can be found in supplementary materials and methods.

Informatic analysis of SARS-CoV-2 S protein

1,117,474 S protein sequences of all available SARS-CoV-2 strains were extracted from the GISAID (Global Initiative on Sharing Avian Influenza Data) database (version: Apr. 18, 2021) (*9*). The S-protein 3D structure modeling was constructed by CHARMM-GUI (*37*) and OpenMM (*38*) based on the PDB file 6VSB_1_1_1 (*39*) with the most abundant glycoform of BEAS-2B data as representative glycan profile (**fig. S4**). *O*-glycans (T323 and S325) used Neu5Ac (α2,3)Gal (β1,3)GalNAc (α1) as representative (*8*). The definition of transmembrane region was according to UniProt (P0DTC2) and other parameters in CHARMM-GUI were according to Woo, H. *et al.* (*39*). Scripts, parameters, and pre-optimized models generated by CHARMM-GUI were used as the input for OpenMM. The protein secondary structure was determined by majority voting in the Dictionary of Secondary Structure of Proteins (DSSP) program (*40*, *41*) and 2Struc web server (*42*). The relative solvent accessibility (RSA) of S protein was calculated by FreeSASA program (*43*) and averaged at each site of residues from three chains. The probe radius in FreeSASA was set to 7.2Å to approximate the average size of hypervariable loops of antibody complementarity determining regions (CDRs) (*44*). Residues with RSA above 5% were regarded as exposed; otherwise as buried (*45*). All 3D structure figures were drawn by UCSF ChimeraX (*46*).

Animal immunization, virus challenge, and prophylactic study of m31A7

For mice vaccination with 2-dose schedule, female 6- to 8-week-old BALB/c mice (n=5) were immunized intramuscularly with 10 μg purified S_{FG}, $S_{HM,}$ or S_{MG} mixed with aluminum hydroxide (50 μg) at day 0 and day 14. The serum was collected at day 28 after the first vaccination for evaluation of anti-S IgG abundance, IgG subtype, and neutralizing titers (described in the supplementary materials and methods). The lymph nodes of S_{FG} or S_{MG} immunized mice were collected at day 21 after the first vaccination for T cell response analysis (described in the supplementary materials and methods). For B cell repertoire analysis and serum titers against variants, female 6- to 8-week-old BALB/c mice (n=5) were immunized intramuscularly with 20 μg purified S_{FG} or S_{MG} mixed with aluminum hydroxide (20 μg) at day 0, day 14, and day 56; mice were euthanized at day 84 to collect whole blood for anti-S IgG and neutralizing titer evaluation and spleens for sorting of S protein-specific B cells (described in the supplementary materials and methods).

For hamster vaccination and virus challenge study, male 6- to 7-week-old Golden Syrian Hamsters (n=5) were immunized intramuscularly with 25 μg of purified S_{FG} or S_{MG} mixed with aluminum hydroxide (250 μg) at day 0 and day 14. Four weeks after the second immunization, each hamster was intranasally challenged with 1×10^4 $TCID_{50}$ of SARS-CoV-2 (hCoV-19/Taiwan/4/2020) in 100 μl PBS. Body weight was recorded daily after infection. On day 3 after challenge, hamsters were euthanized by carbon dioxide. The superior lobe of left lung was fixed in 10% paraformaldehyde for histopathological examination and the rest of lung was collected for

viral load determination ($TCID_{50}$ assay) as described in the supplementary materials and methods.

For transgenic mouse vaccination and virus challenge study, male 6 to 8-week-old CAG-hACE2 transgenic mice (*19*) or male 12-week-old K18-hACE2 transgenic mice (*20*) (purchased from Jackson Laboratory) were immunized intramuscularly with 10 μg of purified S_{FG} or S_{MG} mixed with aluminum hydroxide (50 μg) at day 0 and day 14. CAG-hACE2 transgenic mice were challenged intranasally 4 weeks after the second immunization with 1×10^3 $TCID_{50}$ of WT SARS-CoV-2. In the first trial (n=3), all mice were euthanized at 7 dpi for histopathological examination of superior lobe of left lung; in the second trial, (n=7), 3 mice were euthanized at 4 dpi for lung virus titer and 4 mice were kept until 14 dpi for survival analysis. Serum was collected 1 day before virus challenge.

For challenge studies using variants of concern, CAG-hACE2 mice were challenge with 1×10^3 $TCID_{50}$ of the Alpha variant (hCoV-19/Taiwan/792/2020) (n=5) or the Gamma variant (hCoV-19/Taiwan/906/2021) of SARS-CoV-2 in 50 μl PBS per mouse. Additionally, K18-hACE2 mice were challenged intranasally 4 weeks after the second immunization with 1×10^4 $TCID_{50}$ of Delta SARS-CoV-2 (hCoV-19/Taiwan/1144/2021) (n=4) in 50 μL PBS per mice. For all SARS-CoV-2 variant challenge models, body weight for each mouse was recorded daily until 14 dpi.

For prophylactic protection test of antibody, male 8-week-old K18-hACE2 transgenic mice (n=3) were injected intraperitoneally with m31A7 (15 mg/kg) or PBS 1 day before being intranasally challenged with 1×10^3 $TCID_{50}$ of WT SARS-CoV-2 (hCoV-19/Taiwan/4/2020). Body weight and body temperature were recorded daily until 5 dpi. All animal experiments were evaluated and approved by the Institutional Animal Care and Use Committee of Academia Sinica (approval no. 21-10-1716, 18-12-1272 and 20-10-1522).

m31A7 isolation by single-B cell screening assay and characterization

Primers were designed based on a previous publication (*47*). Polymerase chain reaction (PCR) was performed at 50°C for 30 min, 95°C for 15 min followed by 40 cycles of incubation at 94°C for 30 s, 50°C for 30 s and 72°C for 1 min, with a final extension at 72°C for 10 min. Semi-nested second round PCR was performed using KOD One PCR master mix (TOYOBO) with 1 μl of unpurified first round PCR product at 98°C for 2 min followed by 45 cycles of incubation at 98°C for 10 s, 55°C for 10 s and 68°C for 10 s, with a final extension at 68°C for 1 min. PCR products were then analyzed by electrophoresis and sequencing. The Ig V and L genes were identified on IMGT (the international ImMunoGeneTics information system, http://imgt.org/IMGT_vquest/input). Genes were then amplified from second round PCR product with single gene-specific V and L gene primers containing restriction sites for cloning into the vectors containing human IgH or IgL expression backbone. The chimeric IgH and IgL expression constructs were co-transfected into Expi293 for antibody production. After m31A7 was isolated, the antibody was subsequently evaluated for S protein binding by ELISA and FACS (fluorescence activated cell sorting), pseudovirus neutralization potency, binding kinetics, epitope mapping, and structure determination. Details are in the supplementary materials and methods.

Statistical analysis.

Raw data can be found in data file S1. All data were expressed as the means ± standard errors of the means (SEM) or standard deviation (SD) as mentioned individually. In **Fig. 1D**, ordinary one-way ANOVA test followed by Tukey's multiple comparisons was used to compare data. In **Fig. 5H**, multiple *t* tests with the two-stage step-up method of Benjamini, Krieger and Yekutieli multiple comparisons was used to compare each time point. For B cell repertoire analysis, a Chi-Squared test was used. For all serum antibody titer analyses, *P* values were obtained from two-sided Mann–Whitney *U*-tests to compare two experimental groups. Curves were fit by nonlinear regression using Graph Prism 9.0 and comparisons were performed by Wilcoxon matched-pairs signed rank test (two-tailed). $P < 0.05$ was considered statistically significant. $*P < 0.05$; $**P < 0.01$; $***P < 0.001$; $****P < 0.0001$.

SUPPLEMENTARY MATERIALS

www.science.org/doi/10.1126/scitranslmed.abm0899
Supplementary Materials and Methods
Figs. S1 to S16
Tables S1 to S3
References (*48*–*52*)
MDAR Reproducibility Checklist
Data file S1

REFERENCES AND NOTES

1. P. J. Klasse, D. F. Nixon, J. P. Moore, Immunogenicity of clinically relevant SARS-CoV-2 vaccines in nonhuman primates and humans. *Sci. Adv.* 7, eabe8065 (2021). doi:10.1126/sciadv.abe8065 Medline
2. Y. Watanabe, J. D. Allen, D. Wrapp, J. S. McLellan, M. Crispin, Site-specific glycan analysis of the SARS-CoV-2 spike. *Science* 369, 330–333 (2020). doi:10.1126/science.abb9983 Medline
3. P. Zhao, J. L. Praissman, O. C. Grant, Y. Cai, T. Xiao, K. E. Rosenbalm, K. Aoki, B. P. Kellman, R. Bridger, D. H. Barouch, M. A. Brindley, N. E. Lewis, M. Tiemeyer, B. Chen, R. J. Woods, L. Wells, Virus-Receptor Interactions of Glycosylated SARS-CoV-2 Spike and Human ACE2 Receptor. *Cell Host Microbe* 28, 586–601.e6 (2020). doi:10.1016/j.chom.2020.08.004 Medline
4. J. D. Allen, H. Chawla, F. Samsudin, L. Zuzic, A. T. Shivgan, Y. Watanabe, W.-T. He, S. Callaghan, G. Song, P. Yong, P. J. M. Brouwer, Y. Song, Y. Cai, H. M. E. Duyvesteyn, T. Malinauskas, J. Kint, P. Pino, M. J. Wurm, M. Frank, B. Chen, D. I. Stuart, R. W. Sanders, R. Andrabi, D. R. Burton, S. Li, P. J. Bond, M. Crispin, Site-Specific Steric Control of SARS-CoV-2 Spike Glycosylation. *Biochemistry* 60, 2153–2169 (2021). doi:10.1021/acs.biochem.1c00279 Medline
5. K. M. Bouwman, I. Tomris, H. L. Turner, R. van der Woude, T. M. Shamorkina, G. P. Bosman, B. Rockx, S. Herfst, J. Snijder, B. L. Haagmans, A. B. Ward, G.-J. Boons, R. P. de Vries, Multimerization- and glycosylation-dependent receptor binding of SARS-CoV-2 spike proteins. *PLOS Pathog.* 17, e1009282–e1009282 (2021). doi:10.1371/journal.ppat.1009282 Medline
6. Q. Yang, T. A. Hughes, A. Kelkar, X. Yu, K. Cheng, S. Park, W.-C. Huang, J. F. Lovell, S. Neelamegham, Inhibition of SARS-CoV-2 viral entry upon blocking N- and O-glycan elaboration. *eLife* 9, e61552 (2020). doi:10.7554/eLife.61552 Medline

7. Q. Li, J. Wu, J. Nie, L. Zhang, H. Hao, S. Liu, C. Zhao, Q. Zhang, H. Liu, L. Nie, H. Qin, M. Wang, Q. Lu, X. Li, Q. Sun, J. Liu, L. Zhang, X. Li, W. Huang, Y. Wang, The Impact of Mutations in SARS-CoV-2 Spike on Viral Infectivity and Antigenicity. *Cell* **182**, 1284–1294.e9 (2020). doi:10.1016/j.cell.2020.07.012 Medline
8. L. Casalino, Z. Gaieb, J. A. Goldsmith, C. K. Hjorth, A. C. Dommer, A. M. Harbison, C. A. Fogarty, E. P. Barros, B. C. Taylor, J. S. McLellan, E. Fadda, R. E. Amaro, Beyond Shielding: The Roles of Glycans in the SARS-CoV-2 Spike Protein. *ACS Cent. Sci.* **6**, 1722–1734 (2020). doi:10.1021/acscentsci.0c01056 Medline
9. S. Elbe, G. Buckland-Merrett, Data, disease and diplomacy: GISAID's innovative contribution to global health. *Glob. Chall.* **1**, 33–46 (2017). doi:10.1002/gch2.1018 Medline
10. J.-R. Chen, Y.-H. Yu, Y.-C. Tseng, W.-L. Chiang, M.-F. Chiang, Y.-A. Ko, Y.-K. Chiu, H.-H. Ma, C.-Y. Wu, J.-T. Jan, K.-I. Lin, C. Ma, C.-H. Wong, Vaccination of monoglycosylated hemagglutinin induces cross-strain protection against influenza virus infections. *Proc. Natl. Acad. Sci. U.S.A.* **111**, 2476–2481 (2014). doi:10.1073/pnas.1323954111 Medline
11. C.-C. Wang, J.-R. Chen, Y.-C. Tseng, C.-H. Hsu, Y.-F. Hung, S.-W. Chen, C.-M. Chen, K.-H. Khoo, T.-J. Cheng, Y.-S. E. Cheng, J.-T. Jan, C.-Y. Wu, C. Ma, C.-H. Wong, Glycans on influenza hemagglutinin affect receptor binding and immune response. *Proc. Natl. Acad. Sci. U.S.A.* **106**, 18137–18142 (2009). doi:10.1073/pnas.0909696106 Medline
12. T.-Y. Kuo, M.-Y. Lin, R. L. Coffman, J. D. Campbell, P. Traquina, Y.-J. Lin, L. T.-C. Liu, J. Cheng, Y.-C. Wu, C.-C. Wu, W.-H. Tang, C.-G. Huang, K.-C. Tsao, C. Chen, Development of CpG-adjuvanted stable prefusion SARS-CoV-2 spike antigen as a subunit vaccine against COVID-19. *Sci. Rep.* **10**, 20085–20085 (2020). doi:10.1038/s41598-020-77077-z Medline
13. P. R. Krause, T. R. Fleming, I. M. Longini, R. Peto, S. Briand, D. L. Heymann, V. Beral, M. D. Snape, H. Rees, A.-M. Ropero, R. D. Balicer, J. P. Cramer, C. Muñoz-Fontela, M. Gruber, R. Gaspar, J. A. Singh, K. Subbarao, M. D. Van Kerkhove, S. Swaminathan, M. J. Ryan, A.-M. Henao-Restrepo, SARS-CoV-2 Variants and Vaccines. *N. Engl. J. Med.* **385**, 179–186 (2021). doi:10.1056/NEJMsr2105280 Medline
14. W. T. Harvey, A. M. Carabelli, B. Jackson, R. K. Gupta, E. C. Thomson, E. M. Harrison, C. Ludden, R. Reeve, A. Rambaut, S. J. Peacock, D. L. Robertson; COVID-19 Genomics UK (COG-UK) Consortium, SARS-CoV-2 variants, spike mutations and immune escape. *Nat. Rev. Microbiol.* **19**, 409–424 (2021). doi:10.1038/s41579-021-00573-0 Medline
15. W. F. Garcia-Beltran, E. C. Lam, K. St Denis, A. D. Nitido, Z. H. Garcia, B. M. Hauser, J. Feldman, M. N. Pavlovic, D. J. Gregory, M. C. Poznansky, A. Sigal, A. G. Schmidt, A. J. Iafrate, V. Naranbhai, A. B. Balazs, Multiple SARS-CoV-2 variants escape neutralization by vaccine-induced humoral immunity. *Cell* **184**, 2372–2383.e9 (2021). doi:10.1016/j.cell.2021.03.013 Medline
16. G.-L. Wang, Z.-Y. Wang, L.-J. Duan, Q.-C. Meng, M.-D. Jiang, J. Cao, L. Yao, K.-L. Zhu, W.-C. Cao, M.-J. Ma, Susceptibility of Circulating SARS-CoV-2 Variants to Neutralization. *N. Engl. J. Med.* **384**, 2354–2356 (2021). doi:10.1056/NEJMc2103022 Medline
17. J. Lopez Bernal, N. Andrews, C. Gower, E. Gallagher, R. Simmons, S. Thelwall, J. Stowe, E. Tessier, N. Groves, G. Dabrera, R. Myers, C. N. J. Campbell, G. Amirthalingam, M. Edmunds, M. Zambon, K. E. Brown, S. Hopkins, M. Chand, M. Ramsay, Effectiveness of Covid-19 Vaccines against the B.1.617.2 (Delta) Variant. *N. Engl. J. Med.* **385**, 585–594 (2021). doi:10.1056/NEJMoa2108891 Medline
18. D. Corti, L. A. Purcell, G. Snell, D. Veesler, Tackling COVID-19 with neutralizing monoclonal antibodies. *Cell* **184**, 3086–3108 (2021). doi:10.1016/j.cell.2021.05.005 Medline
19. C.-Y. Tsai, C.-Y. Chen, J.-T. Jan, Y.-C. Chou, M.-L. Chang, L. A. Lu, P.-Y. Huang, M. F. C. Chu, T.-T. Hsu, Y.-P. Hsueh, Sex-biased response to and brain cell infection by SARS-CoV-2 in a highly susceptible human ACE2 transgenic model. *bioRxiv*, 2021.2005.2004.441029 (2021)10.1101/2021.05.04.441029).
20. E. S. Winkler, A. L. Bailey, N. M. Kafai, S. Nair, B. T. McCune, J. Yu, J. M. Fox, R. E. Chen, J. T. Earnest, S. P. Keeler, J. H. Ritter, L.-I. Kang, S. Dort, A. Robichaud, R. Head, M. J. Holtzman, M. S. Diamond, SARS-CoV-2 infection of human ACE2-transgenic mice causes severe lung inflammation and impaired function. *Nat. Immunol.* **21**, 1327–1335 (2020). doi:10.1038/s41590-020-0778-2 Medline
21. D. Zhou, H. M. E. Duyvesteyn, C.-P. Chen, C.-G. Huang, T.-H. Chen, S.-R. Shih, Y.-C. Lin, C.-Y. Cheng, S.-H. Cheng, Y.-C. Huang, T.-Y. Lin, C. Ma, J. Huo, L. Carrique, T. Malinauskas, R. R. Ruza, P. N. M. Shah, T. K. Tan, P. Rijal, R. F. Donat, K. Godwin, K. R. Buttigieg, J. A. Tree, J. Radecke, N. G. Paterson, P. Supasa, J. Mongkolsapaya, G. R. Screaton, M. W. Carroll, J. Gilbert-Jaramillo, M. L. Knight, W. James, R. J. Owens, J. H. Naismith, A. R. Townsend, E. E. Fry, Y. Zhao, J. Ren, D. I. Stuart, K. A. Huang, Structural basis for the neutralization of SARS-CoV-2 by an antibody from a convalescent patient. *Nat. Struct. Mol. Biol.* **27**, 950–958 (2020). doi:10.1038/s41594-020-0480-y Medline
22. L. Wang, T. Zhou, Y. Zhang, E. S. Yang, C. A. Schramm, W. Shi, A. Pegu, O. K. Oloniniyi, A. R. Henry, S. Darko, S. R. Narpala, C. Hatcher, D. R. Martinez, Y. Tsybovsky, E. Phung, O. M. Abiona, A. Antia, E. M. Cale, L. A. Chang, M. Choe, K. S. Corbett, R. L. Davis, A. T. DiPiazza, I. J. Gordon, S. H. Hait, T. Hermanus, P. Kgagudi, F. Laboune, K. Leung, T. Liu, R. D. Mason, A. F. Nazzari, L. Novik, S. O'Connell, S. O'Dell, A. S. Olia, S. D. Schmidt, T. Stephens, C. D. Stringham, C. A. Talana, I. T. Teng, D. A. Wagner, A. T. Widge, B. Zhang, M. Roederer, J. E. Ledgerwood, T. J. Ruckwardt, M. R. Gaudinski, P. L. Moore, N. A. Doria-Rose, R. S. Baric, B. S. Graham, A. B. McDermott, D. C. Douek, P. D. Kwong, J. R. Mascola, N. J. Sullivan, J. Misasi, Ultrapotent antibodies against diverse and highly transmissible SARS-CoV-2 variants. *Science* **373**, eabh1766 (2021). doi:10.1126/science.abh1766 Medline
23. W. Dejnirattisai, D. Zhou, H. M. Ginn, H. M. E. Duyvesteyn, P. Supasa, J. B. Case, Y. Zhao, T. S. Walter, A. J. Mentzer, C. Liu, B. Wang, G. C. Paesen, J. Slon-Campos, C. López-Camacho, N. M. Kafai, A. L. Bailey, R. E. Chen, B. Ying, C. Thompson, J. Bolton, A. Fyfe, S. Gupta, T. K. Tan, J. Gilbert-Jaramillo, W. James, M. Knight, M. W. Carroll, D. Skelly, C. Dold, Y. Peng, R. Levin, T. Dong, A. J. Pollard, J. C. Knight, P. Klenerman, N. Temperton, D. R. Hall, M. A. Williams, N. G. Paterson, F. K. R. Bertram, C. A. Siebert, D. K. Clare, A. Howe, J. Radecke, Y. Song, A. R. Townsend, K. A. Huang, E. E. Fry, J. Mongkolsapaya, M. S. Diamond, J. Ren, D. I. Stuart, G. R. Screaton, The antigenic anatomy of SARS-CoV-2 receptor binding domain. *Cell* **184**, 2183–2200.e22 (2021). doi:10.1016/j.cell.2021.02.032 Medline
24. Y. Li, R. Tenchov, J. Smoot, C. Liu, S. Watkins, Q. Zhou, A Comprehensive Review of the Global Efforts on COVID-19 Vaccine Development. *ACS Cent. Sci.* **7**, 512–533 (2021). doi:10.1021/acscentsci.1c00120 Medline
25. C.-Y. Wu, C.-W. Cheng, C.-C. Kung, K.-S. Liao, J.-T. Jan, C. Ma, C.-H. Wong, Glycosite-deleted mRNA of SARS-CoV-2 spike protein as a broad-spectrum vaccine. *Proc. Natl. Acad. Sci. U.S.A.* **119**, e2119995119 (2022). doi:10.1073/pnas.2119995119 Medline
26. L. A. VanBlargan, J. M. Errico, P. J. Halfmann, S. J. Zost, J. E. Crowe Jr., L. A. Purcell, Y. Kawaoka, D. Corti, D. H. Fremont, M. S. Diamond, An infectious SARS-CoV-2 B.1.1.529 Omicron virus escapes neutralization by therapeutic monoclonal antibodies. *Nat. Med.* (2022). doi:10.1038/s41591-021-01678-y Medline
27. M. Stravalaci, I. Pagani, E. M. Paraboschi, M. Pedotti, A. Doni, F. Scavello, S. N. Mapelli, M. Sironi, C. Perucchini, L. Varani, M. Matkovic, A. Cavalli, D. Cesana, P. Gallina, N. Pedemonte, V. Capurro, N. Clementi, N. Mancini, P. Invernizzi, R. Bayarri-Olmos, P. Garred, R. Rappuoli, S. Duga, B. Bottazzi, M. Uguccioni, R. Asselta, E. Vicenzi, A. Mantovani, C. Garlanda, Recognition and inhibition of SARS-CoV-2 by humoral innate immunity pattern recognition molecules. *Nat. Immunol.* **23**, 275–286 (2022). doi:10.1038/s41590-021-01114-w Medline
28. P. Moss, The T cell immune response against SARS-CoV-2. *Nat. Immunol.* **23**, 186–193 (2022). doi:10.1038/s41590-021-01122-w Medline
29. K. Wagh, B. H. Hahn, B. Korber, Hitting the sweet spot: Exploiting HIV-1 glycan shield for induction of broadly neutralizing antibodies. *Curr. Opin. HIV AIDS* **15**, 267–274 (2020). doi:10.1097/COH.0000000000000639 Medline
30. M. Sadarangani, A. Marchant, T. R. Kollmann, Immunological mechanisms of vaccine-induced protection against COVID-19 in humans. *Nat. Rev. Immunol.* **21**, 475–484 (2021). doi:10.1038/s41577-021-00578-z Medline
31. D. R. Martinez, A. Schäfer, S. R. Leist, G. De la Cruz, A. West, E. N. Atochina-Vasserman, L. C. Lindesmith, N. Pardi, R. Parks, M. Barr, D. Li, B. Yount, K. O. Saunders, D. Weissman, B. F. Haynes, S. A. Montgomery, R. S. Baric, Chimeric spike mRNA vaccines protect against Sarbecovirus challenge in mice. *Science* **373**, 991–998 (2021). doi:10.1126/science.abi4506 Medline
32. A. A. Cohen, P. N. P. Gnanapragasam, Y. E. Lee, P. R. Hoffman, S. Ou, L. M. Kakutani, J. R. Keeffe, H. J. Wu, M. Howarth, A. P. West, C. O. Barnes, M. C. Nussenzweig, P. J. Bjorkman, Mosaic nanoparticles elicit cross-reactive immune responses to zoonotic coronaviruses in mice. *Science* **371**, 735–741 (2021). doi:10.1126/science.abf6840 Medline

33. K. O. Saunders, E. Lee, R. Parks, D. R. Martinez, D. Li, H. Chen, R. J. Edwards, S. Gobeil, M. Barr, K. Mansouri, S. M. Alam, L. L. Sutherland, F. Cai, A. M. Sanzone, M. Berry, K. Manne, K. W. Bock, M. Minai, B. M. Nagata, A. B. Kapingidza, M. Azoitei, L. V. Tse, T. D. Scobey, R. L. Spreng, R. W. Rountree, C. T. DeMarco, T. N. Denny, C. W. Woods, E. W. Petzold, J. Tang, T. H. Oguin 3rd, G. D. Sempowski, M. Gagne, D. C. Douek, M. A. Tomai, C. B. Fox, R. Seder, K. Wiehe, D. Weissman, N. Pardi, H. Golding, S. Khurana, P. Acharya, H. Andersen, M. G. Lewis, I. N. Moore, D. C. Montefiori, R. S. Baric, B. F. Haynes, Neutralizing antibody vaccine for pandemic and pre-emergent coronaviruses. *Nature* **594**, 553–559 (2021). doi:10.1038/s41586-021-03594-0 Medline
34. A. E. Powell, K. Zhang, M. Sanyal, S. Tang, P. A. Weidenbacher, S. Li, T. D. Pham, J. E. Pak, W. Chiu, P. S. Kim, A Single Immunization with Spike-Functionalized Ferritin Vaccines Elicits Neutralizing Antibody Responses against SARS-CoV-2 in Mice. *ACS Cent. Sci.* **7**, 183–199 (2021). doi:10.1021/acscentsci.0c01405 Medline
35. K. Papanikolopoulou, M. J. van Raaij, A. Mitraki, Creation of hybrid nanorods from sequences of natural trimeric fibrous proteins using the fibritin trimerization motif. *Methods Mol. Biol.* **474**, 15–33 (2008). doi:10.1007/978-1-59745-480-3_2 Medline
36. D. Wrapp, N. Wang, K. S. Corbett, J. A. Goldsmith, C.-L. Hsieh, O. Abiona, B. S. Graham, J. S. McLellan, Cryo-EM structure of the 2019-nCoV spike in the prefusion conformation. *Science* **367**, 1260–1263 (2020). doi:10.1126/science.abb2507 Medline
37. S.-J. Park, J. Lee, Y. Qi, N. R. Kern, H. S. Lee, S. Jo, I. Joung, K. Joo, J. Lee, W. Im, CHARMM-GUI Glycan Modeler for modeling and simulation of carbohydrates and glycoconjugates. *Glycobiology* **29**, 320–331 (2019). doi:10.1093/glycob/cwz003 Medline
38. P. Eastman, J. Swails, J. D. Chodera, R. T. McGibbon, Y. Zhao, K. A. Beauchamp, L.-P. Wang, A. C. Simmonett, M. P. Harrigan, C. D. Stern, R. P. Wiewiora, B. R. Brooks, V. S. Pande, OpenMM 7: Rapid development of high performance algorithms for molecular dynamics. *PLOS Comput. Biol.* **13**, e1005659–e1005659 (2017). doi:10.1371/journal.pcbi.1005659 Medline
39. H. Woo, S.-J. Park, Y. K. Choi, T. Park, M. Tanveer, Y. Cao, N. R. Kern, J. Lee, M. S. Yeom, T. I. Croll, C. Seok, W. Im, Developing a Fully Glycosylated Full-Length SARS-CoV-2 Spike Protein Model in a Viral Membrane. *J. Phys. Chem. B* **124**, 7128–7137 (2020). doi:10.1021/acs.jpcb.0c04553 Medline
40. W. Kabsch, C. Sander, Dictionary of protein secondary structure: Pattern recognition of hydrogen-bonded and geometrical features. *Biopolymers* **22**, 2577–2637 (1983). doi:10.1002/bip.360221211 Medline
41. W. G. Touw, C. Baakman, J. Black, T. A. H. te Beek, E. Krieger, R. P. Joosten, G. Vriend, A series of PDB-related databanks for everyday needs. *Nucleic Acids Res.* **43** (D1), D364–D368 (2015). doi:10.1093/nar/gku1028 Medline
42. D. P. Klose, B. A. Wallace, R. W. Janes, 2Struc: The secondary structure server. *Bioinformatics* **26**, 2624–2625 (2010). doi:10.1093/bioinformatics/btq480 Medline
43. S. Mitternacht, FreeSASA: An open source C library for solvent accessible surface area calculations. *F1000 Res.* **5**, 189–189 (2016). doi:10.12688/f1000research.7931.1 Medline
44. O. C. Grant, D. Montgomery, K. Ito, R. J. Woods, Analysis of the SARS-CoV-2 spike protein glycan shield reveals implications for immune recognition. *Sci. Rep.* **10**, 14991–14991 (2020). doi:10.1038/s41598-020-71748-7 Medline
45. T. Kajander, P. C. Kahn, S. H. Passila, D. C. Cohen, L. Lehtiö, W. Adolfsen, J. Warwicker, U. Schell, A. Goldman, Buried charged surface in proteins. *Structure* **8**, 1203–1214 (2000). doi:10.1016/S0969-2126(00)00520-7 Medline
46. E. F. Pettersen, T. D. Goddard, C. C. Huang, E. C. Meng, G. S. Couch, T. I. Croll, J. H. Morris, T. E. Ferrin, UCSF ChimeraX: Structure visualization for researchers, educators, and developers. *Protein Sci.* **30**, 70–82 (2021). doi:10.1002/pro.3943 Medline
47. T. Tiller, C. E. Busse, H. Wardemann, Cloning and expression of murine Ig genes from single B cells. *J. Immunol. Methods* **350**, 183–193 (2009). doi:10.1016/j.jim.2009.08.009 Medline
48. T. Grant, A. Rohou, N. Grigorieff, *cis*TEM, user-friendly software for single-particle image processing. *eLife* **7**, e35383 (2018). doi:10.7554/eLife.35383 Medline
49. S. H. W. Scheres, RELION: Implementation of a Bayesian approach to cryo-EM structure determination. *J. Struct. Biol.* **180**, 519–530 (2012). doi:10.1016/j.jsb.2012.09.006 Medline
50. T. G. Battye, L. Kontogiannis, O. Johnson, H. R. Powell, A. G. Leslie, iMOSFLM: A new graphical interface for diffraction-image processing with MOSFLM. *Acta Crystallogr. D Biol. Crystallogr.* **67**, 271–281 (2011). doi:10.1107/S0907444910048675 Medline
51. D. Liebschner, P. V. Afonine, M. L. Baker, G. Bunkóczi, V. B. Chen, T. I. Croll, B. Hintze, L. W. Hung, S. Jain, A. J. McCoy, N. W. Moriarty, R. D. Oeffner, B. K. Poon, M. G. Prisant, R. J. Read, J. S. Richardson, D. C. Richardson, M. D. Sammito, O. V. Sobolev, D. H. Stockwell, T. C. Terwilliger, A. G. Urzhumtsev, L. L. Videau, C. J. Williams, P. D. Adams, Macromolecular structure determination using X-rays, neutrons and electrons: Recent developments in Phenix. *Acta Crystallogr. D Struct. Biol.* **75**, 861–877 (2019). doi:10.1107/S2059798319011471 Medline
52. P. Emsley, B. Lohkamp, W. G. Scott, K. Cowtan, Features and development of Coot. *Acta Crystallogr. D Biol. Crystallogr.* **66**, 486–501 (2010). doi:10.1107/S0907444910007493 Medline

Acknowledgments: We thank the Academia Sinica specific-pathogen-free (SPF) Animal Facility for providing animal support and the National RNAi Core Facility at Academia Sinica in Taiwan for providing pseudovirus reagents and related services. We thank the Mass Core Facility of Genomics Research Center, Academia Sinica for glycopeptide LC-MS/MS analysis and the Academia Sinica Proteomics Common Mass Spectrometry Facilities in the Institute of Biological Chemistry for HDX-MS. We thank the technical services provided by the National Synchrotron Radiation Research Center, Taiwan. We thank Chun-Kai Chang for helping with hamster vaccination, Chia-Wei Li and Hsiang-Chi Huang for providing ACE2-expressing cell lines, Kuan-Ying A. Huang for providing antibody EY6A, and Chia-Yen Chen and Kuang-Cheng Lee for glycoform analysis. **Funding:** The research was funded by Genomics Research Center, Summit Project (AS-SUMMIT-109) and the Translational Medical Research Program (AS-KPQ-109-BioMed), Academia Sinica and Taiwan Bio-Development Foundation (to C.M.). The Academia Sinica SPF Animal Facility, the Academia Sinica Transgenic Core Facility in the Institute of Molecular Biology, the Academia Sinica Biological Electron Microscopy Core Facility in the Institute of Cellular and Organismic Biology and the Academia Sinica Proteomics Mass Spectrometry Facilities in the Institute of Biological Chemistry were funded by Academia Sinica Core Facility and Innovative Instrument Projects (AS-CFII-111-204, AS-CFII-108-104, AS-CFII-108-119, and AS-CFII-108-107, respectively). The Academia Sinica Cryo-EM Facility (ASCEM) was funded by Academia Sinica Core Facility and Innovative Instrument Projects (AS-CFII-108-110) and Taiwan Protein Project (AS-KPQ-109-TPP2). The Biosafety Level 3 Laboratory of the First Core Laboratory from National Taiwan University College of Medicine was supported by the Ministry of Science and Technology (MOST-110-2740-B-002-006, MOST-109-2327-B-002-009). **Author contributions:** C.M. and C.H.W. conceived the idea. H.Y.L., C.Y.W., H.Y.H and Y.C.C. performed pseudovirus production, infectivity or neutralization. H.Y.L. and T.-J.R.C performed glycan profile analysis. C.W.C. performed bioinformatic analysis. H.Y.H., X.C., T.H.C., and J.M.L performed S protein vaccine design and preparation. H.Y.H. performed immunization, serum collection, and vaccine analysis. J.T.J., H.H.M., Y.M.L., S.Y.C., T.L.C., H.C.K., and Y.M.T. performed virus challenge animal studies and authentic virus neutralization in biosafety level 3. M.S.A.-M. and Y.H.Chen performed IHC and IF staining. A.M., X.C. and M.S.A.-M. purified S protein variants. S.W.W., Y.H.Chang and H.Y.T. performed T cell analysis, B cell sorting, m31A7 identification, and antibody analysis. Y.P.H., C.Y.T. and P.Y.H. generated and immunized CAG-hACE2 mice. X.C., Y.M.W. and M.S.A.-M. performed cryo-EM structure determination. A.M. performed crystal structure determination. C.H.W., C.M., K.I.L., H.Y.H., X.C., S.W.W., H.Y.L., and C.W.C. wrote the paper. **Competing interests:** C.M., C.H.W., H.Y.H. are inventors on patent application (63/190,199) submitted by Academia Sinica that covers improved coronavirus vaccines. K.I.L., C.M., C.H.W., S.W.W., Y.H.Chang, X.C., and H.Y.H. are inventors on patent application (63/251,472) submitted by Academia Sinica entitled "A chimera monoclonal antibody possesses protective activity against various SARS-CoV-2 variants." All other authors declared no competing interests. **Data and materials availability**: All data associated with this study are in the paper or supplementary materials. Negative stain volume of S_{MG} was deposited in the Electron Microscopy Data Bank (EMD) under accession number EMD-32825; Cryo-EM data of the m31A7-bound S protein were deposited in EMD under EMD-32832 and in the Protein Data Bank (PDB) under ID 7WUH; Crystal structure of

the m31A7-bound RBD was deposited in PDB under ID 7WUE. CAG-hACE2 mice are available from Y.P.H., and C.Y.T. lab under a material transfer agreement (MTA) with the Jackson Laboratory and Academia Sinica, Taiwan. S_{MG} protein and m31A7 antibody are available upon request with Academia Sinica for evaluation by Yi-Li Chou (qbowchou@gate.sinica.edu.tw) and completion of a MTA. This work is licensed under a Creative Commons Attribution 4.0 International (CC BY 4.0) license, which permits unrestricted use, distribution, and reproduction in any medium, provided the original work is properly cited. To view a copy of this license, visit https://creativecommons.org/licenses/by/4.0/. This license does not apply to figures/photos/artwork or other content included in the article that is credited to a third party; obtain authorization from the rights holder before using this material.

Submitted 25 August 2021
Accepted 22 February 2022
Published First Release 1 March 2022
10.1126/scitranslmed.abm0899

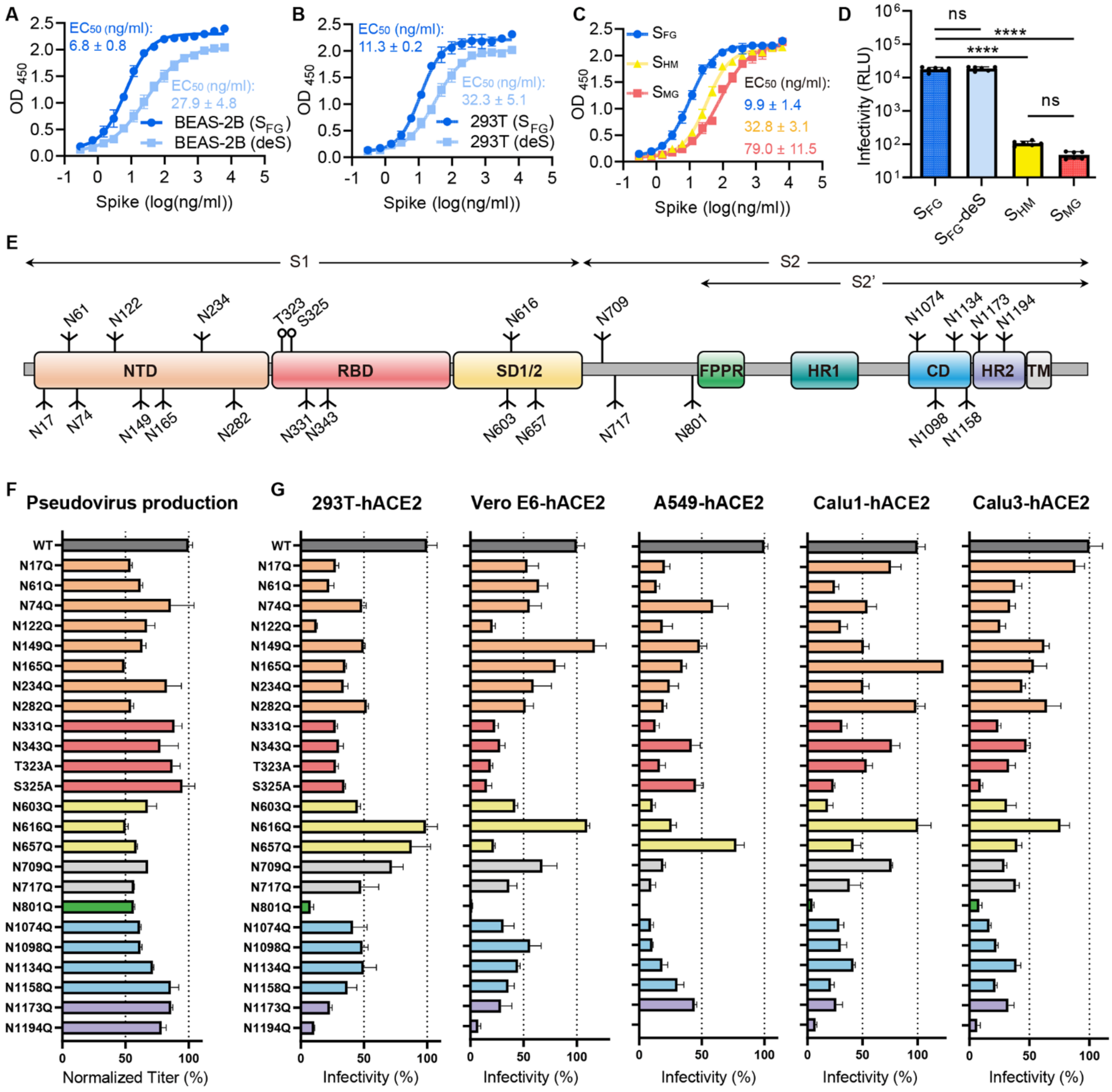
A
B
C
D
E
F
G
EC50 (ng/ml): 6.8 ± 0.8
EC50 (ng/ml): 27.9 ± 4.8
BEAS-2B (SFG)
BEAS-2B (deS)
EC50 (ng/ml): 11.3 ± 0.2
EC50 (ng/ml): 32.3 ± 5.1
293T (SFG)
293T (deS)
SFG
SHM
SMG
EC50 (ng/ml):
9.9 ± 1.4
32.8 ± 3.1
79.0 ± 11.5
OD 450
Spike (log(ng/ml))
Infectivity (RLU)
ns
****
SFG-deS
S1
S2
S2'
NTD
RBD
SD1/2
FPPR
HR1
CD
HR2
TM
Pseudovirus production
Normalized Titer (%)
293T-hACE2
Vero E6-hACE2
A549-hACE2
Calu1-hACE2
Calu3-hACE2
Infectivity (%)

Fig. 1. S protein glycosylation impacts ACE2 receptor binding and SARS-CoV-2 infection. (A to C) Binding avidity of ACE2 was measured for differently glycosylated S protein ectodomains (S_{FG}, original fully glycosylated, blue; deS, non-sialylated, light blue; $S_{HM,}$ high-mannose, yellow; S_{MG}, Mono-GlcNAc, red) from BEAS-2B (A), HEK293T (B) and HEK293S (GnTI$^-$) cells without or with Endo H digestion (C). Data of three technical replicates are shown as mean ± SD and curves fit by nonlinear regression for EC_{50} values. **(D)** Viral infectivity was measured for pseudoviruses carrying differently glycosylated S protein with the same input amount (0.3 μg/mL p24-equivalent) colored accordingly as in (C). RLU: relative luminescence unit. Data of six technical replicates shown as mean ± SD and analyzed with ordinary one-way ANOVA test followed by Tukey's multiple comparisons test. ns, not significant; ****P < 0.0001. **(E)** A schematic view of SARS-CoV-2 S protein (wild type, WT) is shown colored by domain, including N-terminal domain (NTD, 14-306, orange), receptor binding domain (RBD, 319-541, red), two subdomains (SD1/2, 542-685, yellow), fusion peptide proximal region (FPPR, 816-856, green), heptad repeat 1 (HR1, 912-984, teal), connecting domain (CD, 1063-1162, blue), heptad repeat 2 (HR2, 1163-1211, purple) and transmembrane domain (TM, 1214-1234, white). N-glycan (drawn as branches) and *O*-glycan (circles) sites are marked with residue number. S1 and S2 domains are shown above. **(F)** Viral titers are shown for pseudoviruses carrying WT S protein or mutants with glycans removed at each shown glycosite, normalized by p24 quantification, colored accordingly as in (E). **(G)** Infectivity of the same panel of pseudoviruses as in (F) tested in five hACE2-expressing cell lines. Values in (F) and (G) are normalized against WT values (defined as 100%, colored in dark gray) with mean ± SD of three independent experiments.

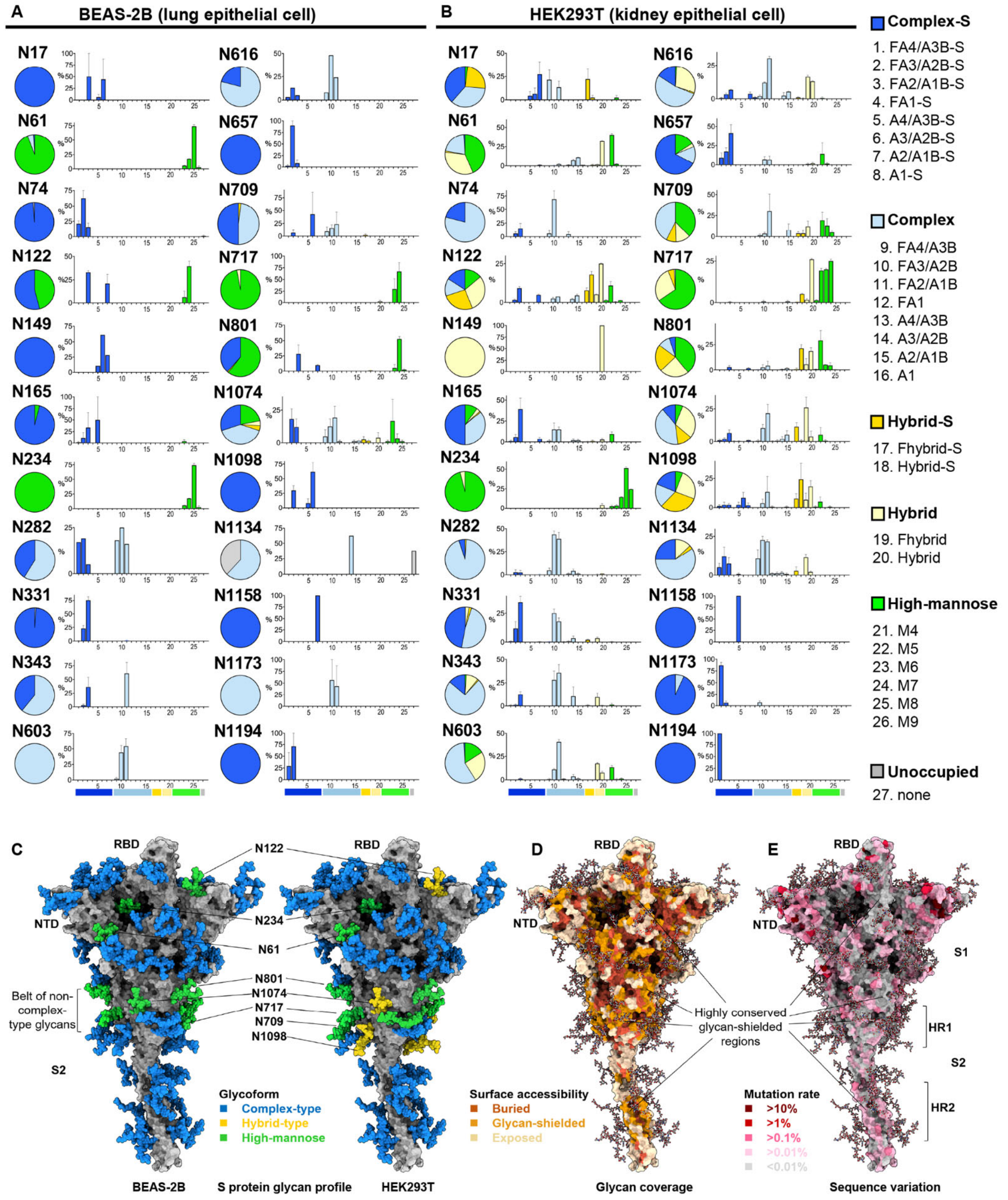
A
BEAS-2B (lung epithelial cell)
B
HEK293T (kidney epithelial cell)
N17
N61
N74
N122
N149
N165
N234
N282
N331
N343
N603
N616
N657
N709
N717
N801
N1074
N1098
N1134
N1158
N1173
N1194
Complex-S
1. FA4/A3B-S
2. FA3/A2B-S
3. FA2/A1B-S
4. FA1-S
5. A4/A3B-S
6. A3/A2B-S
7. A2/A1B-S
8. A1-S
Complex
9. FA4/A3B
10. FA3/A2B
11. FA2/A1B
12. FA1
13. A4/A3B
14. A3/A2B
15. A2/A1B
16. A1
Hybrid-S
17. Fhybrid-S
18. Hybrid-S
Hybrid
19. Fhybrid
20. Hybrid
High-mannose
21. M4
22. M5
23. M6
24. M7
25. M8
26. M9
Unoccupied
27. none
C
D
E
RBD
NTD
S1
S2
HR1
HR2
N122
N234
N61
N801
N1074
N717
N709
N1098
Belt of non-complex-type glycans
Glycoform
Complex-type
Hybrid-type
High-mannose
Surface accessibility
Buried
Glycan-shielded
Exposed
Highly conserved glycan-shielded regions
Mutation rate
>10%
>1%
>0.1%
>0.01%
<0.01%
BEAS-2B
S protein glycan profile
HEK293T
Glycan coverage
Sequence variation

Fig. 2. S protein glycan profiles demonstrate differences in two cell lines and correlate with sequence conservation. **(A and B)** A comparison of the N-glycosylation profile of recombinant S protein expressed from BEAS-2B lung epithelial cells (A) and HEK293T kidney epithelial cells (B) is shown. Glycans are grouped and colored accordingly: complex-S (sialylated complex-type, dark blue), complex (non-sialylated complex-type, light blue), hybrid-S (sialylated hybrid-type, dark yellow) and hybrid (non-sialylated hybrid type, light yellow), high-mannose (green) and unoccupied (gray). The percentage of each group is shown for each glycosite in pie chart and the proportion of each glycoform (No. 1 to 27) in bar chart. The bar graphs represent the mean ± SD of three biological replicates. Detailed structure and percentage of each glycoform can be found in table S1 to S3 and fig. S4 to S6. Fhybrid indicates fucosylated hybrid type glycans. **(C)** Glycan profiles from (A and B) were mapped on the 3D structure of S ectodomain (modeled from 6VSB). Glycans are colored by the highest-abundance group for BEAS-2B (left) or HEK293T (right) data as labeled (complex-type, blue; hybrid-type, yellow; high-mannose, green). Non-complex-type N-glycosites are labeled with residue number. **(D)** Mapping of relative surface accessibility (RSA) on modeled S structure protein is shown, with buried residues colored in dark yellow, glycan-shielded in yellow, and exposed in light yellow. **(E)** Mapping of sequence variation on modeled S protein structure is shown, colored in a heat map with darker red indicating higher mutation rates. Several highly conserved glycan-shielded regions are highlighted. More details for (D and E) can be found in fig. S9 and data file S1.

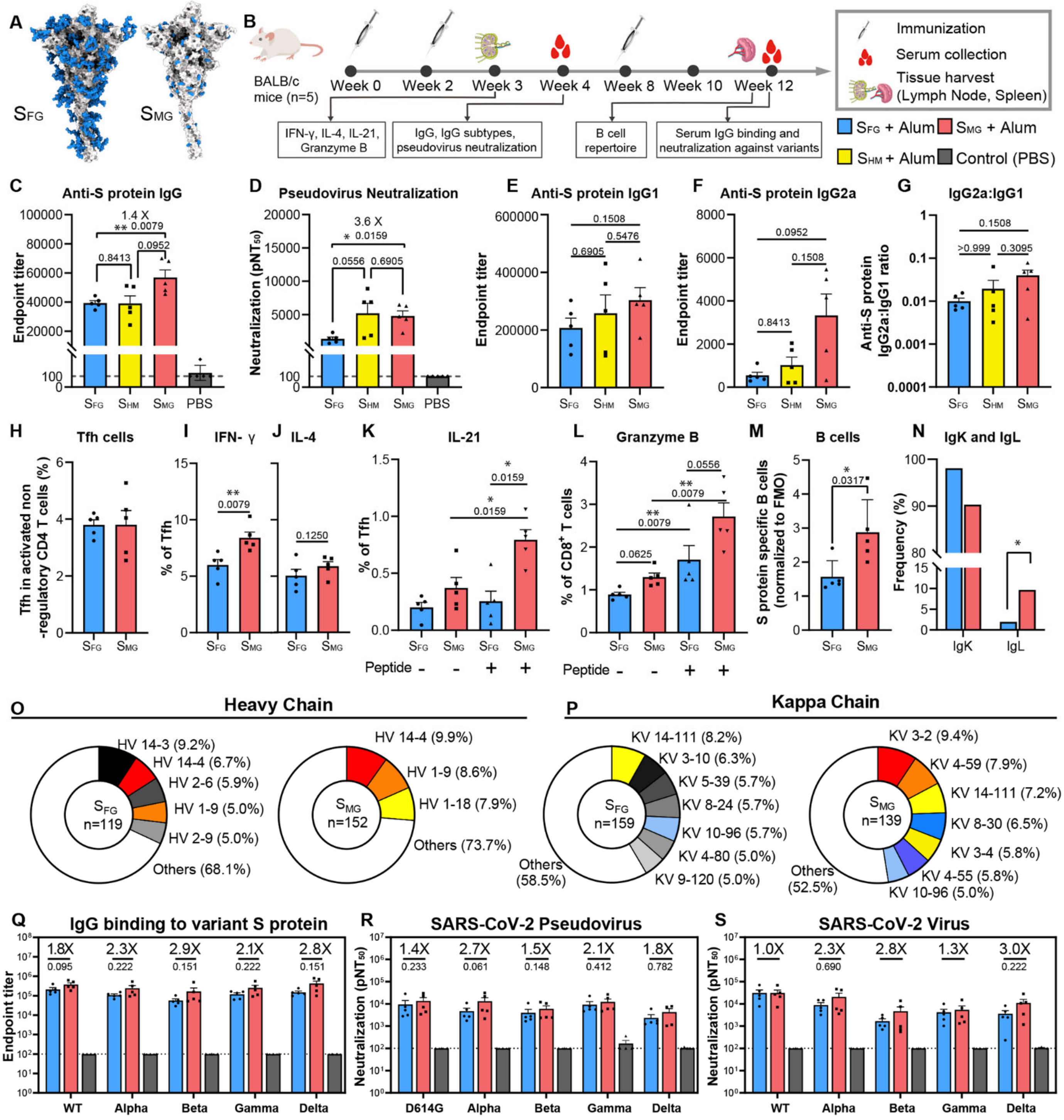

A
SFG
SMG
B
BALB/c mice (n=5)
Week 0
Week 2
Week 3
Week 4
Week 8
Week 10
Week 12
IFN-γ, IL-4, IL-21, Granzyme B
IgG, IgG subtypes, pseudovirus neutralization
B cell repertoire
Serum IgG binding and neutralization against variants
Immunization
Serum collection
Tissue harvest (Lymph Node, Spleen)
SFG + Alum
SMG + Alum
SHM + Alum
Control (PBS)
C Anti-S protein IgG
D Pseudovirus Neutralization
E Anti-S protein IgG1
F Anti-S protein IgG2a
G IgG2a:IgG1
H Tfh cells
I IFN- γ
J IL-4
K IL-21
L Granzyme B
M B cells
N IgK and IgL
O Heavy Chain
P Kappa Chain
Q IgG binding to variant S protein
R SARS-CoV-2 Pseudovirus
S SARS-CoV-2 Virus

Fig. 3. S_{MG} vaccination elicits stronger humoral and cellular immune responses than S_{FG} in BALB/c mice. (A) Structural models of S_{FG} and S_{MG} protein vaccine are shown (according to Fig. 2C). Blue: glycans, Gray: protein. S_{FG} was expressed by HEK293E without further modification. S_{MG} was obtained by enzymatic digestion to truncate all N-glycans of S_{HM} expressed by HEK293S GnTI^{-} to single GlcNAc, whereas *O*-glycans were unmodified. **(B)** Immunization schedule using proteins as in (A) as immunogens in BALB/c mice (n = 5 in each experiment). S_{FG} (blue), S_{HM} (yellow), S_{MG} (red) and control (gray). Alum: Aluminum hydroxide. **(C)** Anti-S protein IgG titers of serum samples were analyzed by ELISA. **(D)** Neutralization titers of serum samples were measured using pseudovirus with WT S protein. **(E to G)** IgG subtype analysis of sera, including IgG1 (E), IgG2a (F) and the IgG2a:IgG1 ratio (G). **(H to K)** The percentage of Tfh in activated non-regulatory CD4 T cells (H) and the percentages of IFN-γ (I), IL-4 (J) and IL-21 (K) expressing Tfh cells ($CD4^{+}CD19^{-}CD44^{hi}Foxp3^{-}PD\text{-}1^{+}CXCR5^{+}$) in lymph nodes (LN) of BALB/c mice by flow cytometry. **(L)** The percentage of granzyme B-producing $CD8^{+}$ T cells ($CD3^{+}$ $B220^{-}CD8^{+}$ $CD49b^{-}$) in the lymph node (LN) of BALB/c mice analyzed by flow cytometry. **(M)** The ratio of S protein-specific B cells ($CD3^{-}$ $CD19^{+}$ S protein^{+}) (%) normalized to Fluorescence Minus One (FMO) control staining (stained without S protein) (%) in the spleen is shown. **(N)** Kappa and lambda light chain usage is shown. **(O and P)** Heavy (O) and kappa (P) chain distribution of B cell repertoire analysis. Less than 5% usage is shown in white. **(Q to S)** Anti-S protein IgG titers (Q), pseudovirus neutralization titers (R) and authentic virus neutralization titers (S) are shown for serum isolated from BALB/c mice after 3 doses of indicated vaccines against SARS-CoV-2 WT (or D614G) and variants (number above each bar indicate fold of increase of S_{MG} comparing to S_{FG} group). pNT_{50} represents the reciprocal dilution achieving 50% neutralization. The dotted line in bar charts represents the lower limit of detection. Data are shown as mean ± SEM and analyzed by two-sided Mann–Whitney *U*-test to compare two experimental groups, except in (N), where 5 samples were pooled together and a chi-squared test was used. *P* values shown above each bar. $^{*}P < 0.05$; $^{**}P < 0.01$.

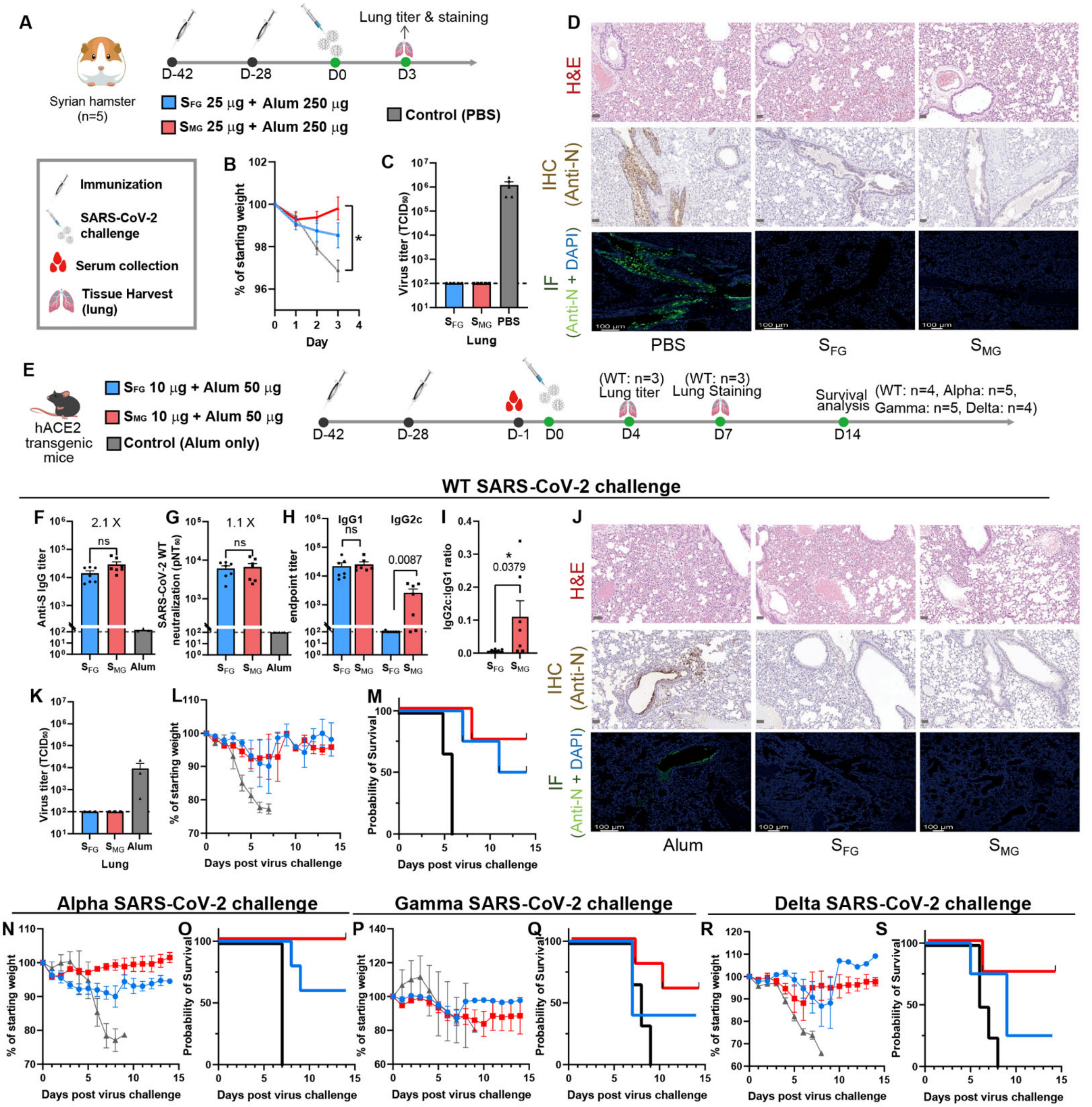
A
Lung titer & staining
D-42
D-28
D0
D3
Syrian hamster
(n=5)
SFG 25 μg + Alum 250 μg
SMG 25 μg + Alum 250 μg
Control (PBS)
Immunization
SARS-CoV-2 challenge
Serum collection
Tissue Harvest (lung)
B
% of starting weight
Day
C
Virus titer (TCID50)
SFG SMG PBS
Lung
D
H&E
IHC (Anti-N)
IF (Anti-N + DAPI)
PBS
SFG
SMG
E
hACE2 transgenic mice
SFG 10 μg + Alum 50 μg
SMG 10 μg + Alum 50 μg
Control (Alum only)
D-42
D-28
D-1
D0
(WT: n=3) Lung titer
D4
(WT: n=3) Lung Staining
D7
Survival analysis
(WT: n=4, Alpha: n=5, Gamma: n=5, Delta: n=4)
D14
WT SARS-CoV-2 challenge
F
2.1 X
ns
Anti-S IgG titer
SFG SMG Alum
G
1.1 X
ns
SARS-CoV-2 WT neutralization (pNT50)
SFG SMG Alum
H
IgG1
IgG2c
ns
0.0087
endpoint titer
SFG SMG SFG SMG
I
0.0379
IgG2c:IgG1 ratio
SFG SMG
J
H&E
IHC (Anti-N)
IF (Anti-N + DAPI)
Alum
SFG
SMG
K
Virus titer (TCID50)
SFG SMG Alum
Lung
L
% of starting weight
Days post virus challenge
M
Probability of Survival
Days post virus challenge
Alpha SARS-CoV-2 challenge
N
% of starting weight
Days post virus challenge
O
Probability of Survival
Days post virus challenge
Gamma SARS-CoV-2 challenge
P
% of starting weight
Days post virus challenge
Q
Probability of Survival
Days post virus challenge
Delta SARS-CoV-2 challenge
R
% of starting weight
Days post virus challenge
S
Probability of Survival
Days post virus challenge

Fig. 4. S_{MG} vaccination provides enhanced protection against SARS-CoV-2 infection in vivo. (A) The immunization schedule for Syrian hamsters is shown. S_{FG} (blue), S_{MG} (red) and control (gray). Alum: Aluminum hydroxide. **(B)** Weight change was measured in Syrian hamsters after WT SARS-CoV-2 challenge. **(C)** Lung virus titers of challenged hamsters are shown. The dashed line indicates the lower limit of detection. **(D)** Representative images shown histopathology, immunohistochemistry and immunofluorescence of lungs from an infected hamster (3 dpi). First row: H&E staining, scale bar: 50 μm. Second row: immunohistochemistry (IHC) staining, scale bar: 50 μm. Third row: immunofluorescence (IF) staining, scale bar: 100 μm. SARS-CoV-2 N-specific polyclonal antibodies were used for virus detection as brown dots in IHC and green dots in IF staining. Blue: 4,6-diamidino-2-phenylindole (DAPI). **(E)** The immunization schedule for CAG-hACE2 or K18-hACE2 transgenic mice is shown. **(F to I)** Anti-S IgG titers (F), SARS-CoV-2 WT microneutralization titers (G) and subtype IgG analysis, including IgG1, IgG2c (H) and IgG2c:IgG1 ratio (I) are shown for serum samples collected from immunized CAG-hACE2 transgenic mice (n=7). **(J)** Representative histopathology, immunohistochemistry and immunofluorescence of the infected mouse lungs (7 dpi) are shown. Scale bars the same as in (D). **(K)** Lung virus titers of the infected CAG-hACE2 mice (n=3). The dashed line indicates the lower limit of detection. **(L and M)** Weight change (L) and survival analysis (M) are shown for WT-SARS-CoV-2-challenged CAG-hACE2 transgenic mice (n=4). **(N and O)** Weight change (N) and survival analysis (O) are shown for SARS-CoV-2 alpha variant-challenged CAG-hACE2 transgenic mice (n=5). **(P and Q)** Weight change (P) and survival analysis (Q) are shown for SARS-CoV-2 gamma variant-challenged CAG-hACE2 transgenic mice (n=5). **(R and S)** Weight change (R) and survival analysis (S) are shown for SARS-CoV-2 delta variant-challenged K18-hACE2 transgenic mice (n=4). Data shown as mean ± SEM and analyzed by two-sided Mann–Whitney *U*-tests to compare two experimental groups. ns, not significant $^*P < 0.05$; $^{**}P < 0.01$.

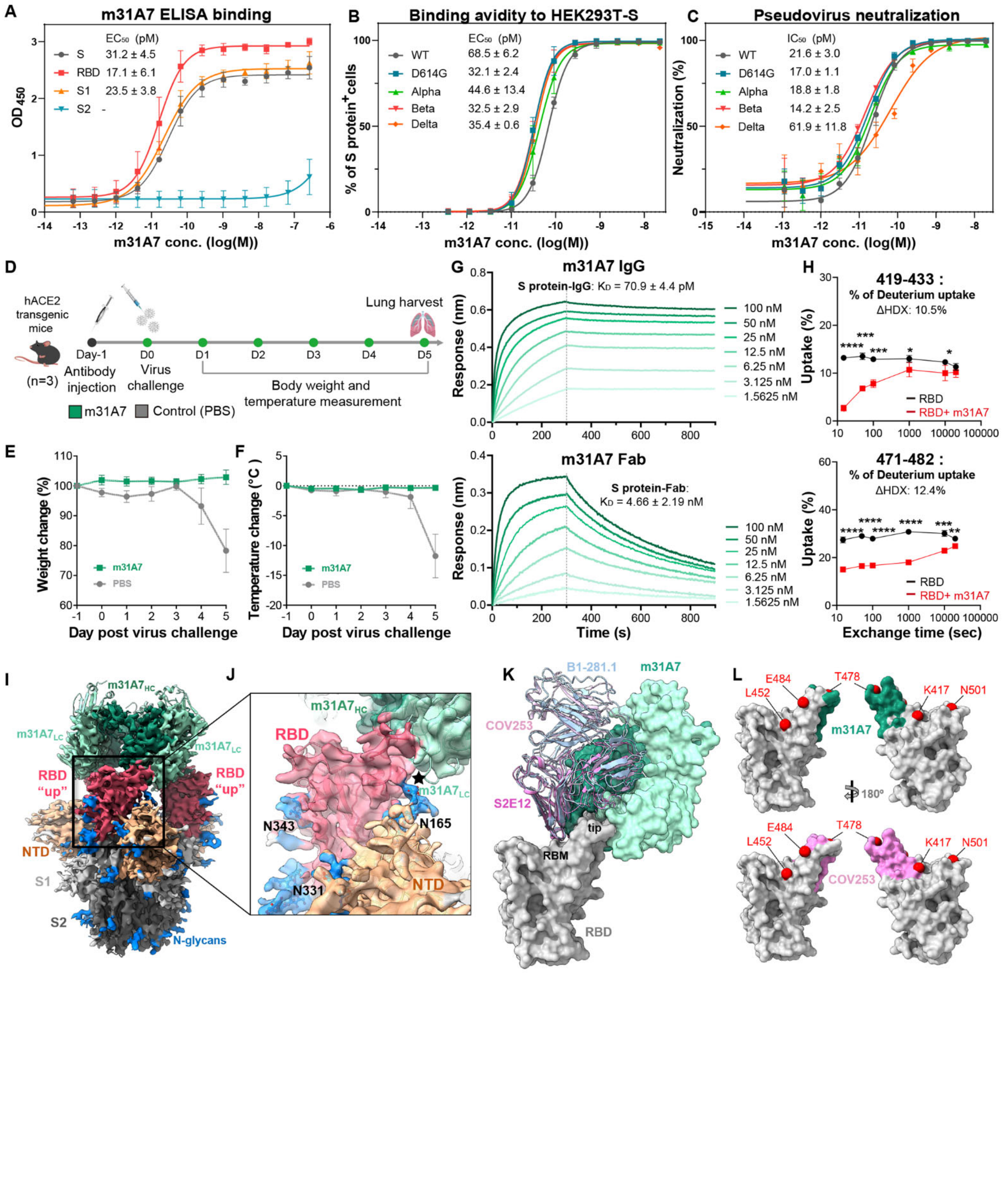

A
m31A7 ELISA binding
EC50 (pM)
S 31.2 ± 4.5
RBD 17.1 ± 6.1
S1 23.5 ± 3.8
S2 -
OD 450
m31A7 conc. (log(M))
B
Binding avidity to HEK293T-S
EC50 (pM)
WT 68.5 ± 6.2
D614G 32.1 ± 2.4
Alpha 44.6 ± 13.4
Beta 32.5 ± 2.9
Delta 35.4 ± 0.6
% of S protein+cells
m31A7 conc. (log(M))
C
Pseudovirus neutralization
IC50 (pM)
WT 21.6 ± 3.0
D614G 17.0 ± 1.1
Alpha 18.8 ± 1.8
Beta 14.2 ± 2.5
Delta 61.9 ± 11.8
Neutralization (%)
m31A7 conc. (log(M))
D
hACE2 transgenic mice
(n=3)
Day-1 Antibody injection
D0 Virus challenge
D1 D2 D3 D4 D5
Lung harvest
Body weight and temperature measurement
m31A7 Control (PBS)
E
Weight change (%)
m31A7
PBS
Day post virus challenge
F
Temperature change (°C)
m31A7
PBS
Day post virus challenge
G
m31A7 IgG
S protein-IgG: KD = 70.9 ± 4.4 pM
100 nM
50 nM
25 nM
12.5 nM
6.25 nM
3.125 nM
1.5625 nM
Response (nm)
m31A7 Fab
S protein-Fab: KD = 4.66 ± 2.19 nM
Time (s)
H
419-433 :
% of Deuterium uptake
ΔHDX: 10.5%
RBD
RBD+ m31A7
Uptake (%)
471-482 :
% of Deuterium uptake
ΔHDX: 12.4%
Exchange time (sec)
I
m31A7HC
m31A7LC
RBD "up"
NTD
S1
S2
N-glycans
J
m31A7HC
RBD
m31A7LC
N343
N165
N331
NTD
K
B1-281.1
m31A7
COV253
S2E12
tip
RBM
RBD
L
E484
T478
L452
K417
N501
m31A7
180°
COV253

Fig. 5. Functional, prophylactic, and structural characterization of antibody m31A7 elicited by S_{MG} vaccination indicates cross-neutralizing capacity. (A) ELISA binding of m31A7 to S1, S2, RBD or the entire S ectodomain. **(B)** Flow cytometry analysis of m31A7 binding to HEK293T cells expressing S protein of SARS-CoV-2 WT and variants. **(C)** Neutralization activity of m31A7 against pseudoviruses carrying WT or variant S proteins. Data of three technical replicates shown for (A), (B) and (C) as mean ± SD and curves fit by nonlinear regression for EC_{50} values. **(D)** Antibody injection and challenge schedule for K18-hACE2 transgenic mice (n=3) is shown. **(E and F)** Weight change (E) and body temperature change (F) are shown for mice treated with m31A7 or PBS. Data are presented as mean ± SEM. **(G)** Binding kinetics of m31A7 IgG and Fab to S protein are presented, with dissociation constants (K_D) shown above. **(H)** Epitope mapping by HDX-MS of m31A7 is shown in a time course revealing two peptide candidates, 419-433 and 471-482, with greater than 10% ΔHDX at 15 s. Data shown as mean ± SD and analyzed by multiple *t* tests at each time point. **P* < 0.05; ***P* <0.01; ****P* < 0.001; *****P* < 0.0001. **(I)** The Cryo-EM map fitted with m31A7-Fab/S protein complex structure is shown. Heavy-chain: dark green; light-chain: light green; RBD: red, NTD: orange; the rest of S1: light gray; S2: dark gray; N-glycans: blue. **(J)** An enlarged view of RBD-m31A7 interface is shown. The star marks the vicinity between m31A7 light chain and N165-glycan. **(K)** Superimposition of previously reported mAbs S2E12 (magenta), COV253 (pink) and B1-182.1 (light blue) (PDB 7BEN, 7K4N and 7MLZ) onto the m31A7-bound RBD (gray). The receptor binding motif (RBM) and RBD tip are highlighted. **(L)** A footprint comparison of COV253 (pink) and m31A7 (green) on RBD (gray) shows similarity, with residues of VOCs labeled and drawn as red spheres.
