# Supplementary material for: Vaccination with SARS-CoV-2 spike protein lacking glycan shields elicits enhanced protective responses in animal models
Source: Sci Transl Med. 2022 Apr 6;14(639):eabm0899. doi: 10.1126/scitranslmed.abm0899 (PMC9802656; doi:10.1126/scitranslmed.abm0899)
Supplement: Supplementary file 2 — Materials and Methods Figs. S1 to S16 Tables S1 to S3 References (48–52) [file scitranslmed.abm0899_sm.pdf]

Science Translational Medicine
AAAS

Supplementary Materials for

Vaccination with SARS-CoV-2 spike protein lacking glycan shields elicits enhanced protective responses in animal models

Han-Yi Huang *et al.*

Corresponding author: Che Ma, cma@gate.sinica.edu.tw; Kuo-I Lin, kuoilin@gate.sinica.edu.tw; Chi-Huey Wong, chwong@gate.sinica.edu.tw

Sci. Transl. Med. **14**, eabm0899 (2022)
DOI: 10.1126/scitranslmed.abm0899

The PDF file includes:

Materials and Methods
Figs. S1 to S16
Tables S1 to S3
References (*48*–*52*)

Other Supplementary Material for this manuscript includes the following:

Data file S1
MDAR reproducibility checklist

Supplementary Materials and Methods

Cell lines and culture medium

For virus infection studies, HEK 293T (American Type Culture Collection (ATCC) CRL-3216), HEK293T-ACE2 cells, Vero E6 (ATCC CRL-1586), Vero E6 cells expressing angiotensin converting enzyme 2 (Vero E6-ACE2) cells, A549 (ATCC CCL-185) and A549-ACE2 cells were cultured in Dulbecco's modified Eagle medium (DMEM) (Gibco). Calu1 (ATCC HTB-54) and Calu1-ACE2 cells were cultured in DMEM/F-12 (Gibco). Calu3 (ATCC HTB-55) and Calu3-ACE2 cells were cultured in Minimum Essential Medium (Gibco). The stably expressing hACE2 cell lines including HEK293T-ACE2, Vero E6-ACE2, A549-ACE2, Calu3-ACE2 Calu1-ACE2 were provided by H.C.H Laboratory, IBMS, Academia Sinica. All the cells above were cultured in medium supplemented with 10% fetal bovine serum (FBS) (Gibco) and 1% Penicillin-Streptomycin solution (Gibco). For spike (S) protein expression, BEAS-2B cells (ATCC CRL-9609) or HEK 293T (ATCC CRL-3216) were cultured in RPMI-1640 (Gibco) or DMEM (Gibco) with 10% FBS (Gibco) and 1% Penicillin-Streptomycin solution (Gibco). HEK293 EBNA (ATCC CRL-10852) and HEK293S GnTI^{-} (ATCC CRL-3022) cells were cultured in Freestyle 293 expression medium (Invitrogen) supplemented with 0.5% FBS (Gibco). For antibody production, Expi293F cells were purchased and maintained in Expi293 Expression and LV-MAX production (Thermo Fisher Scientific).

SARS-CoV-2 S protein production and characterization

For expression in HEK 293T cells (ATCC CRL-3216) or BEAS-2B cells (*Homo sapiens*, lung) cells (ATCC CRL-9609), SARS-CoV-2 (Wuhan/WH01/2019 strain) S protein ectodomain (1-1208) was codon-optimized for *Homo sapiens* with a T4 fibritin foldon trimerization motif (*35*), residues 986 and 987 substituted by proline, and sequence 682 to 685 replaced with "GSAS" to

abolish the furin cleavage site (S2P) (*36*). This was followed by addition of a 6xHisTag at the C-terminus and cloning into the mammalian expression vector pcDNA3.1 (*36*). This expression vector was transiently transfected into HEK293T or BEAS-2B using X-tremeGENE HP DNA Transfection Reagent (Roche), and cells were incubated for 72 hours at 37 ºC. For large-scale expression in suspension cells for vaccine studies, SARS-CoV-2 (WH01) S protein sequence (14 to 1209) was codon-optimized for *Homo sapiens* with the furin cleavage site replaced by GSAG and 2P substitution (*36*). The N-terminal signal peptide is from Hemagglutinin (A/Brisbane/59/2007) and the C-terminus contains a thrombin cleavage site, a T4 fibritin foldon, and 6xHisTag. The construct was cloned into pTT vector, and the plasmid was mixed with polyethyleneimine (PEI) and transfected into HEK293 EBNA (ATCC CRL-10852) or HEK293S GnTI^{-} (ATCC CRL-3022) cells followed by incubation at 32°C for 5 to 6 days with continuous shaking. S proteins were purified from supernatants using Ni-NTA affinity column (GE Healthcare) and concentrated by Amicon Ultrafiltration Unit (MW100K cutoff, Millipore) in phosphate buffered saline (PBS), pH 7.4, or Tris-based buffer (20 mM Tris/HCl, pH 8.0, 150 mM NaCl) before further purification by Superose 6 Increase 10/300 GL (GE Healthcare). The purified S_{HM} expressed by HEK293S GnTI^{-} cells was further treated with Endo H (New England Biolabs) overnight at 25°C in a ratio of 50:1 (w/w) to produce S_{MG}. For Endo H removal, S_{MG} was either buffer-exchanged three times (MW 100 kDa cutoff) or repurified by Enrich SEC 650 (10 × 300 column, Bio-Rad) in buffer containing 20 mM Tris pH 8.0, 20 mM NaCl, 50 mM L-Arginine and 50 mM L-Glutamate. The purity was monitored by using 10% SDS-PAGE stained by Coomassie Brilliant Blue-Plus (EBL Biotechnology) and the proteins were confirmed using Western blot with anti- $(his)_6$ antibodies (Qiagen) or polyclonal anti-SARS-CoV-2 S protein antibodies (LKT laboratories) and horseradish peroxidase (HRP)-

conjugated secondary antibodies (Perkin Elmer). To further characterize S protein, the binding ability to hACE2 was evaluated by enzyme-linked immunosorbent assay (ELISA), the glycosylation profiles of S protein were analyzed by mass spectrometry (glycoform categorization followed previous studies (*2*)), and the structures of S_{FG} and S_{MG} were determined by negative staining.

ELISA determination of SARS-CoV-2 S protein and human ACE2 binding.

ELISA (96-well) plates were coated with 0.2 μg per well hACE2 (ACROBiosystems) protein in 100 mM sodium bicarbonate pH 8.8 at 4 °C for overnight, blocked with PBST (1×PBS, 0.1% Tween 20, pH 7.4) containing 1% bovine serum albumin (BSA) and 5% (w/v) skim milk at 37 °C for 1 hour, and washed 3 times with PBST before being incubated with 100 μl of SARS-CoV-2 S protein in 2-fold serial dilutions at 37 ºC for 1 hour. After three washes with PBST, the plates were incubated with anti-SARS-CoV-2 S polyclonal antibody (1:2000) (provided by C.M. Lab) at 37 °C for 1 hour, followed by three washes with PBST and then incubation with 200 μl of HRP-conjugated goat anti-rabbit IgG (1:5000) (Jackson Immuno-Research) for 1 hour at 37 ºC. The plates were again washed 3 times with PBST and developed with 50 μl of the 1-Step Ultra 3,3',5,5'-Tetramethylbenzidine (TMB) substrate (Thermo Fisher Scientific) for 4 min at 25°C. The reaction was stopped with 50 μl of 1 M H_2SO_4. The absorbance of each well was measured at 450 nm using a SpectraMax M5 (Molecular Devices).

N-Glycosylation profile on SARS-CoV-2 S protein by mass spectrometry

SARS-CoV-2 S protein (20 μg) was incubated in 50 mM triethylammonium bicarbonate (TCEP) (Sigma-Aldrich) at 55°C for 1 hour. The reduced S protein was alkylated by adding 18 mM iodoacetamide (IAA) and incubated for 30 min in the dark at room temperature. Protein samples

were divided in five groups to be digested separately using different proteases and combinations: trypsin (Promega, 1:100 w/w) alone, chymotrypsin (Promega, 1:50 w/w) alone, α-lytic protease (New England Biolabs, 1:100 w/w) alone, trypsin plus chymotrypsin, or trypsin plus α-lytic protease, for overnight incubation at 37°C before they were acidified and processed for LC-MS/MS (liquid chromatography with tandem mass spectrometry) analysis. Samples were detected by LC-ESI-MS on a Orbitrap Fusion mass spectrometer (Thermo Fisher Scientific) equipped with EASY-nLC 1200 system (Thermo Fisher Scientific) and EASY-spray source (Thermo Fisher Scientific). The digestion solution was injected (5μl) at 1 μl/min flow rate on to easy column (C18, 0.075 mm X 150 mm, ID 3 μm; Thermo Fisher Scientific). Chromatographic separation used 0.1% formic acid in water as mobile phase A and 0.1% formic acid in 80% acetonitrile as mobile phase B operated at 300 nl/min flow rate. Briefly, the gradient employed was 5% buffer B at 2 min to 60% buffer B at 55 min. Full-scan mass spectrometry condition: mass range m/z 375-1800 (AGC target 5E5) with lock mass, resolution 60,000 at m/z 200, and maximum injection time of 50 ms. The MS/MS was run in top speed mode with 3s cycles with CID and HCD; the dynamic exclusion duration was set to 60s with a 10 ppm tolerance around the selected precursor and its isotopes. Electrospray voltage was maintained at 1.8 kV and capillary temperature was set at 275°C. For glycopeptide identification, Byonic software 4.2.10 (protein metric) was used for the identification of summary formulas of glycans associated with glycopeptides (glycan database, 132 entries; precursor mass tolerance, 10 ppm; fragment mass tolerance, 0.1 Da; maximum missed cleavages, 5; and cysteine carbamidomethylation and methionine oxidation). Glycoform categorization followed previous studies (*2*) and charts were generated using GraphPad Prism 9.0.0.

Negative staining of purified S_{FG} or S_{MG} SARS-CoV-2 S protein

Freshly purified S_{FG} or S_{MG} protein was diluted to 20 μg/ml in 20 mM Tris/HCl, pH 8.0, 150 mM NaCl before being applied onto glow-discharged carbon-coated 400 mesh CF400-CU grids (Electron Microscopy Sciences) for 1 min at room temperature. Excessive protein solutions were removed with filter paper and grids were washed 3 times in filtered pure water prior to staining by 1% uranyl acetate for 60 seconds at room temperature. Data was collected using a FEI Tecnai G2 F20 S-TWIN electron microscope (Thermo Fisher Scientific) operating at 120 keV and a magnification of 29K that resulted in a pixel size of 3.13 Å at the specimen plane. Particle selection, 2D classification, and 3D reconstruction were processed by cisTEM (*48*). The S protein modeled structure (without HR2 and TM) was used for fitting in map in ChimeraX (*46*) and volumes were drawn at level 3.08.

Expression analysis of S protein N-glycosite mutants

Choices of amino acid substitutions were based on S protein variation statistics from Global initiative on sharing all influenza data (GISAID) (*9*), using the naturally occurring mutations with highest abundance at either N or S/T site for each N-glycosite and excluding those strictly conserved sites with variation below 2 cases. S protein construction, mutagenesis, and sequencing were carried out as described above. The expression of S protein mutants was evaluated in HEK293E suspension cells, with the supernatant run on a 7.5% SDS-PAGE analyzed by western blot using HRP-conjugated anti-(his_6) monoclonal antibody (provided by C.M. lab). The original S2P construct (WT strain) was used as the positive control for every batch. Reduction of protein expression could be the collective result of multiple factors including reduced protein solubility, loss of protein integrity (misfolding), and disrupted protein secretion. Severe reduction of expression was defined as signals below 30% of the WT signal.

Pseudovirus production for neutralization assay and infectivity of glycan-engineered virus

For production and purification of SARS-CoV-2 pseudotyped lentivirus, the pseudotyped lentivirus carrying SARS-CoV-2 S protein was generated by transiently transfecting HEK-293T cells with pCMV-ΔR8.91, pLAS2w.Fluc.Ppuro and pcDNA3.1-nCoV-SΔ18 (or pcDNA3.1-nCoV-SΔ18 D614G). HEK-293T cells were seeded one day before transfection, and indicated plasmids were delivered into cells by using TransITR-LT1 transfection reagent (Mirus). The culture medium was refreshed at 16 hours and harvested at 48 hours and 72 hours post-transfection. Cell debris was removed by centrifugation at 4,000 g for 10 min, and the supernatant was passed through a 0.45 μm syringe filter (Pall Corporation). The pseudotyped lentivirus was aliquoted and then stored at -80°C.

To estimate the lentiviral titer by AlarmaBlue assay, the transduction unit (TU) of SARS-CoV-2 pseudotyped lentivirus was estimated by using cell viability assay in response to the limited dilution of lentivirus. In brief, HEK-293T cells stably expressing human ACE2 were plated on 96-well plate one day before lentivirus transduction. To determine the titer of pseudotyped lentivirus, different amounts of lentivirus were added into the culture medium containing polybrene (final concentration 8 μg/ml). Spin infection was carried out at 1,100 g in a 96-well plate for 30 min at 37°C. After incubating cells at 37°C for 16 hours, the culture medium containing the virus and polybrene was removed and replaced with fresh complete DMEM containing 2.5 μg/ml puromycin. After treating puromycin for 48 hours, the culture medium was removed, and the cell viability was detected by using 10% AlarmaBlue reagents according to the manufacturer's instruction. The survival rate of uninfected cells (without puromycin treatment) was set as 100%. The virus titer (transduction units) was determined by plotting the survival cells versus diluted viral dose.

For production of glycan-engineered pseudoviruses in Fig 1D, the same pseudovirus production system was used, and S_{FG} and S_{HM} were produced in the absence and presence of 15 μM kifunensine (*6*). S_{FG} or S_{HM} pseudovirus was further incubated with 30 μg/ml neuraminidase or 30 μg/ml EndoH overnight at 4 °C respectively to produce S_{FG}-deS and S_{MG} pseudovirus. The PEG-it (SBI) was then used to concentrate and separate the virus from enzymes. The virus S protein glycoform was verified by western blotting with anti-S polyclonal antibody (1:1500, provided by C.M lab) and the viral titer was determined using the lentiviral p24 ELISA kit (Cell Biolabs, Inc.). For analyzing the infectivity, serial diluted lentivirus was added into the pre-seeded HEK293T-hACE2 cells in DMEM with 10% FBS and 10 μg/ml blasticidin in 96-well plates. After incubating cells at 37°C for 16 hours, the culture medium containing virus was removed and replaced with fresh same medium. After 48 hours, the expression of luciferase gene was determined by using the Bright-Glo Luciferase Assay System (Promega). The relative light unit (RLU) was determined by Cytation 5 imaging reader (BioTek).

Glycosite-specific S mutants pseudovirus production and infectivity analysis

Pseudoviruses with deletion of C-terminal (Δ19 or Δ27) SARS-CoV-2 (WH01) S mutants were generated with or without 8 membrane-proximal residues of HIV-1 Env cytoplasmic domain (NRVRQGYS), and with or without arginine substitutions at 812 and 813 (KRRKR) to facilitate efficient pseudovirus formation. Pseudoviruses were cloned in expression vector pVax. Glycosite-specific mutations were generated using QuikChange Lightning Multi Site-Directed Mutagenesis Kit (Agilent Technologies) into the pVax-nCoV-SΔ19 construct, and XL10-Gold ultracompetent cells were used for transformation and plasmids from single colonies were confirmed by sequencing. Each corresponding pseudovirus strain was produced in the same way as above, except using co-transfection of each pVax-S plasmid with the luciferase-expressing

HIV-1 genome plasmid (pNL4-3.luc.RE) according to manufacturer's instructions. The vesicular stomatitis virus-G (VSV-G) protein was used as control to evaluate entry efficiency. Pseudovirus titer was quantified using HIV-1 Gag p24 ELISA (R&D Systems): p24-specific monoclonal antibody was pre-coated on a 96-well plate, blocked and washed as mentioned above, and incubated for 1 hour with target pseudoviruses in 1000, 1500, 2000, 5000 and 10000-fold dilutions, or with p24 standard at 500, 250, 125, 62.5, 31.3, 15.6, 7.81, and 0 pg/ml. Each well was then washed three time in PBST and incubated with p24-specific antibody for 1 hour before washing and reacting with 200μl substrate solution for 7 min at 25 °C. Color development was stopped by adding 50 μl of stop solution and absorbance at 450 nm was measured using SpectraMax M5 (Molecular Devices) with the resulting titer calculated from the standard curve. Pseudovirus infection efficiency was determined as follows: HEK293T cells were seeded on a 96-well plate and inoculated with 100 μl virus-containing medium incubated at 37 °C before medium was refreshed at 24 hours post-infection; after another 24 hours, cells were harvested and lysed with 60 μl of Glo lysis buffer at room temperature for 5 min. The cell lysate (40μl) was mixed with 100 μl of the assay solution from the Luciferase Assay System (Promega) for luciferase activity quantification using a CLARIOstar plate-reader.

Serum antibody titer evaluation

Anti-S protein ELISA was used to determine IgG titer of immunized mouse sera. Plates were coated with 50 ng/well of S protein expressed from HEK293E (S_{FG}) of SARS-CoV-2 WT or variants (S2P) as indicated, and then blocked with 5% skim milk in PBS. Immunized mouse serum samples were applied as primary antibody and anti-mouse HRP-conjugated secondary antibody was added next. Subsequently, 50 μl peroxidase substrate solution (TMB) (SeraCare) was applied followed by adding 50 1M H_2SO_4 after 10 min and absorbance (OD 450 nm) was

measured by CLARIOstar plate-reader. Every step was separated by three washes with PBST. The S protein variants include WT: original S protein; D614G: D614G; B.1.1.7 /(Alpha): 69-70 deletion, 144 deletion, N501Y, A570D, D614G, P681H, T716I, S982A and D1118H; B.1.351/ (Beta): L18F, D80A, D215G, 242-244 deletion, R246I, K417N, E484K, N501Y, D614G and A701V; P.1/ (Gamma): L18F, T20N, P26S, D138Y, R190S, K417T, D427N, E484K, N501Y, D614G, H655Y, T1027I, V1176F; and B.1.617.2 /(Delta): T19R, E156G, 157-158 deletion, L452R, T478K, D614G, P681R, D950N (*9*).

Pseudovirus neutralization assay for immunized mouse sera and m31A7

For pseudovirus neutralization assays, heat-inactivated serum samples or purified antibody was serially diluted accordingly and incubated with 1,000 transduction units (TU) of SARS-CoV-2 (WT or variants) pseudotyped lentivirus in DMEM (supplemented with 1% FBS and 100 U/ml Penicillin/Streptomycin) for 1 hour at 37°C. The mixture was then inoculated with 10,000 HEK293T cells stably expressing human ACE2 in 96-well plates. The culture medium was replaced with fresh complete DMEM (supplemented with 10% FBS and 100 U/ml Penicillin/ Streptomycin) at 16 hours post-infection and cells were continuously cultured for another 48 hours before performing luciferase assay using Bright-Glo Luciferase Assay System (Promega) to measure the relative light unit (RLU) by Tecan i-control (Infinite 500). The percentage of inhibition was calculated as the ratio of RLU reduction in the presence of each diluted sample to the RLU value of no-sample control and the calculation formula was $(RLU^{control} - RLU^{sample}) / RLU^{control}$.

Microneutralization and plaque reduction assays

Heat-inactivated serum and SARS-CoV-2 (included WT and variants) were incubated at 37°C for 1 hour in a 96 well tissue culture plate, then $1.5x10^4$ Vero E6 cells per well were added to the

mixture. The plate was then cultured for 48 hours with 0.5% serum in medium, then washed and fixed with 50% methanol 50% acetone. Anti-N ELISA was then used to determine virus titer. Plates were blocked with 5% skim milk 0.5% BSA, and rabbit anti-N primary antibody (rabbit polyclonal antibody raised against N protein of SARS-CoV-2 (WH01), a gift from An-Suei Yang lab, Academia Sinica), and HRP-conjugated secondary antibody were sequentially added. Peroxidase substrate solution (TMB) and 1M H_2SO_4 stop solution were used and the absorbance (OD 450 nm) was read by CLARIOstar plate-reader. The SARS-CoV-2 included WT SARS-CoV-2 (hCoV-19/Taiwan/4/2020, GISAID accession ID: EPI_ISL_411927), Alpha variant (hCoV-19/Taiwan/792/2020, GISAID accession ID: EPI_ISL_1381386), Beta variant (hCoV-19/Taiwan/1013/202, GISAID accession ID:EPI_ISL_5854267), Gamma variant (hCoV-19/Taiwan/906/2021, GISAID accession ID: EPI_ISL_ 5854262), and Delta SARS-CoV-2 (hCoV-19/Taiwan/1144/2021, GISAID accession ID: EPI_ISL_5854263).

For plaque reduction assay, Vero E6 cells were seeded into 24□well culture plates in DMEM with 10% FBS and antibiotics (1% penicillin/streptomycin)1 day before infection. Serum samples were heated inactivated at 56°C for 30 min. SARS□CoV□2 D614G variant (hCoV-19/Taiwan/NTU03/2020, GISAID accession ID: EPI_ISL_413592) or B.1.1.7 (hCoV-19/Taiwan/NTU49/2020, GISAID accession ID: EPI_ISL_1010728) was incubated with antibodies for 1 hour at 37°C before adding to the cell monolayer for another hour. Subsequently, virus□antibody mixtures were removed, and the cell monolayers were washed once with PBS before covering with media containing 1% methylcellulose for 5 to 7 days. The cells were then fixed with 10% formaldehyde overnight. After removal of the overlay media, the cells were stained with 0.5% crystal violet, and the plaques were counted. The percentage of inhibition was calculated as $[1 - (VD/VC)] \times 100\%$, where VD and VC refer to the virus titers in the presence

and absence of serum, respectively.

Intracellular cytokine staining of Tfh cells

After euthanizing mice 7 days after the last immunization, cells from inguinal and popliteal lymph nodes from each mouse were pooled and re-suspended in RPMI-1640 containing 10% heat-inactivated FBS, 1% Penicillin/Streptomycin, and 50 μM 2-mercaptoethanol (Gibco) at the density of 2×10^6 cells/ml. Cells were stimulated with 50 ng/mL phorbol 12-myristate 13-tacetate (PMA, Sigma-Aldrich) and 1 μg/mL ionomycin (Sigma-Aldrich) for 2 hours and then treated with brefeldin A and monensin cocktail (eBioscience) for a further 3 hours. After stimulation, cells were washed with cold fluorescence activated cell sorting (FACS) buffer (PBS supplemented with 1% FBS). After blocking with Fc receptor binding inhibitor (clone: 93, eBioscience) at 1:250 dilution on ice for 20 min, cells were stained with antibodies against CD19 (clone: 6D5, fluorescein isothiocyanate (FITC)-conjugated, BioLegend), CD4 (clone: RM4-5, allophycocyanin (APC)-cyanine (Cy) 7-conjugated, BioLegend), CD44 (clone: IM7, phycoerythrin (PE)-Cy7-conjugated, BioLegend), PD-1 (clone: J43, APC-conjugated, BD Biosciences) and CXCR5 (clone: L138D7, peridinin chlorophyll protein (PerCP)-Cy5.5-conjugated, BioLegend) at 1:200 dilution on ice for 20 min. Following fixation and permeabilization (eBioscience), cells were further stained with antibodies against Foxp3 (clone: FJK-16S, FITC-conjugated, eBioscience), interleukin (IL)-4 (clone: 11B11, PE-conjugated, BioLegend) and interferon (IFN)-γ (clone: XMG1.2, BV42-conjugated, BioLegend) at 1:100 dilution on ice for 30 min. The samples were analyzed on a FACSCanto II and flow cytometric data analysis was done on FlowJo (ver.10.5.0).

Antigen-specific stimulation for identifying IL-21 or granzyme B producing T cell

2.5×10^6 cells per ml cells from inguinal and popliteal lymph nodes were stimulated with 1.5

μg/mL SARS-CoV-2 S protein peptide pool (PM-WCPV-S, JPT) and 2.5 μg/mL anti-CD28 mAb (clone: 37.51, Invitrogen) for 48 hours. Four hours before harvesting the cells, the cells were further treated with 50 ng/mL PMA, 1 μg/mL ionomycin, brefeldin A, and monensin cocktail. Cells were harvested, washed with FACS buffer as described above, and then blocked with Fc receptor binding inhibitor for 20 min. To identify IL-21 production, cells were stained with antibodies against CD19 (clone: 6D5, Pacific Blue-conjugated, BioLegend), CD4, CD44, PD-1, and CXCR5 at 1:200 dilution on ice for 20 min. Following fixation and permeabilization, cells were further stained with antibodies against Foxp3 and IL-21 (clone: mhalx21, PE-conjugated, Invitrogen) at 1:100 dilution on ice for 30 min. To identify granzyme B production by CD8 T cells, cells were stained with CD8 (clone: 53-6.7, Pacific Blue-conjugated, BD Biosciences), B220 (clone: RA3-6B2, APC-Cy7-conjugated, BD), CD3 (clone: 145-2C11, PE-conjugated, BD Biosciences) and CD49b (clone: DX5, PerCP-Cy5.5-conjugated, BioLegend) at 1:200 dilution on ice for 20 min. After fixation and permeabilization, cells were further stained with antibodies against granzyme B (clone: NGZB, eFluor 660-conjugated, eBioscience) at 1:100 dilution on ice for 30 min. The stained samples were analyzed using a FACSCanto II and the acquired data were done using FlowJo (ver.10.5.0).

FACS analysis and sorting of S protein-specific B cells

Splenocytes isolated from S_{MG} immunized mice were incubated with 2 μg/ml S protein at 4 °C for 1 hour, followed by washing and incubation with an antibody cocktail against CD19 (clone: 6D5, PE-Cy7-conjugated, BioLegend), CD3 (clone: 17A2, PE-conjugated, BioLegend), and His (clone: J095G46, APC-conjugated, BioLegend) at 1:125 dilution on ice for 15 min. Propidium Iodide (BioLegend) was used at 1:1000 to exclude dead cells. Live single S protein-specific B cells ($CD3^{-}CD19^{+}$) were sorted into 96-well PCR plates (Applied Biosystems) containing 10

μl/well catch buffer (10 mM Tris-HCl, pH 8, and 5 U/μl RNasin (Promega)) by BD FACSAria II. For repertoire analysis, five spleens from S_{MG} or S_{FG} immunized mice were pooled and stained before sorting.

Histopathology, immunohistochemistry (IHC), and immunofluorescence (IF) staining

Hamster lungs at 3 days post infection (dpi) or transgenic mice lungs at 7 dpi were freshly collected and immediately fixed by 10% neutral buffered formalin for 24 hours, and then transferred into 70% ethanol for 72 hours. Paraffin-embedded tissues were trimmed to a thickness of 5 mm. For histological staining, tissue was stained with hematoxylin and eosin (H & E) followed by microscopic examination. For IHC staining, the tissue sections were deparaffinized with xylene and rehydrated with an ethanol gradient. Antigen retrieval was performed by heating the slides to 95°C for 10 min in 10 mM sodium citrate buffer (pH 6.0) in a microwave oven. After cooling at room temperature and washing with PBS, 3% H_2O_2 was applied to eliminate endogenous peroxidase activity. Tissues were sectioned and blocked with 5% normal goat serum and 1 % BSA in 1x PBST for 1 hour, followed by incubation with rabbit anti-N primary antibody at 1:50 dilution (rabbit polyclonal antibody raised against N protein of SARS-CoV-2 (WH01), a gift from An-Suei Yang lab, Academia Sinica) overnight at 4°C, and then incubated with goat anti-rabbit HRP secondary antibody at 1:500 dilution for 1 hour. HRP activity was visualized by incubation with 3,3'Diaminobenzidine substrate and counterstained with hematoxylin. For immunofluorescence staining, after antigen retrieval steps, tissue was permeabilized with 0.1% Triton X-100 in PBS. Tissues were sectioned and blocked with 5% normal goat serum and 1% BSA in 1x PBST for 1 hour, and then incubated with an autofluorescence quencher for 5 min. The samples were subsequently incubated with rabbit anti-N primary antibody same as described above at 1:50 dilution for overnight at 4°C, then with

secondary antibody Alexa Fluor-488 (1:500, Thermo Fisher Scientific) for 1 hour at room temperature. Samples were then stained with 4,6-diamidino-2-phenylindole (DAPI) for 3 min. Coverslips were mounted on microscope slides and the slides were imaged under a Leica TCS SP8X confocal microscope with HC PL APO CS2 10x/0.40 lens (Leica AG).

Quantification of viral titer in lung tissue by cell culture infection assay ($TCID_{50}$).

The middle, inferior, and post-caval lung lobes of hamsters or transgenic mice were homogenized in 4 ml of DMEM with 2% FBS and 1% penicillin/streptomycin using a homogenizer. Tissue homogenate was centrifuged, and the supernatant was collected for live virus titration. Briefly, 10-fold serial dilutions of each sample were added onto Vero E6 cell monolayers in duplicate and incubated for 4 days, and cells were observed by microscope daily. The plates were washed with water and scored for infection. The fifty-percent tissue culture infectious dose ($TCID_{50}$) /ml was calculated by the Reed and Muench method.

ELISA determination of m31A7 binding to SARS-CoV-2 S protein or domains

For m31A7 binding ELISA assay, plates were coated with 0.2 μg per well SARS-CoV-2 S protein, 0.1 μg per well S1 (SinoBiological) or S2 (SinoBiological), or 0.05 μg per well NTD (SinoBiological) or RBD (SinoBiological). Plates were then blocked with PBST containing 2% BSA for 1 hour and washed 3 times with PBST before being incubated with 100 μl of m31A7 in 4-fold serial dilutions at 37 ºC for 1 hour. After three washes with PBST, the plates were incubated with 100 μl of HRP-conjugated goat anti-human IgG (1:10000) (Jackson Immuno-Research) for 1 hour at 37 ºC. The plates were washed 7 times with PBST and developed with 100 μl of the 1:1 mixed TMB substrate reagent set (BD Biosciences) for 10 min at 25°C. The reaction was stopped with 100 μl of 1 M H_2SO_4 and the OD450 was measured by SpectraMax Paradigm, Molecular Devices

Binding of antibody with S protein expressing 293T surface

HEK293T cells were transfected with pcDNA6/S protein-P2A-eGFP of SARS-CoV-2 WT or variants. Transfected cells were selected under 10 μg/ml of blasticidin for 2 to 3 weeks. Selected cells were then sorted by FACS Aria II to obtain $eGFP^+$ cells. These cells were maintained in DMEM containing 10% FBS and 10 μg/ml of blasticidin. Cells were incubated at a concentration of 2 to 3×10^5 cells with serially diluted antibody in FACS buffer on ice for 1 hour. Then, cells were washed with FACS buffer 3 times, followed by staining in brilliant violet 421 mouse anti-human IgG (BD Biosciences, 562581) at 1:100 dilution on ice for 20 min. Cells were then washed with FACS buffer twice. The percentage of positive cells was quantified using FACS Canto II and data was analyzed with FlowJo (ver.10.5.0).

Affinity and avidity determination using bio-layer interferometry

S protein (SARS-CoV-2 WT S2P) was conjugated with biotin by using One-Step Antibody Biotinylation Kit (Miltenyi Biotec) according to manufacturer's instructions. Biotinylated S protein was loaded at 5 μg/ml kinetics buffer (0.01% endotoxin-free BSA, 0.002% Tween-20, 0.005% NaN_3 in PBS) onto streptavidin biosensors (Molecular Devices, ForteBio). Fab fragment was prepared by using Pierce Fab Micro Preparation Kit (Thermo Fisher Scientific) according to manufacturer's instructions. Briefly, m31A7 IgG (250 μg) was digested by incubating with immobilized papain resin at 37°C for 8 hours. Fab was then purified by a protein A column. Association and dissociation of IgG or Fab by biotinylated S protein was performed in kinetics buffer at indicated concentrations for 5 min and 10 min, respectively. Dissociation constant (K_D) values were calculated using a 1:1 global fit model (Octet).

Hydrogen–Deuterium Exchange Mass spectrometry (HDX-MS)

HDX-MS experiments were carried out using an automated HDX robot (LEAP Technologies) coupled to an M-Class Acquity LC and HDX manager (Waters). Three µl protein solution containing 100 µM of RBD alone or RBD with m31A7 in PBS buffer was added to 57 µl labelling buffer (PBS in D2O pD 7.4) and incubated at 4°C for 15, 50, 100, 1000, 10000, or 20000 seconds. Following the labelling reaction, samples were quenched by adding 50 µL of the labelled solution to 50 µl quench buffer (0.2% formic acid, 200 mM TCEP, 8M urea in PBS, pH 2.3) giving a final quench pH of about 2.5. Fifty µL of quenched sample was passed through a Enzymate BEH Pepsin column (Waters) at 70 µl/min (15°C) and a VanGuard Pre-column Acquity UPLC BEH C18 (1.7 µm, 2.1 mm × 5 mm, Waters) for 3 min in 0.2 % formic acid in water. The pepsin-digested peptides were transferred to a C18 column (75 µm × 150 mm, Waters) and separated by a segmented gradient in 7 min from 5% to 40% solvent B (acetonitrile with 0.2% formic acid) at 40 µl/min (4°C). Solvent A was 0.2% formic acid in water. MS acquisitions were performed in positive and sensitivity mode in the m/z range 50–2000 Da on a Synapt G2 HDMS mass spectrometer (Waters) with a standard electrospray ionization source by Academia Sinica Common Mass Spectrometry Facility. The peptides were identified from MS^E analysis (the Engine that drives MS performance, Waters) and intermittent infusion of (Glu1)-fibrinopeptide B human (CAS No 103213-49-6, Sigma-Aldrich) was used for locking mass correction with an expected m/z (785.8426). HDX data were analyzed using PLGS (v3.0.2) and DynamX (v3.0.0) software supplied with the mass spectrometer. Restrictions for identified peptides in DynamX were as follows: minimum intensity 1000, max ppm error 25, file threshold 2/3.

Cryo-EM sample preparation, data collection, processing, and model building

The purified SARS CoV-2 S protein (D614G, 1 mg/ml) was mixed with m31A7 Fab (2 mg/ml) at 3:1 (v/v) ratio in 20mM Tris pH 8.0, 150mM NaCl for 3 min at room temperature. Mixed protein complex (4 µl) was applied to a glow discharged Quantifoil R1.2/1.3 holey carbon grid mounted in a Mark IV Vitrobot (Thermo Fisher Scientific) equilibrated at 4 °C and 100 % humidity. Grids were blotted at force 0 for 3 seconds. Data was collected on a Titan Krios G3 at 300 kV (Thermo Fisher Scientific), equipped with a Gatan K3 detector and the Gif Quantum energy filter with 20 eV slit width. Movies were acquired in EPU (Thermo Fisher Scientific, v2.10) at two exposures per hole. Total electron dose was 38 e- /A^2 collected over 2.5 sec and fractionated into 40 frames. The corresponding pixel size was 0.83 Å in the defocus range from -1.5 to -2 µm. 4311 micrographs were harvested (**fig. S16A**) and processed with C1 symmetry in relion 3.0 (*49*) with the resolution of the final map determined by gold-standard Fourier shell correlation (FSC) cutoff at 0.143 (**fig. S16B, data file S1**). The coordinates used for model fit in map included the S protein (PDB 7CN9) and the crystal structure of Fab-RBD complex solved in this study (PDB 7WUE).

Crystallization, data collection and structure determination of m31A7-Fab-bound RBD.

The RBD domain (333-530) of SARS-CoV-2 WT S protein was constructed and cloned into pTT vector for protein expression and purification. The plasmid was mixed with PEI (polyethyleneimine) and transfected into HEK293EBNA (ATCC CRL-10852) suspension cells shaking for 4 days at 37°C. The supernatant was harvested and clarified by centrifugation before being loaded onto Ni-NTA resin (GE healthcare). The eluant was concentrated in Millipore Amicon Ultra Filter (MW 10 kDa cutoff) and further purified by Superdex 200 SEC (10/300 GL, GE healthcare) preequilibrated with 20 mM Tris/HCl, pH 8.0, 150 mM NaCl. The antibody

m31A7 was purified and Fab fragments was digested as mentioned above. The purified RBD and m31A7-Fab were mixed at 1:1 molar ratio for 30 min on ice before further purification by Superdex 200 SEC (10/300 GL, GE healthcare) in 20 mM Tris/HCl, pH 8.0, 150 mM NaCl. The complex peak was checked with 14% SDS-PAGE and pooled together to be concentrated to 12.5 mg/ml. Hanging-drop vapor diffusion crystallization was set at 20°C and the crystals appeared on day 8 in the condition containing 2.0 M Ammonia sulfate, 0.1M sodium acetate pH 4.6. Crystals were harvested and diffraction datasets collected at National Synchrotron Radiation Research Center (NSRRC) BL15A1 beamline at resolution 3.2Å in the space group of $C_{2\,2\,21}$. Data was processed in iMosflm (*50*) ; Molecular replacement was performed by Phenix Phase-MR (*51*) using PDB 7C01 as the searching ensemble. The solved structure was further refined in phenix.refine with manual adjustments done in WinCoot (*52*) until R/Rfree converged and all geometry statistics were satisfactory (**data file S1**).

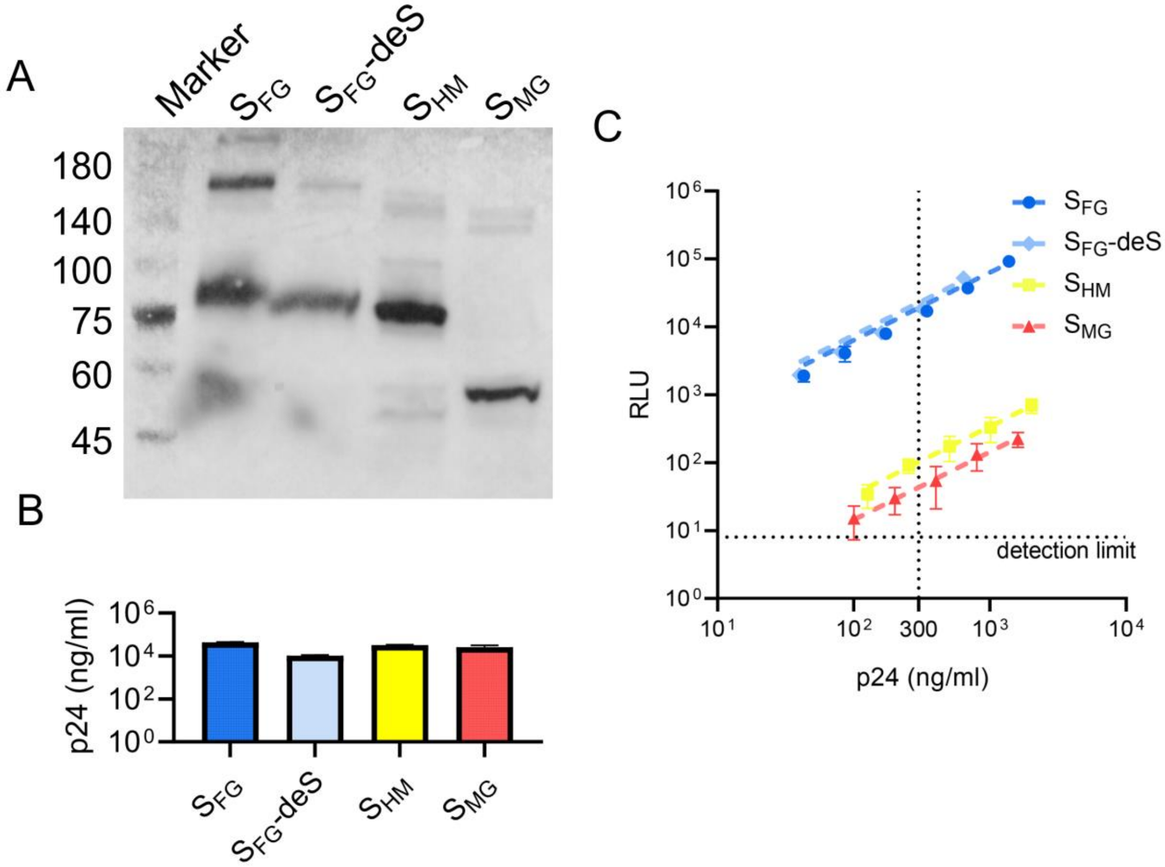

Fig. S1. Virus production and infection of pseudovirus carrying glycoengineered spike (S) protein from severe acute respiratory syndrome coronavirus 2 (SARS-CoV-2). (A) Western blot analysis of the original and the glycoengineered pseudoviruses is shown using rabbit anti-S protein polyclonal antibody. $S_{FG,}$ fully glycosylated; $S_{FG,}$-deS, non-sialylated; $S_{HM,}$ high-mannose; S_{MG}, Mono-GlcNac. **(B)** p24 quantification (duplicates) of glycoengineered pseudoviruses is shown. **(C)** The infectivity analysis of glycoengineered pseudoviruses is shown (6 replicates). The y-axis shows the virus amount (p24-equivalent) used for infection and the vertical line (at 300 ng/ml p24) represents the quantity applied in the Fig. 1D. RLU, relative light units. Data are presented as mean ± SD.

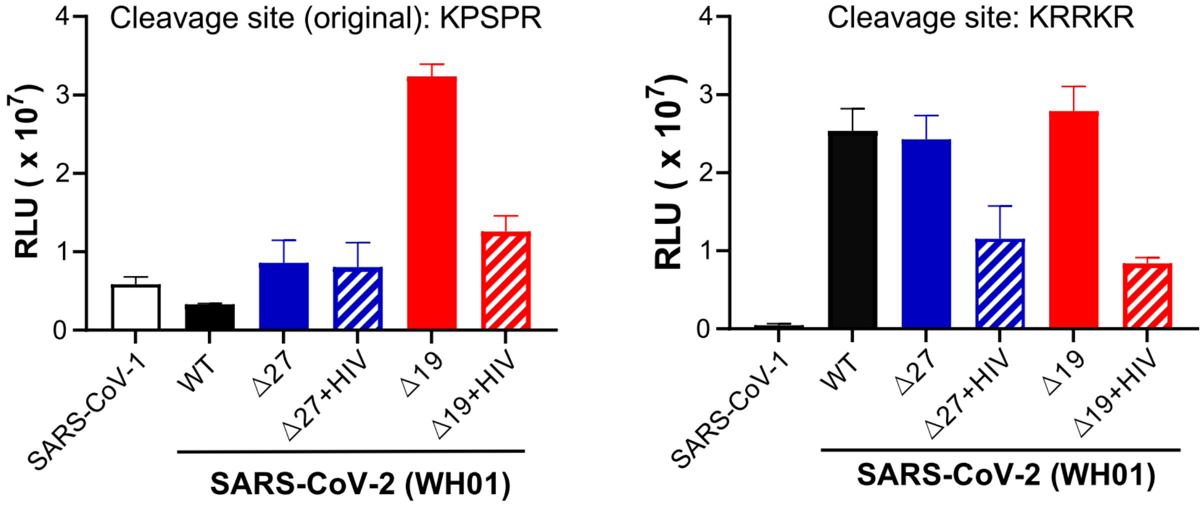

Fig. S2. Impact of cytoplasmic truncation of S protein on pseudovirus production. Infectivity analysis (measured as RLU) shows the titer of pseudovirus carrying S protein from SARS-CoV-1 (white) or SARS-CoV-2 wild type (WT, black), C-terminal 27 amino acids (aa) deletion mutant (Δ27, blue), C-terminal 19aa deletion (Δ19, red), and Δ27 or Δ19 with the addition of HIV sequence NRVRQGYS (striped blue or striped red), with or without arginine substitutions at 812 and 813 (KRRKR) to facilitate efficient pseudovirus formation. Data of 3 replicates are presented as mean ± SD.

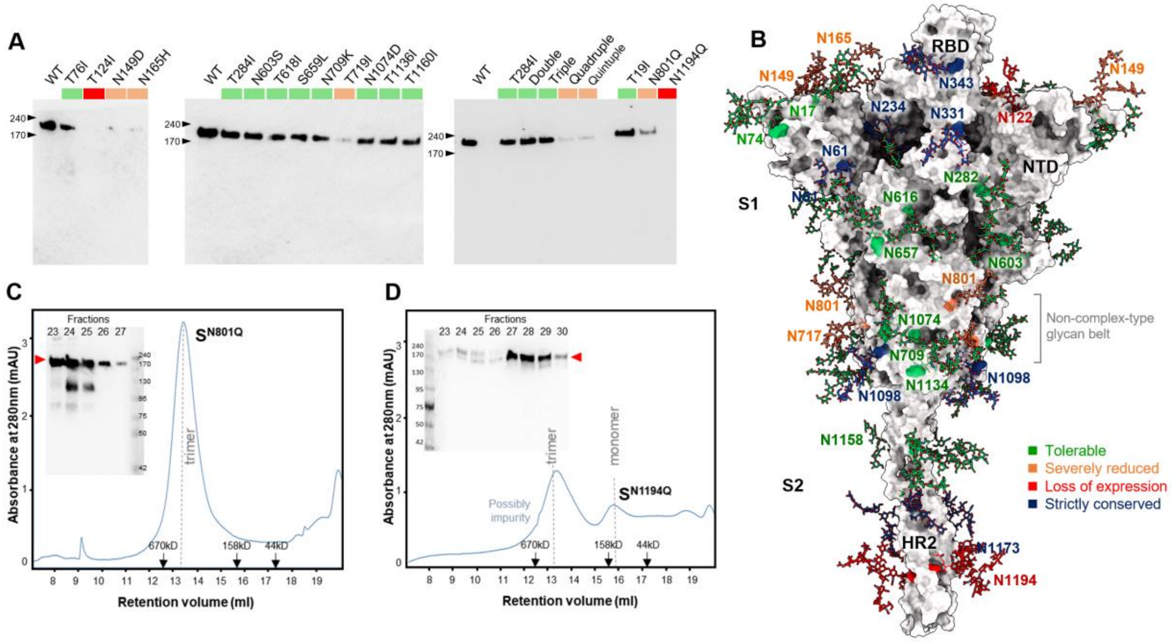

Fig. S3. Expression of recombinant S protein with N-glycosite mutations. (A) Western blot analysis using anti-(his)$_6$ monoclonal antibody shows different expression of each N-glycosite single or multiple mutants as labeled. Multiple mutants: double, T284I/N603S; triple, T284I/N603S/T618I; quadruple, T284I/N603S/T618I/S659L; quintuple, T284I/N603S/T618I/S659L/T1136I. Colored blocks indicate relative expression, green: normal or slightly reduced expression, orange: severe reduction, red: almost no expression. **(B)** Mutational tolerance mapping of each tested N-glycosite in terms of protein yield is shown on modelled S structure, colored according to (A). Dark blue indicates strictly conserved sites which were not tested. **(C and D)** Size-exclusion chromatography profile of S protein N801Q (C) and N1194Q (D) mutants are shown, with fractions analyzed by western blot using rabbit anti-S polyclonal antibody (S protein highlighted as red triangles). The expression experiment is an initial test which had been performed once before we moved on to the S_{MG} vaccine design.

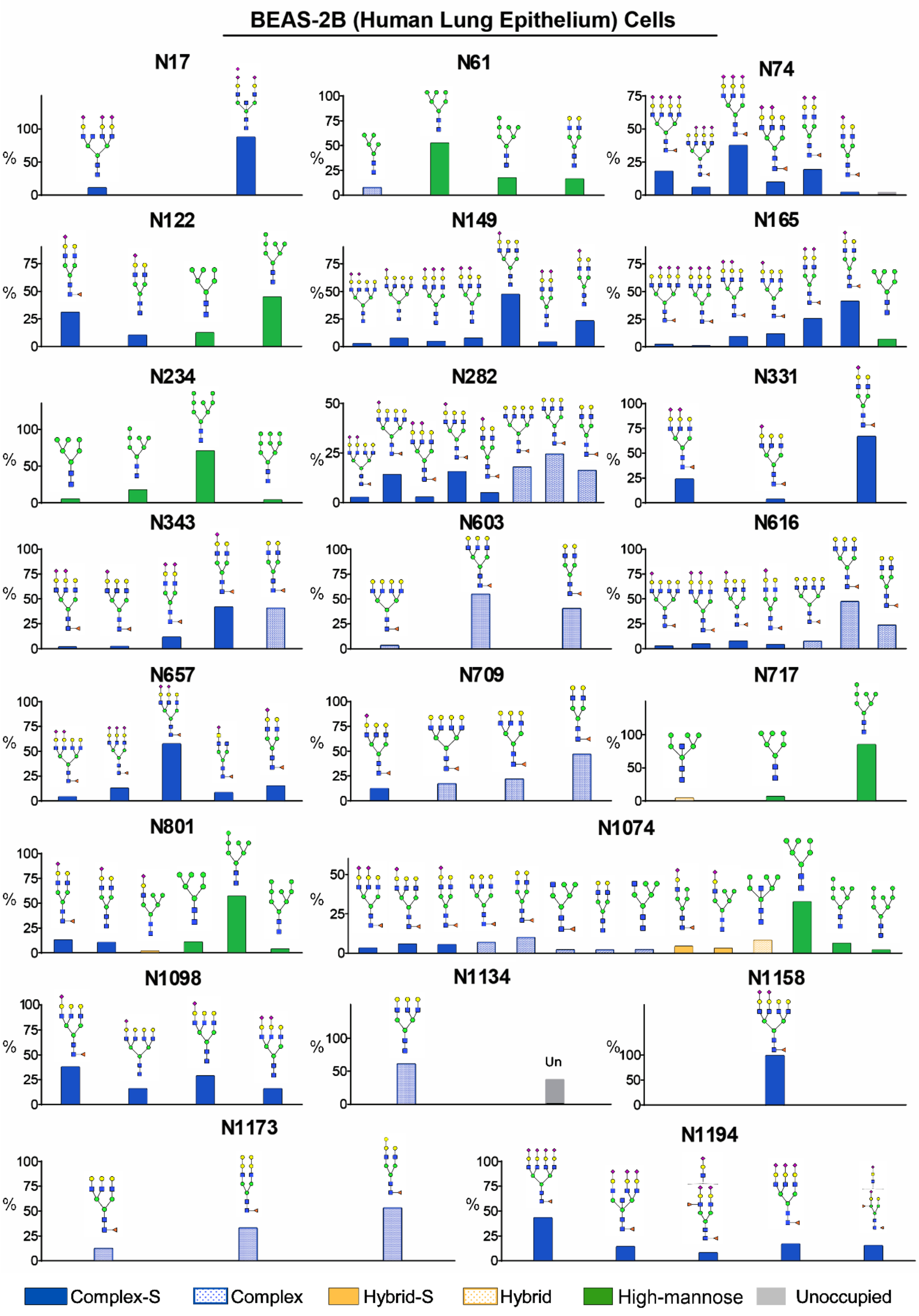
BEAS-2B (Human Lung Epithelium) Cells
N17
N61
N74
N122
N149
N165
N234
N282
N331
N343
N603
N616
N657
N709
N717
N801
N1074
N1098
N1134
Un
N1158
N1173
N1194
%
Complex-S
Complex
Hybrid-S
Hybrid
High-mannose
Unoccupied

Fig. S4. N-linked glycoforms of SARS-CoV-2 S protein from BEAS-2B cells. Detailed glycan profiles of S protein expressed from BEAS-2B cells was determined by the liquid chromatography coupled with tandem mass spectrometry (LC-MS/MS). Different glycans at each site are shown individually in structure with y-axis representing the percentage of the population. Each glycan is drawn according to standard monosaccharide symbol nomenclature. Each bar is colored by groups: complex-type with sialic acids (blue), complex-type (dotted blue), hybrid-type with sialic acids (yellow), hybrid-type (dotted yellow), high-mannose type (green) and unoccupied (gray). Three biological replicates were analyzed and the mean of the percentage are shown. Glycoforms with percentage less than 2% are omitted.

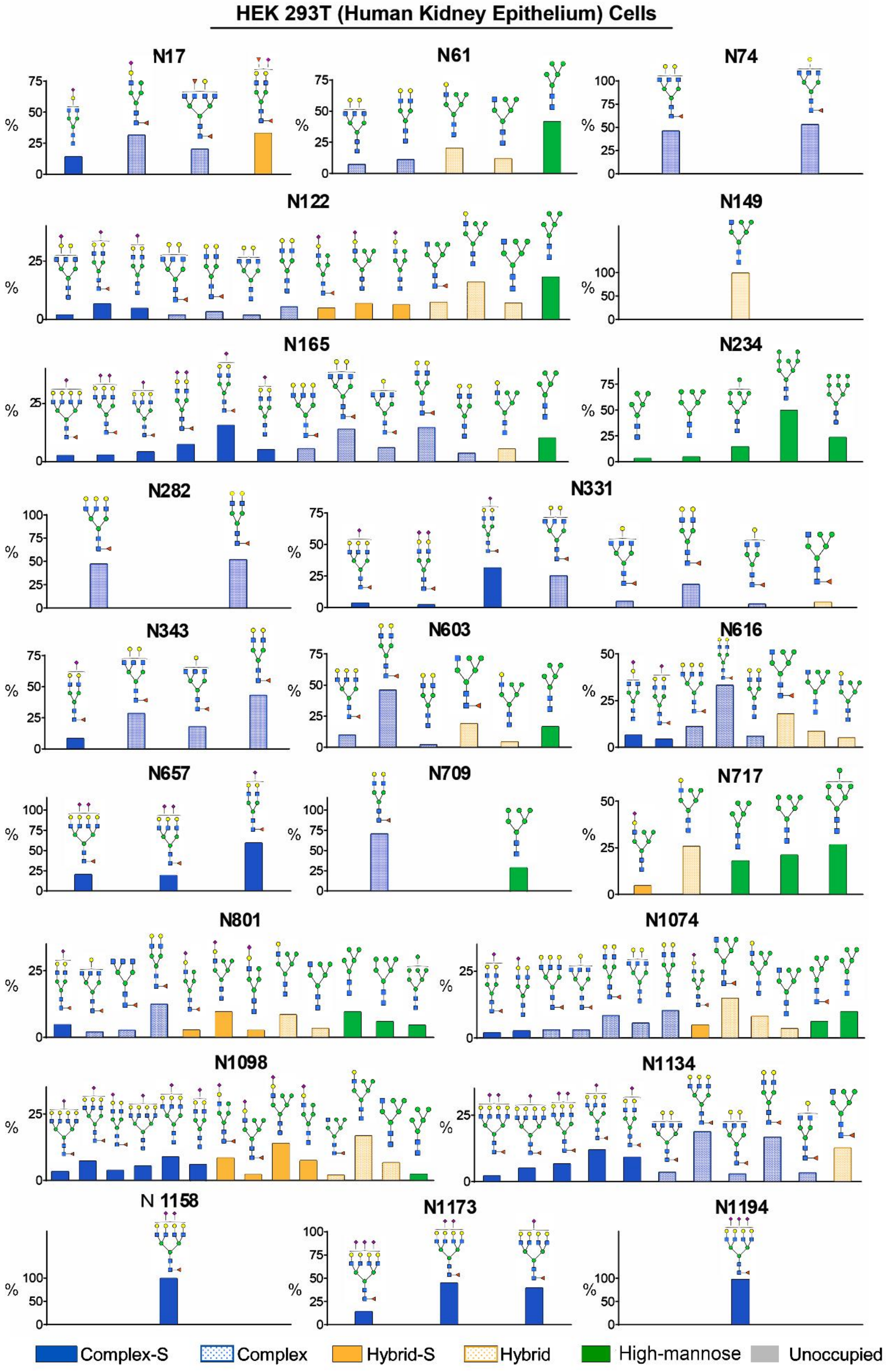
HEK 293T (Human Kidney Epithelium) Cells
N17
N61
N74
N122
N149
N165
N234
N282
N331
N343
N603
N616
N657
N709
N717
N801
N1074
N1098
N1134
N 1158
N1173
N1194
%
Complex-S
Complex
Hybrid-S
Hybrid
High-mannose
Unoccupied

Fig. S5. N-linked glycoforms of SARS-CoV-2 S protein from HEK293T cells. Detailed glycan profiles of S protein expressed from HEK293T cells was determined by LC–MS/MS. Different glycans at each site are shown individually in structure with y-axis representing the percentage of the population. Each glycan is drawn according to standard monosaccharide symbol nomenclature. Each bar is colored by groups: complex-type with sialic acids (blue), complex-type (dotted blue), hybrid-type with sialic acids (yellow), hybrid-type (dotted yellow), high-mannose type (green) and unoccupied (gray). Three biological replicates were analyzed and the mean of the percentage are shown. Glycoforms with percentage less than 2% are omitted.

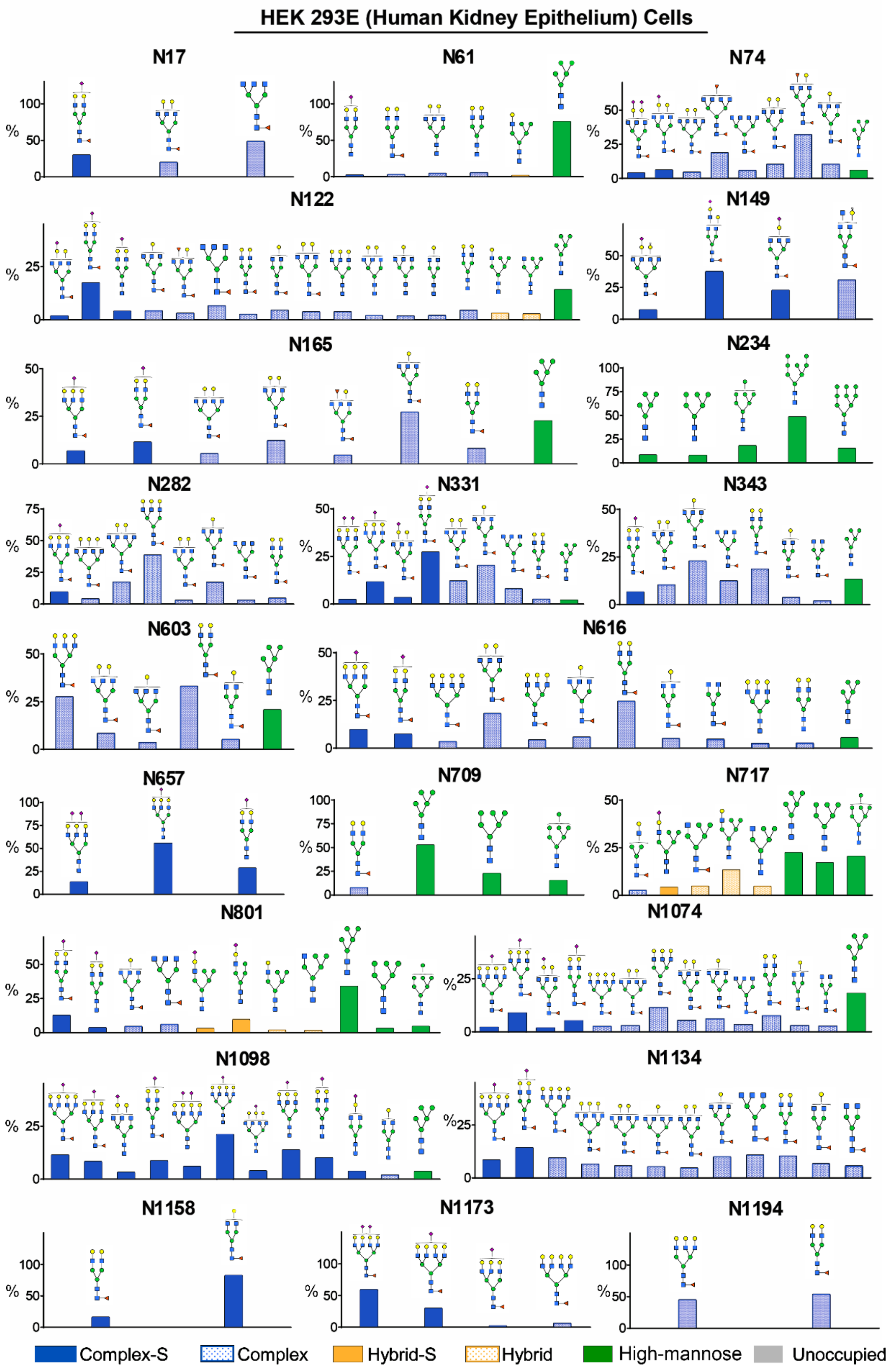
HEK 293E (Human Kidney Epithelium) Cells
N17
N61
N74
N122
N149
N165
N234
N282
N331
N343
N603
N616
N657
N709
N717
N801
N1074
N1098
N1134
N1158
N1173
N1194
%
Complex-S
Complex
Hybrid-S
Hybrid
High-mannose
Unoccupied

Fig. S6. N-linked glycoforms of SARS-CoV-2 S protein from HEK293E cells. Detailed glycan profiles of S protein expressed from HEK293 EBNA cells was determined by LC–MS/MS. Different glycans at each site are shown individually in structure with y-axis representing the percentage of the population. Each glycan is drawn according to standard monosaccharide symbol nomenclature. Each bar is colored by groups: complex-type with sialic acids (blue), complex-type (dotted blue), hybrid-type with sialic acids (yellow), hybrid-type (dotted yellow), high-mannose type (green) and unoccupied (gray). Three biological replicates were analyzed and the mean of the percentage are shown. Glycoforms with percentage less than 2% are omitted.

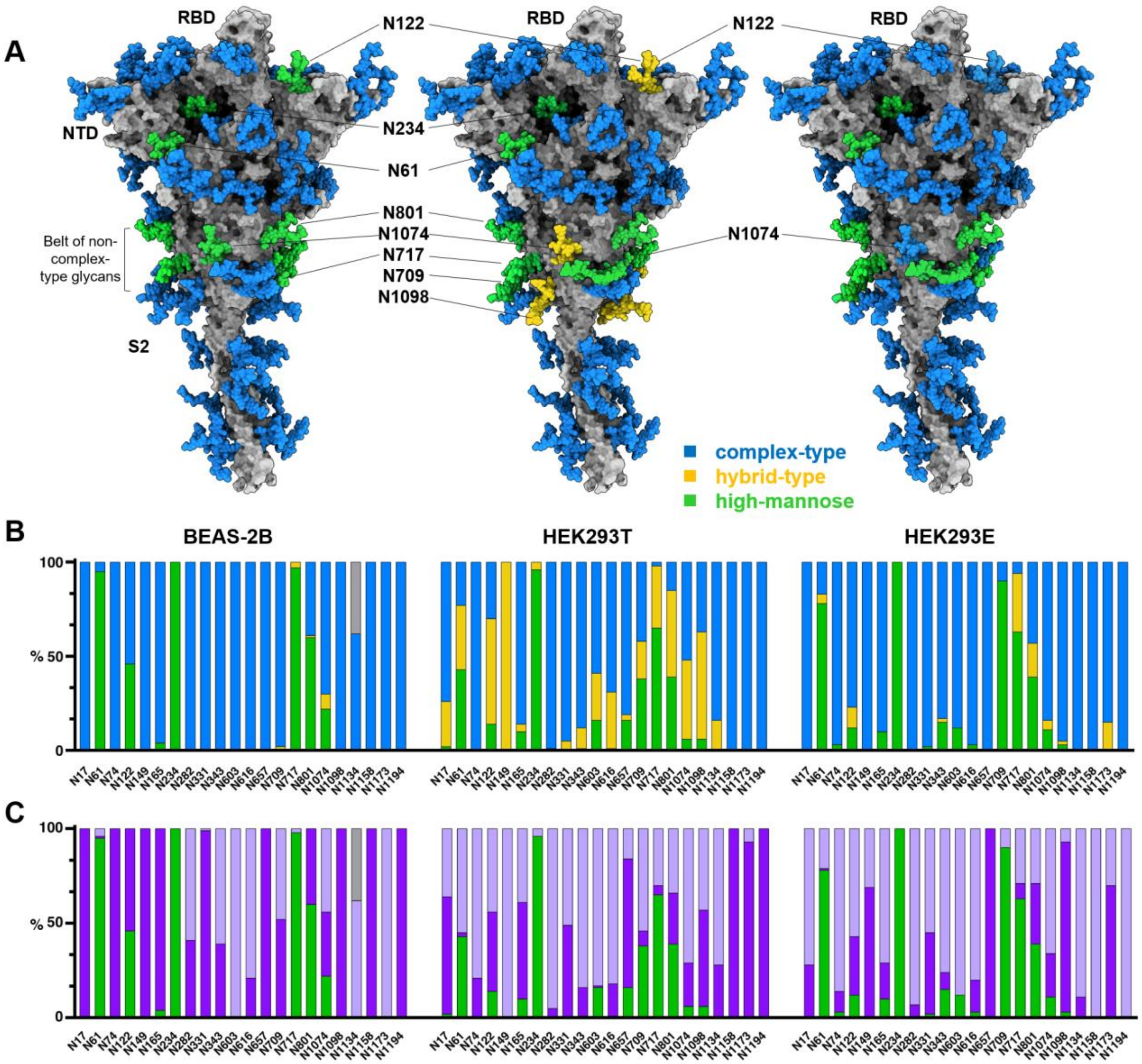

Fig. S7. Comparison of S protein glycan profiles from BEAS-2B, HEK293T, and HEK293E cells. (A) Mapping of S protein glycan profile (from fig. S4 to S6 data) on modeled 3D structure is shown, with the glycans colored by the highest-abundance type (complex-type in blue, hybrid-type yellow, high-mannose green). **(B and C)** Bar charts summarize the comparison of S protein glycan profiles from the three evaluated cell lines, either grouped by complex-type (blue), high mannose (green) and hybrid-type (yellow) (B) or by sialylated (purple), non-sialylated (lavender), high-mannose (green) and unoccupied (gray) (C). Each N-glycosite is labeled on the x-axis, and the percentage of each group is at y-axis.

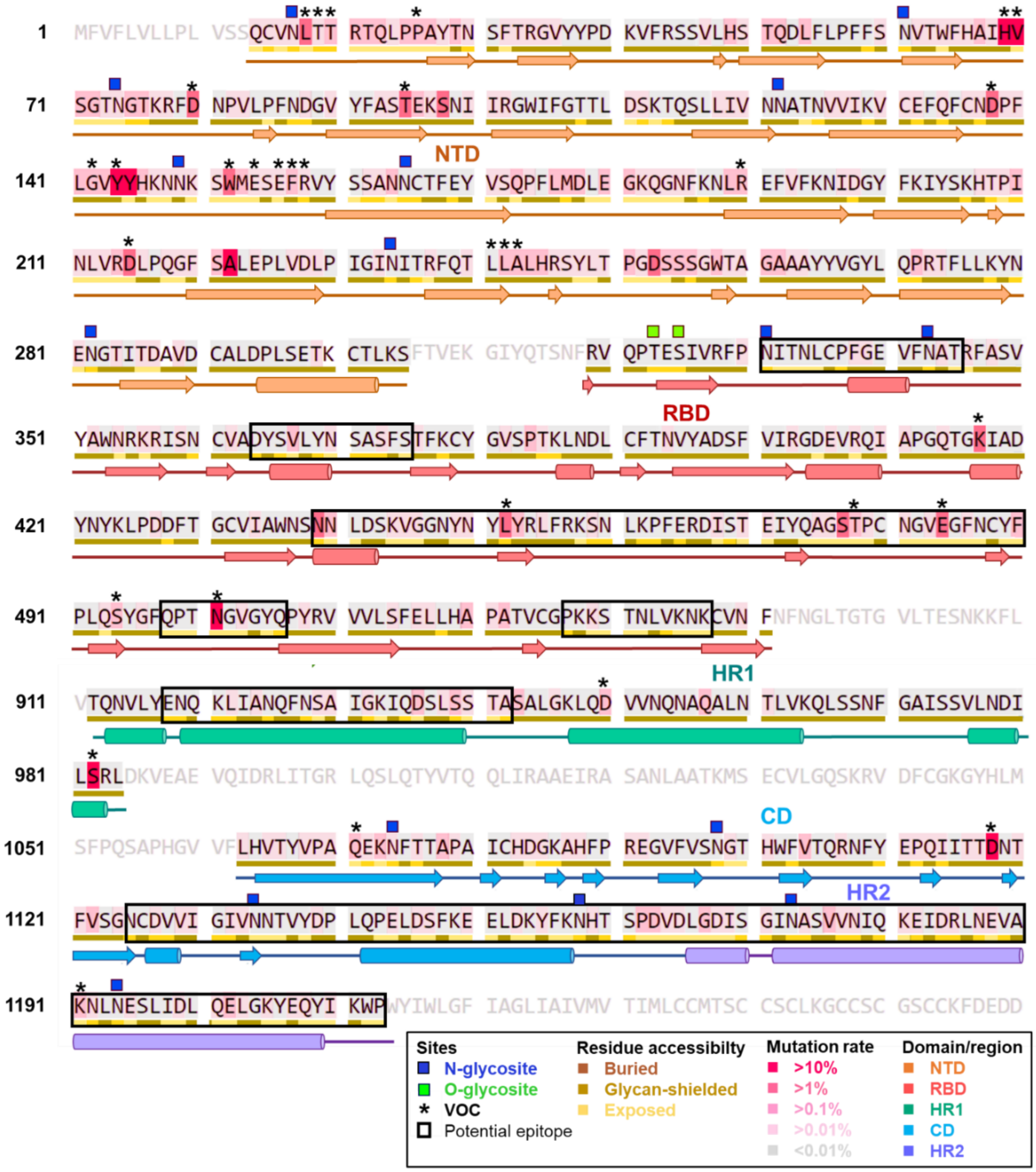

Fig. S8. Sequence variation overlaid with residue accessibility in selected regions of S protein. The primary sequence is shaded in a red gradient with the darker color for higher mutation rate. Below the sequence is a yellow-gradient line with the darker color for lower-accessibility (buried) residues. Secondary structures are drawn in cartoon colored by domains: N-terminal domain (NTD, orange), receptor binding domain (RBD, red), heptad repeat 1 (HR1, teal), connecting domain (CD, blue) and heptad repeat 2 (HR2, purple). Other symbols are shown in the box. N-glycosites: blue square; *O*-glycosites: green square; residues of variants of concern (VOCs): stars, including variants of Alpha, Beta, Gamma, Delta, B.1.427, B.1.429, B.1.617, B.1.617.1 (Kappa), and B.1.617.3 from the Centers for Disease Control and Prevention (CDC) official site. Black frames highlight potential linear epitopes with relatively low variation, high accessibility, and residue continuity. Mutation rate < 0.1% is considered as conserved and the relative solvent accessibility (RSA) >5% as exposed.

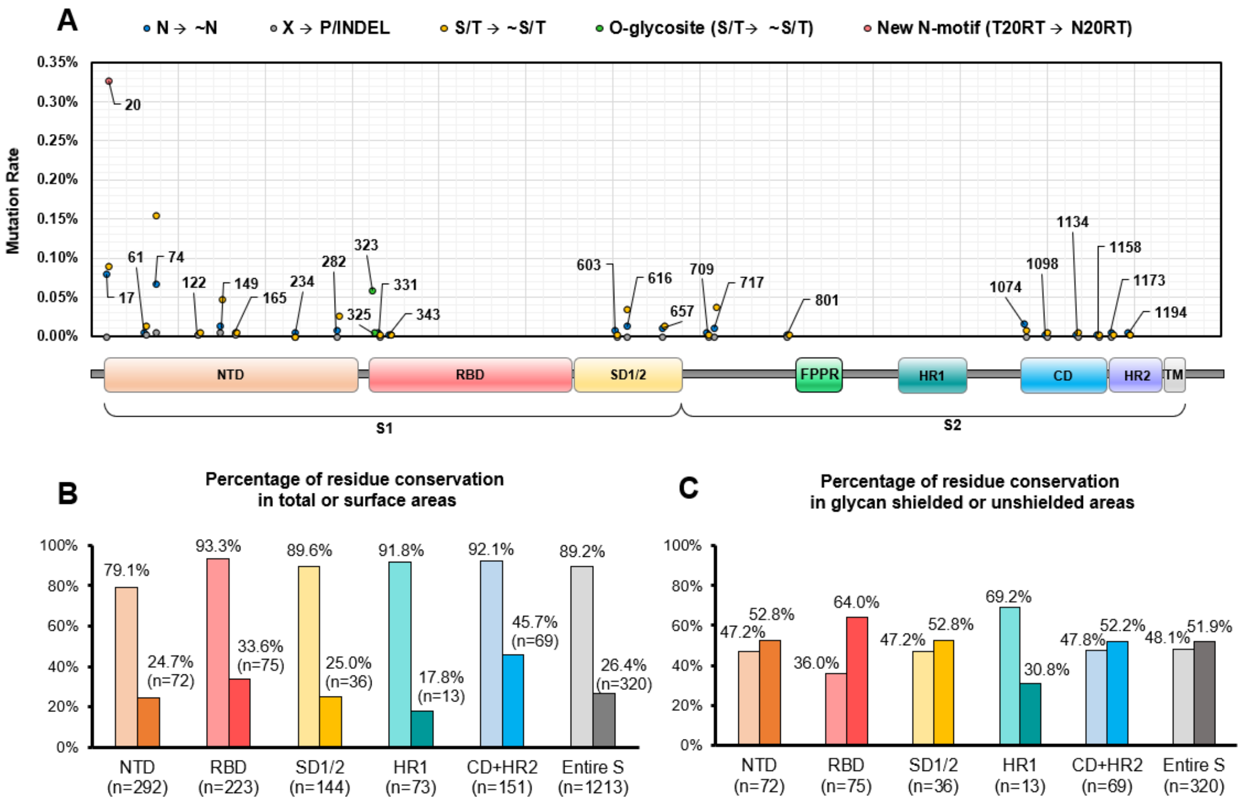

Fig. S9. Conservation of S protein glycosites and correlation with glycan shielding. (A) The mutation rate (%, y-axis) of 22 N-glycosites and 2 *O*-glycosites is shown in the context of domain organization of SARS-CoV-2 WT S protein below x-axis. Blue, gray, and yellow dots indicate the N-glycosite Asn–X–Ser/Thr sequon (N, X, and S/T) residues of each N-glycosite, respectively. Residue numbers are only shown for N. Green dots are *O*-glycosites. Pink indicate a new N-glycosite. **(B)** Percentages are shown for conserved residues (light colors) or conserved surface residues (dark colors) of total residues of each region, following the color scheme as in (A). **(C)** Percentages are shown for conserved surface residues shielded by glycans (light color) or not shielded by glycans (dark color) in surface residues of each region. Mutation rate < 0.1% is considered as conserved and RSA >5% as exposed. Statistics are based on the modelled S protein structure using the glycan profile from BEAS-2B expression.

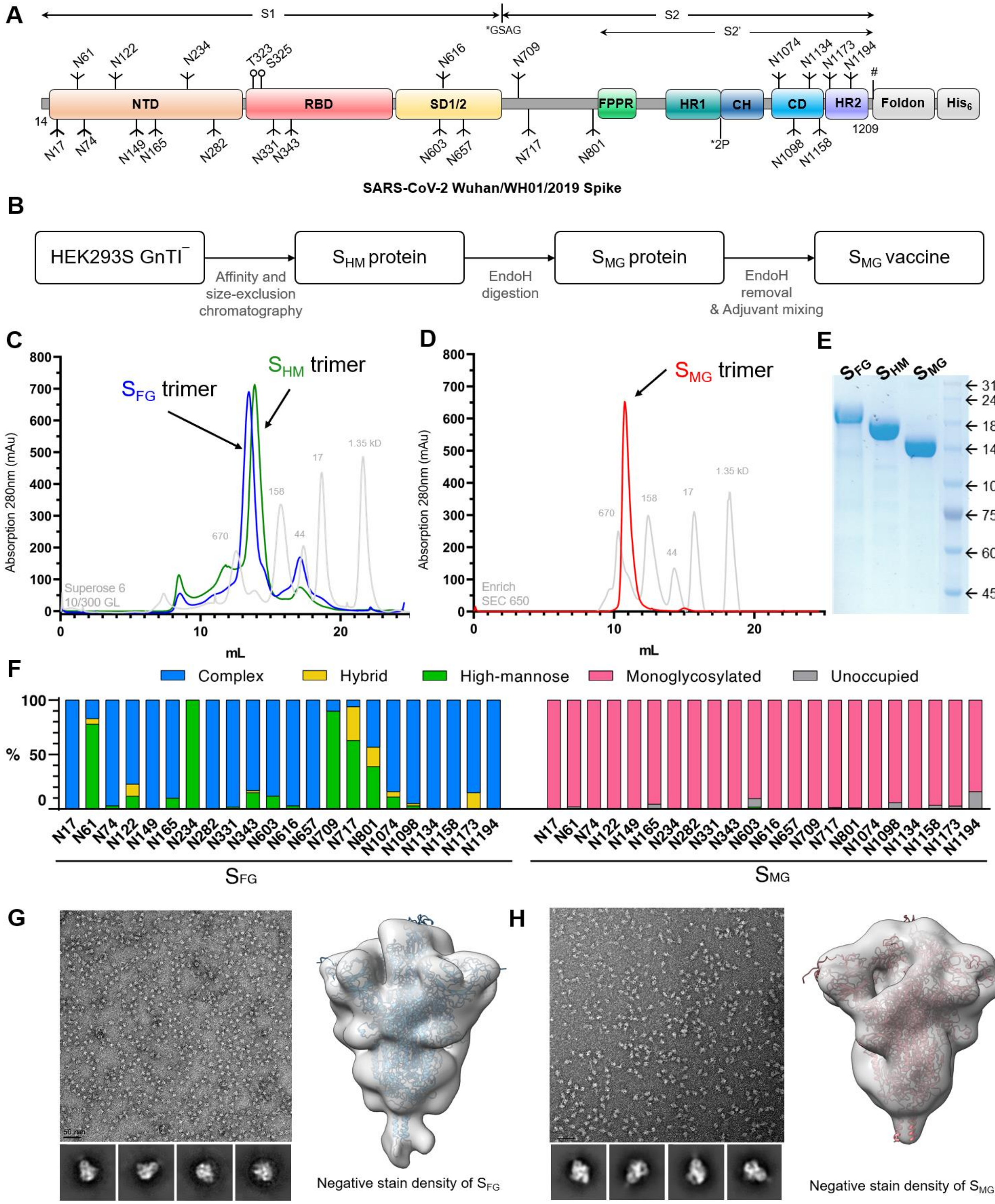

A
S1
S2
S2'
*GSAG
N61
N122
N234
T323
S325
N616
N709
N1074
N1134
N1173
N1194
#
NTD
RBD
SD1/2
FPPR
HR1
CH
CD
HR2
Foldon
His6
14
1209
N17
N74
N149
N165
N282
N331
N343
N603
N657
N717
N801
*2P
N1098
N1158
SARS-CoV-2 Wuhan/WH01/2019 Spike
B
HEK293S GnTI⁻
Affinity and size-exclusion chromatography
SHM protein
EndoH digestion
SMG protein
EndoH removal & Adjuvant mixing
SMG vaccine
C
SFG trimer
SHM trimer
Absorption 280nm (mAu)
670
158
44
17
1.35 kD
Superose 6 10/300 GL
mL
D
SMG trimer
Enrich SEC 650
E
SFG
SHM
SMG
310
245
180
140
100
75
60
45
F
Complex
Hybrid
High-mannose
Monoglycosylated
Unoccupied
%
SFG
SMG
G
50 nm
Negative stain density of SFG
H
Negative stain density of SMG

Fig. S10. Design and characterization of Mono-GlcNAc-decorated S protein (S_{MG}) vaccine. **(A)** Schematic representation of the recombinant SARS-CoV-2 S protein construct, colored by domain as in Fig. 1A. The C-terminus of soluble S protein is attached with a T4 fibritin (foldon) sequence and a His-tag (His_6). The furin cleavage site is substituted by GSAG and two proline residues are introduced in (K986P and V987P) to fix the S protein in the prefusion state. The # site represents the thrombin cleavage site. **(B)** Schematic overview of S_{MG} vaccine production. S_{HM}, S protein with high-mannose type N-glycans; S_{MG}, S protein with a single GlcNAc at each N-glycosite. **(C)** Size-exclusion chromatography profile of S_{FG} (S protein with the original complex-type N-glycans) and S_{HM}. Blue curve: $S_{FG,}$ Green curve: $S_{HM,}$ Gray curve: protein molecular weight markers. **(D)** Size-exclusion chromatography profile of purified $S_{MG.}$ Red curve: S_{MG}, Gray curve: protein molecular weight markers. **(E)** SDS-PAGE analysis of purified S_{FG}, S_{HM}, and S_{MG}. (C, D and E) show the representative data from greater than 3 independent experiments. **(F)** Mass spectrometry analysis of the N-glycan compositions of S_{FG} and S_{MG}, with blue color for complex-type, yellow for hybrid-type, green for high-mannose, pink for mono-GlcNAc and gray for unoccupied. Data are shown as the average of three replicates. **(G** and **H)** Negative stain raw image of purified S_{FG} (G) or S_{MG} (H) with selected 2D class averages shown below, and 3D reconstructed density on the right. The S protein modeled structure (as in Fig. 2C) was used for fitting, colored in blue for S_{FG} (G) and pink for S_{MG} (H), with the flexible HR2 regions unshown. Volume contoured at level 3.08.

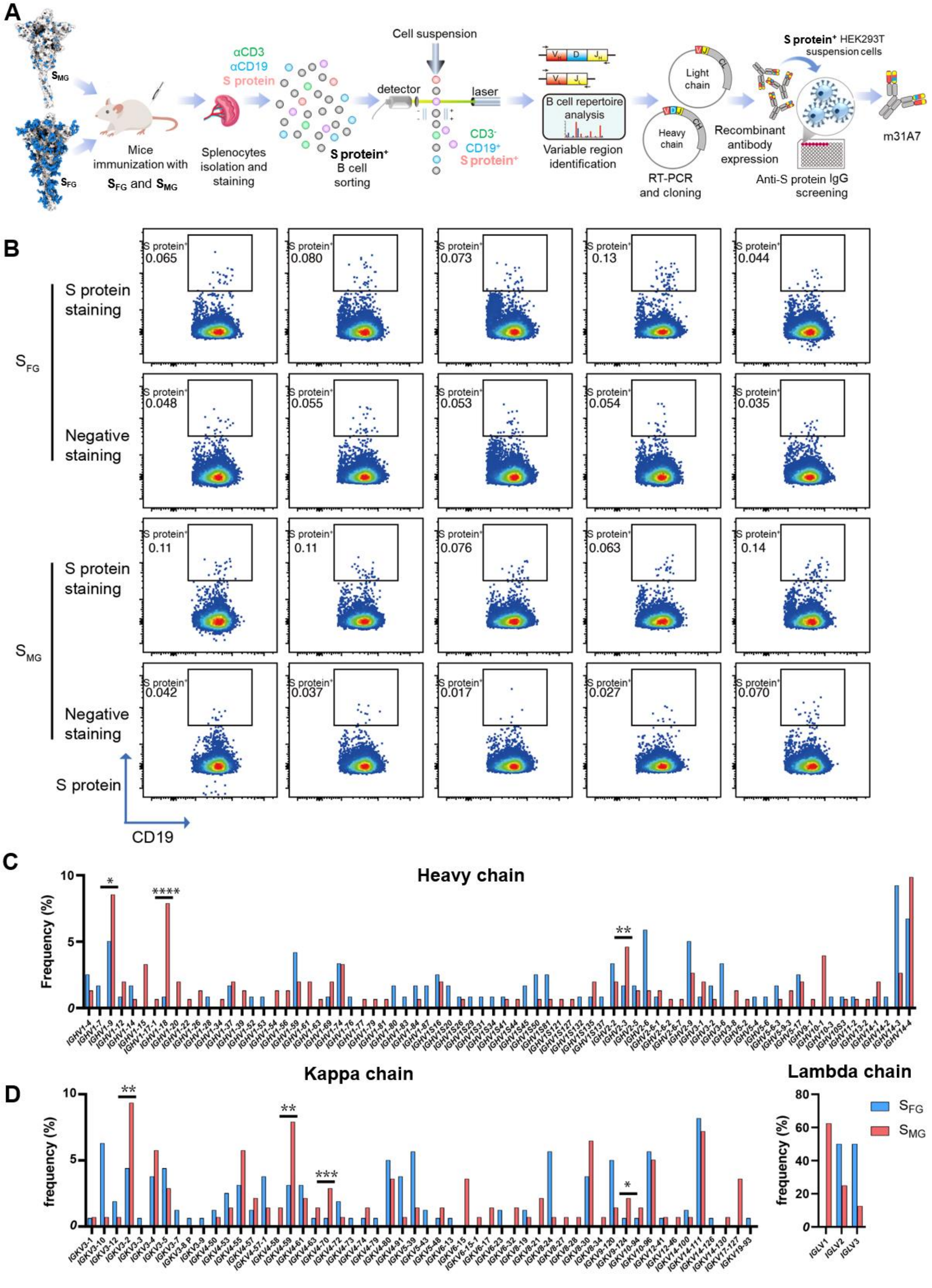
A
SMG
SFG
Mice immunization with SFG and SMG
αCD3
αCD19
S protein
Splenocytes isolation and staining
S protein+ B cell sorting
Cell suspension
detector
laser
CD3-
CD19+
S protein+
B cell repertoire analysis
Variable region identification
Light chain
Heavy chain
RT-PCR and cloning
S protein+ HEK293T suspension cells
Recombinant antibody expression
Anti-S protein IgG screening
m31A7
B
SFG
SMG
S protein staining
Negative staining
S protein+
0.065
0.080
0.073
0.13
0.044
0.048
0.055
0.053
0.054
0.035
0.11
0.11
0.076
0.063
0.14
0.042
0.037
0.017
0.027
0.070
S protein
CD19
C
Heavy chain
Frequency (%)
D
Kappa chain
Lambda chain
frequency (%)
SFG
SMG

Fig. S11. Locus usages of heavy and light chains of SARS-CoV-2-specific B cells isolated from S_{FG} and S_{MG} vaccinated mice.

(A) Overview of the single B cell screening platform. Single S protein-specific B cells ($CD3^{-}CD19^{+}$S protein^{+}) from spleens of immunized mice were sorted into 96-well plates by fluorescence activated cell sorting (FACS). The gene transcripts encoding the IgH and IgL chains for each single B cell were amplified by real-time polymerase chain reaction (RT-PCR). After sequencing, the cDNAs from the variable regions of IgH or IgL genes were subcloned into the expression vectors containing human IgG heavy chain or light chain constant region, respectively. The chimeric monoclonal antibody was produced by Expi293, and its binding to S protein-expressing 293T cells was measured by FACS. **(B)** FACS analysis shows the percentage of S protein-specific B cells from mice immunized with S_{FG} or S_{MG} at day 28 after the last immunization. **(C)** Analysis of heavy chain IgG repertoires of S_{FG} or S_{MG} immunized mice showed highly represented *IGHV1-9, IGHV1-18,* and *IGHV2-3* in the S_{MG} group (*P*-values: 0.048, 1.6×10^{-11} and 0.005). **(D)** Light chain (including kappa and lambda chain) IgG repertoires of S_{FG} or S_{MG} immunized mice showed highly represented *IGKV3-2, IGKV4-59, IGKV4-70,* and *IGKV9-142* in the S_{MG} group (*P*-values 0.044, 0.0013, 0.0008 and 0.0226). P values were obtained from a chi-squared test. $*P < 0.05$; $**P < 0.01$; $***P < 0.001$; $****P < 0.0001$.

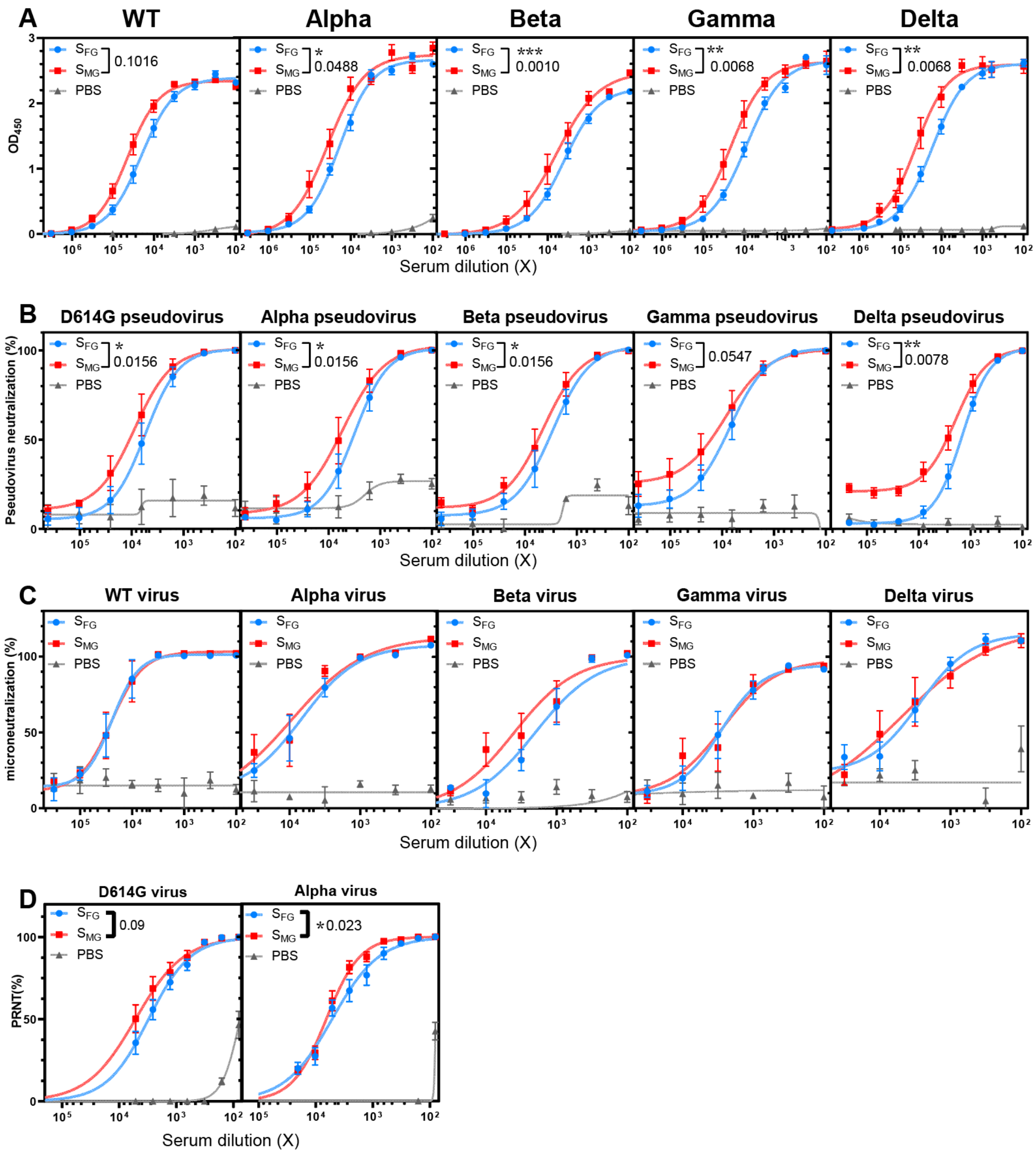

Fig. S12. Humoral response of S_{FG} and S_{MG} immunized mice against SARS-CoV-2 WT and variants. (**A to D**) ELISA binding curves (A), pseudovirus neutralization percentage (B), and authentic virus neutralization percentage using microneutralization (C) or PRNT assay (D) are shown for serum samples collected from vaccinated mice (n=5) and tested with SARS-CoV-2 WT and variants as labeled on top of each panel. Phosphate-buffered saline (PBS) was used as a negative control for vaccination. Data are presented as mean ± SEM. The curves were fitted with nonlinear regression using Graph Prism 9.0 and comparisons were performed by Wilcoxon matched-pairs signed rank test (two-tailed). **P* < 0.05; ***P* <0.01; ****P* < 0.001.

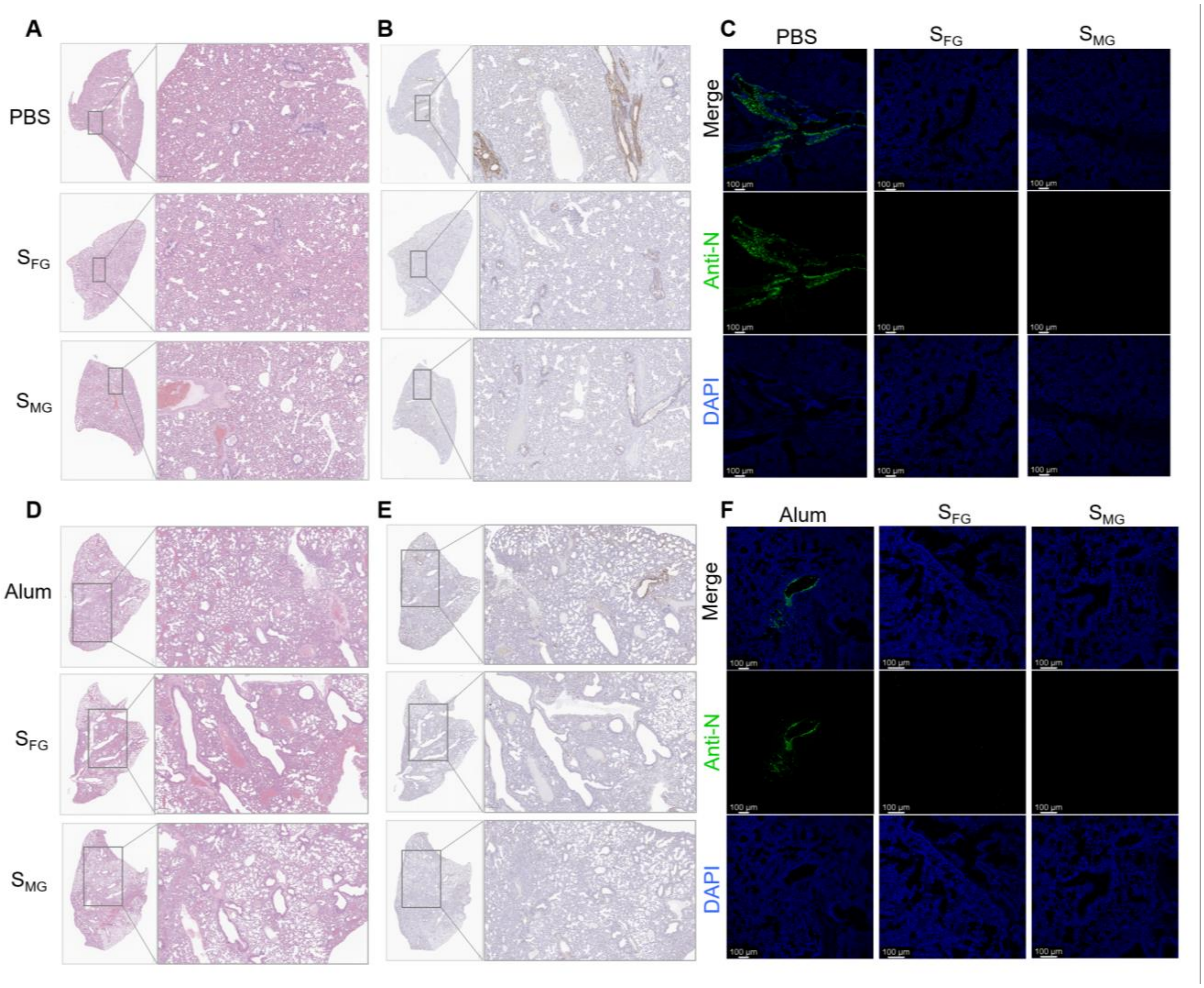

Fig. S13. Representative staining of SARS-CoV-2 lung infection in animal models. (A to C) Representative histopathology (H&E) staining (A), IHC staining (B) and SARS-CoV-2 N-specific IF staining (C) are shown for a SARS-CoV-2-infected hamster at 3 dpi (n=3). (**D to F**) H&E staining (D), IHC staining (E) and SARS-CoV-2 N-specific IF staining (F) are shown for a SARS-CoV-2-infected human angiotensin converting enzyme 2 (hACE2) transgenic mouse (7 dpi) (n=3). The scale bars for H&E and IHC staining are 1mm and 200 μm. The scale bar for immunofluorescence staining is 100 μm.

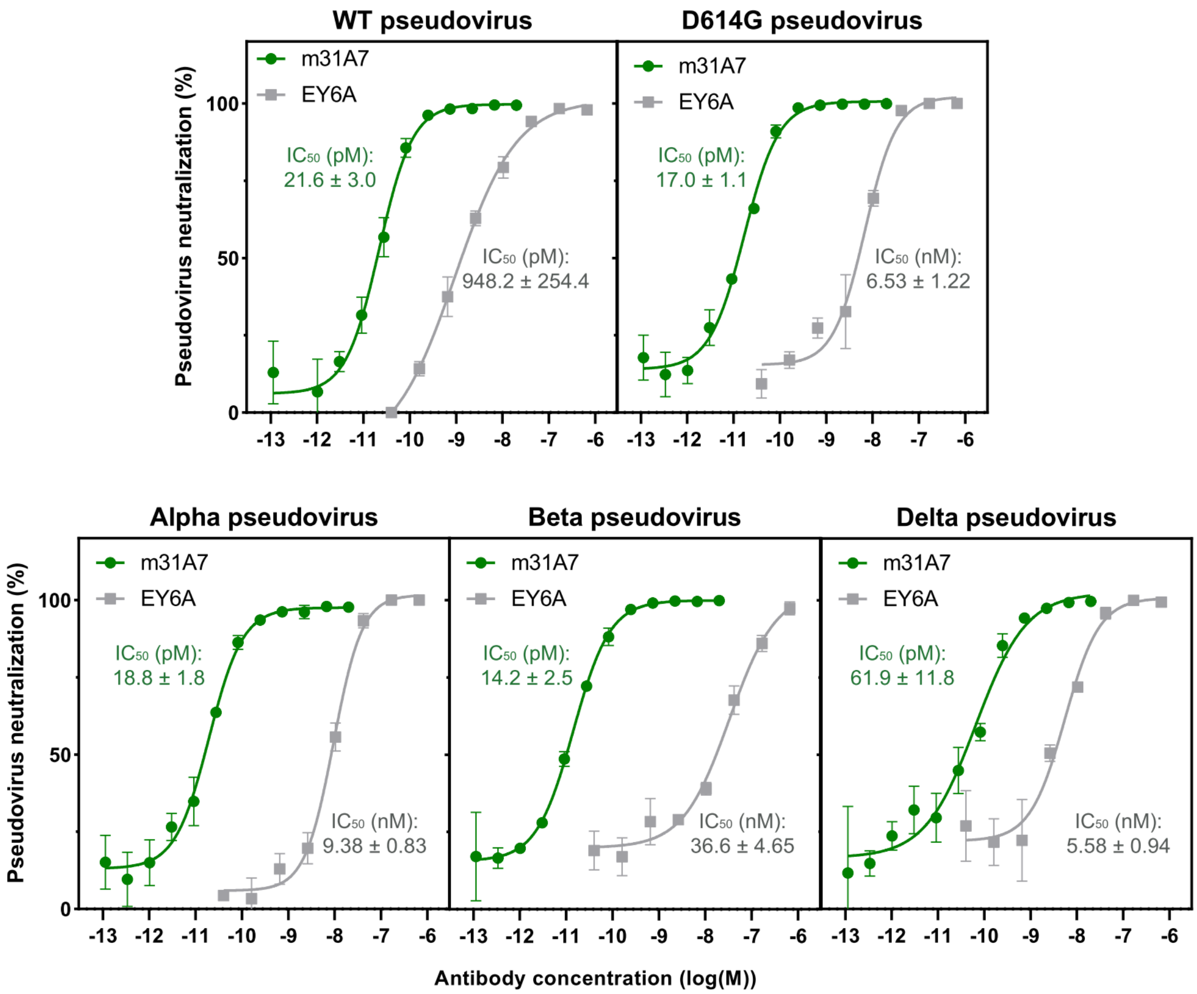

Fig. S14 Pseudovirus neutralization of SARS-CoV-2 WT and variants by m31A7. Pseudovirus neutralization of SARS-CoV-2 WT and variants by m31A7 (green) is shown in comparison with the previously reported mAb EY6A (gray). Variant species are labeled on top of each panel. The curves were fitted with nonlinear regression using Graph Prism 9.0 to obtain the sub-picomolar half maximal inhibitory concentration (IC_{50}) values which are shown beside the curves. Data of 3 replicates are presented as mean ± SD.

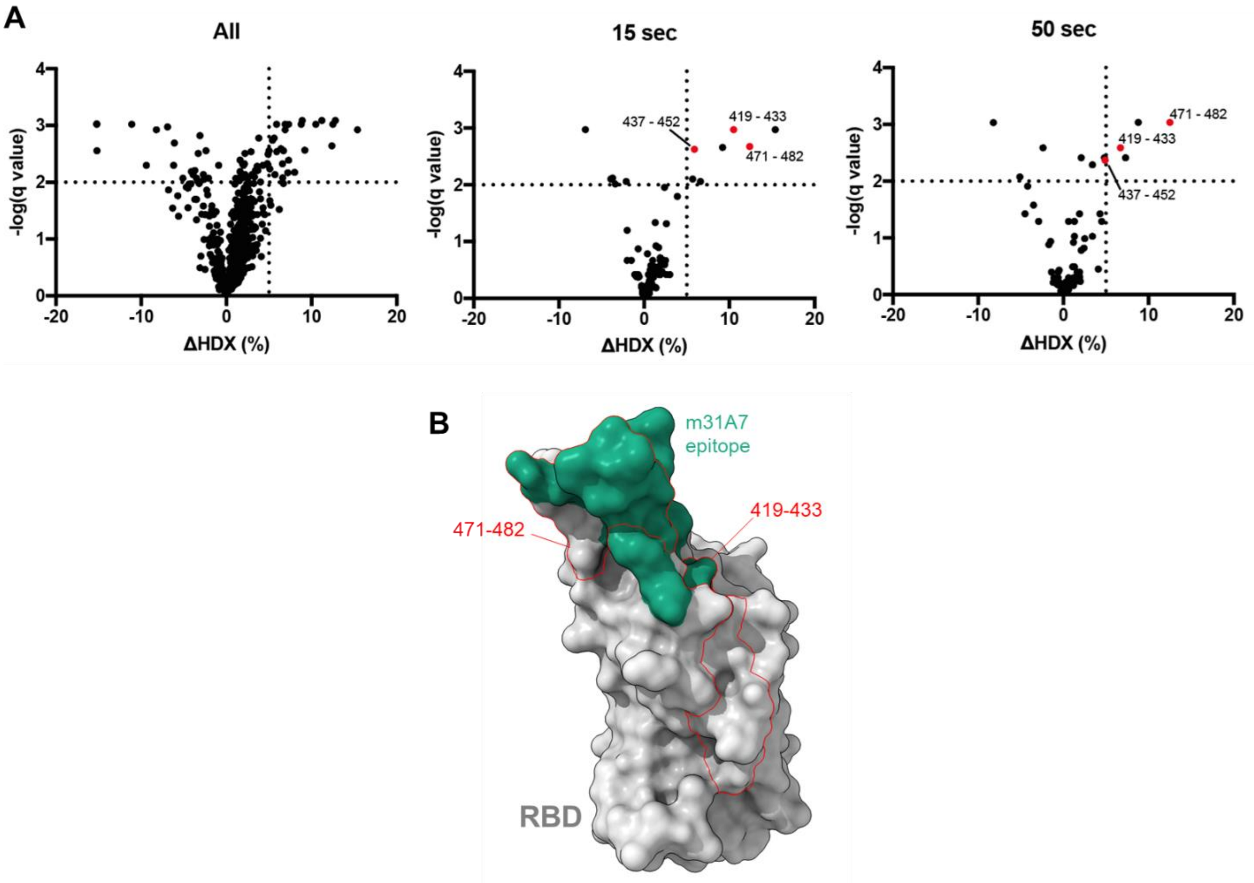

Fig. S15. Epitope mapping of RBD bound by m31A7 using hydrogen-deuterium exchange mass spectrometry (HDX-MS). (A) Volcano plots of the changes in deuterium uptake in RBD upon addition of m31A7 IgG, with the hits (ΔHDX > 5%, q value < 0.01) shown in the top right corner and highlighted in red. Detailed ΔHDX curves of the highlighted peptides are shown in Fig. 4H. Unlabeled hits are redundant peptides with 471 to 482. **(B)** Mapping of the two interacting peptides on RBD structure identified in HDX shown as red contour lines, with the observed epitope in colored in green (PDB 7WUE from this study) and the rest of RBD in gray.

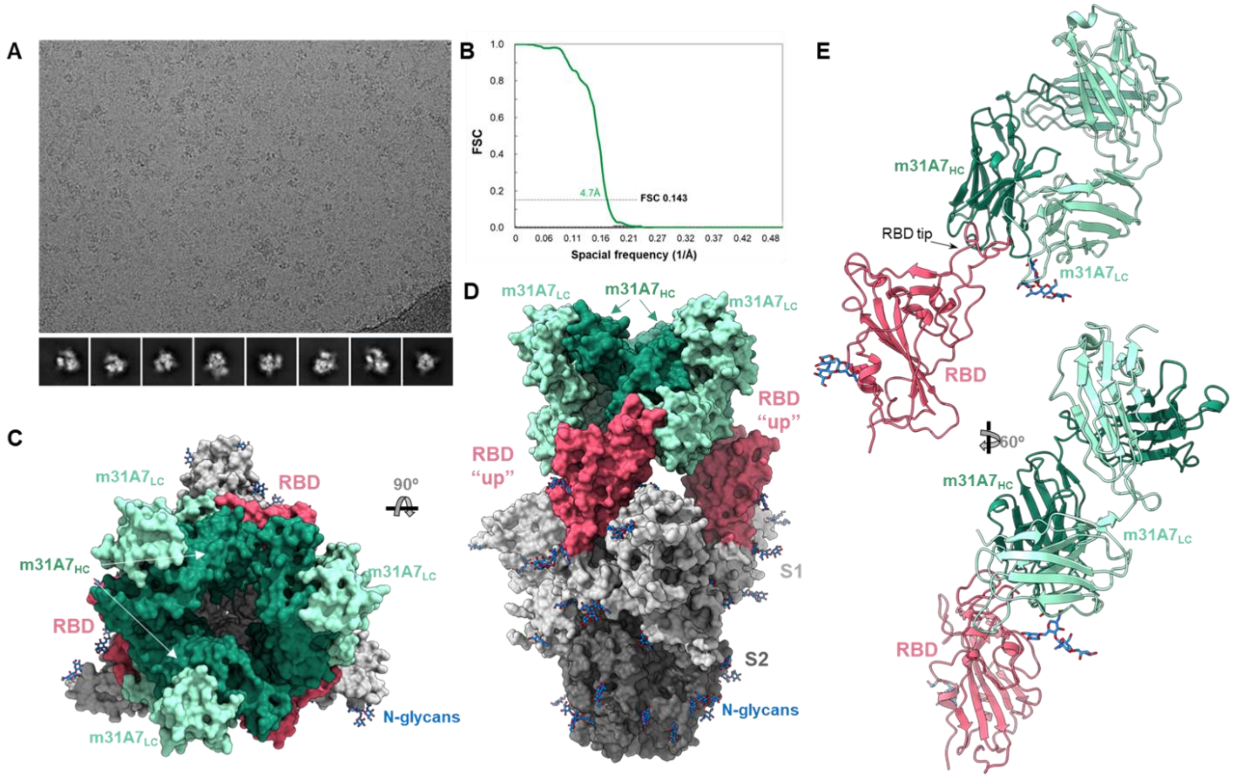

Fig. S16. Cryo-EM and crystal structures of m31A7-Fab-bound S protein. (A) Raw image and 2D class average (selected classes shown below) used for 3D reconstitution and refinements. **(B)** Fourier shell correlation (FSC) curve shown for the final cryo-EM volume. **(C)** Top view and **(D)** side view of the m31A7-Fab-bound full-length S protein shown in surface representation. **(E)** Views of the m31A7-Fab-bound RBD crystal structure. Rotating angles are indicated in figure. Light gray: S1; Dark gray: S2; Red: RBD; Light green: Fab light chain; Dark green: Fab heavy chain. Blue: N-glycans.

Table S1. Compositions and types of *N*-glycans observed across the SARS-CoV-2 S protein from human lung epithelial (BEAS-2B) cells.

N-glycan type [a]	(%) Percentage of *N*-Glycan in Individual Glycosylation Sites [b]																							Total [c]
	N17	N61	N74	N122	N149	N165	N234	N282	N331	N343	N603	N616	N657	N709	N717	N801	N1074	N1098	N1134	N1158	N1173	N1194		
**Individual *N*-Glycan**																								
FA4/A3B-S	0	0	21	0	0	2	0	17	1	0	0	3	3	0	0	0	0	0	0	0	0	29	FA4/A3B-S	3
FA3/A2B-S	0	0	63	0	0	12	0	19	23	3	0	13	90	7	0	0	18	30	0	0	0	71	FA3/A2B-S	16
FA2/A1B-S	50	0	16	33	0	34	0	5	76	37	0	5	8	0	0	28	12	0	0	0	0	0	FA2/A1B-S	14
FA1-S	0	0	0	0	0	0	0	0	0	0	0	0	0	0	0	0	0	0	0	0	0	0	FA1-S	0
A4/A3B-S	6	0	0	0	11	50	0	0	0	0	0	0	0	0	0	0	0	8	0	0	0	0	A4/A3B-S	3
A3/A2B-S	44	1	0	0	61	0	0	0	0	0	0	0	0	43	0	0	0	62	0	0	0	0	A3/A2B-S	10
A2/A1B-S	0	0	0	21	28	0	0	0	0	0	0	0	0	0	0	10	0	0	0	100	0	0	A2/A1B-S	7
A1-S	0	0	0	0	0	0	0	0	0	0	0	0	0	0	0	0	0	0	0	0	0	0	A1-S	0
FA4/A3B	0	0	0	0	0	0	0	18	0	0	2	8	0	9	0	0	5	0	0	0	0	0	FA4/A3B	2
FA3/A2B	0	0	0	0	0	0	0	25	0	0	44	48	0	16	0	0	12	0	0	0	57	0	FA3/A2B	9
FA2/A1B	0	0	0	0	0	0	0	16	1	61	54	24	0	24	0	0	19	0	0	0	44	0	FA2/A1B	11
FA1	0	0	0	0	0	0	0	0	0	0	0	0	0	0	0	0	1	0	0	0	0	0	FA1	0
A4/A3B	0	0	0	0	0	0	0	0	0	0	0	0	0	0	0	0	0	0	0	0	0	0	A4/A3B	0
A3/A2B	0	1	0	0	0	0	0	0	0	0	0	0	0	0	0	0	0	0	62	0	0	0	A3/A2B	3
A2/A1B	0	4	0	0	0	0	0	0	0	0	0	0	0	0	0	0	1	0	0	0	0	0	A2/A1B	0
A1	0	0	0	0	0	0	0	0	0	0	0	0	0	0	0	0	2	0	0	0	0	0	A1	0
Fybrid-S	0	0	0	0	0	0	0	0	0	0	0	0	0	2	0	0	3	0	0	0	0	0	Fybrid-S	0
Hybrid-S	0	0	0	0	0	0	0	0	0	0	0	0	0	0	0	1	2	0	0	0	0	0	Hybrid-S	0
Fybrid	0	0	0	0	0	0	0	0	0	0	0	0	0	0	0	0	0	0	0	0	0	0	Fybrid	0
Hybrid	0	0	0	0	0	0	0	0	0	0	0	0	0	0	3	0	4	0	0	0	0	0	Hybrid	0
M4	0	0	0	0	0	0	0	0	0	0	0	0	0	0	0	0	0	0	0	0	0	0	M4	0
M5	0	22	0	0	0	0	0	0	0	0	0	0	0	0	1	0	1	0	0	0	0	0	M5	1
M6	0	64	0	7	0	4	6	0	0	0	0	0	0	0	29	6	17	0	0	0	0	0	M6	6
M7	0	9	0	39	0	0	18	0	0	0	0	0	0	0	67	52	4	0	0	0	0	0	M7	9
M8	0	1	0	0	0	0	74	0	0	0	0	0	0	0	1	2	1	0	0	0	0	0	M8	4
M9	0	0	0	0	0	0	3	0	0	0	0	0	0	0	0	0	0	0	0	0	0	0	M9	0
unoccupied	0	0	1	0	0	0	0	0	0	0	0	0	0	0	0	0	0	0	38	0	0	0	unoccupied	2
**Categorized**																								
Complex-S	100	1	99	54	100	97	0	41	99	39	0	21	100	50	0	38	30	100	0	100	0	100	Complex-S	53
Complex	0	5	0	0	0	0	0	59	1	61	100	79	0	48	0	0	40	0	62	0	100	0	Complex	25
Hybrid-S	0	0	0	0	0	0	0	0	0	0	0	0	0	2	0	1	4	0	0	0	0	0	Hybrid-S	0
Hybrid	0	0	0	0	0	0	0	0	0	0	0	0	0	0	3	0	4	0	0	0	0	0	Hybrid	0
Oligomannose	0	95	0	46	0	4	100	0	0	0	0	0	0	0	98	60	22	0	0	0	0	0	Oligomannose	19
Sialylation	100	1	99	54	100	97	0	41	99	39	0	21	100	52	0	39	34	100	0	100	0	100	Sialylation	53
Fucosylation	50	0	99	33	0	47	0	100	100	100	100	100	100	57	0	28	70	30	0	0	100	100	Fucosylation	55
Unoccupied	0	0	1	0	0	0	0	0	0	0	0	0	0	0	0	0	0	0	38	0	0	0	Unoccupied	2

[a] The N-glycans was defined and categorized as described *(2).* [b] The *N*-glycan types and percentages on each glycosite were determined using the liquid chromatography coupled with tandem mass spectrometry LC-MS/MS. [c] The percentages of *N*-glycans on S protein, without considering individual glycosites, was calculated by dividing the amount of *N*-glycans on all site by the number of glycosylation sites (22 for S proteins) *(2).*

Table S2. Compositions and types of *N*-glycans on SARS-CoV-2 S protein from human epithelial kidney 293T cells.

N-glycan type [a]	(%) Percentage of *N*-Glycan in Individual Glycosylation Sites [b]																							Total [c]
	N17	N61	N74	N122	N149	N165	N234	N282	N331	N343	N603	N616	N657	N709	N717	N801	N1074	N1098	N1134	N1158	N1173	N1194		
**Individual *N*-Glycan**																								
FA4/A3B-S	0	0	0	0	0	1	0	0	1	1	0	1	9	0	0	0	1	1	5	0	87	100	FA4/A3B-S	9
FA3/A2B-S	0	0	6	2	0	6	0	3	12	1	0	3	17	0	0	0	2	2	12	0	6	0	FA3/A2B-S	3
FA2/A1B-S	0	0	15	9	0	40	0	2	34	12	1	7	41	0	0	3	7	2	7	0	0	0	FA2/A1B-S	8
FA1-S	0	0	0	0	0	0	0	0	0	0	0	0	0	0	0	0	0	0	0	0	0	0	FA1-S	0
A4/A3B-S	4	0	0	0	0	0	0	0	0	0	0	0	0	0	0	0	0	2	1	100	0	0	A4/A3B-S	5
A3/A2B-S	6	0	0	0	0	0	0	0	0	0	0	0	0	0	0	0	0	8	0	0	0	0	A3/A2B-S	1
A2/A1B-S	27	1	0	5	0	4	0	0	0	0	0	4	0	0	0	2	1	2	0	0	0	0	A2/A1B-S	2
A1-S	0	0	0	0	0	0	0	0	0	0	0	1	0	0	0	0	0	0	0	0	0	0	A1-S	0
FA4/A3B	21	0	6	0	0	1	0	5	0	0	1	2	0	1	0	0	1	0	11	0	7	0	FA4/A3B	3
FA3/A2B	0	0	69	3	0	15	0	43	25	28	11	12	7	3	1	3	10	4	23	0	0	0	FA3/A2B	12
FA2/A1B	14	2	0	4	0	15	0	39	18	36	40	31	7	30	0	4	22	13	22	0	0	0	FA2/A1B	13
FA1	0	0	0	0	0	0	0	0	0	0	1	0	0	0	0	0	0	1	1	0	0	0	FA1	0
A4/A3B	0	1	4	0	0	0	0	1	1	0	0	0	0	0	0	0	1	0	1	0	0	0	A4/A3B	0
A3/A2B	0	7	0	2	0	2	0	4	3	10	3	2	0	0	0	1	2	0	1	0	0	0	A3/A2B	2
A2/A1B	0	11	0	5	0	2	0	1	0	0	1	5	0	8	1	2	5	1	1	0	0	0	A2/A1B	2
A1	0	1	0	0	0	0	0	0	0	0	0	0	0	0	0	0	0	0	0	0	0	0	A1	0
Fybrid-S	22	0	0	8	0	0	0	0	2	1	0	1	0	4	0	1	12	8	3	0	0	0	Fybrid-S	3
Hybrid-S	2	1	0	18	0	1	0	0	0	0	0	0	0	4	5	21	1	24	0	0	0	0	Hybrid-S	4
Fybrid	0	2	0	5	0	0	0	0	3	9	17	16	1	12	2	6	26	7	11	0	0	0	Fybrid	5
Hybrid	0	32	0	25	100	3	4	1	0	0	8	13	2	0	26	18	4	18	2	0	0	0	Hybrid	12
M4	0	0	0	1	0	0	0	0	0	0	0	0	0	0	1	1	0	1	0	0	0	0	M4	0
M5	0	40	0	11	0	10	2	0	0	1	13	1	14	19	19	29	5	5	0	0	0	0	M5	8
M6	2	2	0	0	0	0	3	0	0	0	1	0	1	13	20	5	1	0	0	0	0	0	M6	2
M7	0	0	0	1	0	0	14	0	0	0	1	0	0	5	25	4	0	1	0	0	0	0	M7	2
M8	0	0	0	0	0	0	51	0	0	0	0	0	0	0	0	0	0	0	0	0	0	0	M8	2
M9	0	0	0	0	0	0	24	0	0	0	0	0	0	0	0	0	0	0	0	0	0	0	M9	1
unoccupied	0	0	0	0	0	0	0	0	0	0	0	0	0	0	0	0	0	0	0	0	0	0	unoccupied	0
**Categorized**																								
Complex-S	38	1	21	16	0	50	0	5	47	14	1	16	68	0	0	5	11	19	25	100	93	100	Complex-S	29
Complex	35	21	79	14	0	36	0	93	48	74	58	53	14	43	1	10	41	19	59	0	7	0	Complex	32
Hybrid-S	24	1	0	26	0	1	0	0	2	1	0	1	0	8	5	22	12	32	3	0	0	0	Hybrid-S	6
Hybrid	0	33	0	30	100	3	4	1	3	10	25	29	3	12	28	24	30	25	13	0	0	0	Hybrid	17
Oligomannose	2	43	0	14	0	10	96	0	0	1	16	1	16	38	65	39	6	6	0	0	0	0	Oligomannose	16
Sialylation	63	2	21	42	0	52	0	5	49	15	1	17	68	7	6	27	23	50	28	100	93	100	Sialylation	35
Fucosylation	59	3	96	44	0	80	0	92	92	79	54	57	82	42	6	32	55	56	83	0	100	100	Fucosylation	55
Unoccupied	0	0	0	0	0	0	0	0	0	0	0	0	0	0	0	0	0	0	0	0	0	0	Unoccupied	0

[a] The N-glycans was defined and categorized as described *(2)*. [b] The *N*-glycan types and percentages on each glycosite were determined by using LC-MS/MS. [c] The percentages of *N*-glycans on S protein, without considering individual glycosites, was calculated by dividing the amount of *N*-glycans on all site by the number of glycosylation sites (22 for S proteins) *(2)*.

Table S3. Compositions and types of N-glycans on SARS-CoV-2 S protein rom human epithelial kidney 293E cells.

N-glycan type [a]	(%) Percentage of *N*-Glycan in Individual Glycosylation Sites [b]																							Total [c]
	N17	N61	N74	N122	N149	N165	N234	N282	N331	N343	N603	N616	N657	N709	N717	N801	N1074	N1098	N1134	N1158	N1173	N1194		
**Individual *N*-Glycan**																								
FA4/A3B-S	0	0	0	0	0	0	0	0	0	0	0	0	0	0	0	0	3	11	5	0	85	0	FA4/A3B-S	5
FA3/A2B-S	0	0	11	3	69	4	0	7	14	2	0	8	0	0	0	0	11	9	0	0	4	0	FA3/A2B-S	6
FA2/A1B-S	28	0	0	18	0	11	0	0	29	6	0	9	0	0	0	11	7	9	11	0	0	0	FA2/A1B-S	6
FA1-S	0	0	0	0	0	0	0	0	0	0	0	0	0	0	0	0	0	0	0	0	0	0	FA1-S	0
A4/A3B-S	0	0	0	0	0	0	0	0	0	0	0	0	0	0	0	0	0	28	0	0	0	0	A4/A3B-S	1
A3/A2B-S	0	0	0	0	0	0	0	0	0	0	0	0	65	0	0	0	0	14	0	0	0	0	A3/A2B-S	4
A2/A1B-S	0	1	0	6	0	0	0	0	0	0	0	0	35	0	0	7	0	19	0	0	0	0	A2/A1B-S	3
A1-S	0	0	0	0	0	0	0	0	0	0	0	0	0	0	0	0	0	0	0	0	0	0	A1-S	0
FA4/A3B	0	0	25	0	0	3	0	22	2	0	0	4	0	0	0	0	9	0	29	0	11	0	FA4/A3B	5
FA3/A2B	72	0	61	20	31	58	0	64	45	50	39	30	0	0	0	14	28	0	28	0	0	38	FA3/A2B	26
FA2/A1B	0	3	0	10	0	9	0	7	8	24	49	42	0	10	3	4	16	0	28	100	0	62	FA2/A1B	17
FA1	0	0	0	0	0	0	0	0	0	1	0	0	0	0	3	0	1	0	0	0	0	0	FA1	0
A4/A3B	0	0	0	0	0	0	0	0	0	0	0	0	0	0	0	0	1	0	0	0	0	0	A4/A3B	0
A3/A2B	0	7	0	10	0	0	0	0	0	0	0	1	0	0	0	5	0	0	0	0	0	0	A3/A2B	1
A2/A1B	0	6	0	7	0	0	0	0	0	0	0	3	0	0	0	3	1	5	0	0	0	0	A2/A1B	1
A1	0	0	0	0	0	0	0	0	0	0	0	0	0	0	1	0	0	0	0	0	0	0	A1	0
Fybrid-S	0	0	0	1	0	0	0	0	0	0	0	0	0	0	1	0	1	0	0	0	0	0	Fybrid-S	0
Hybrid-S	0	0	0	3	0	0	0	0	0	0	0	0	0	0	7	14	0	0	0	0	0	0	Hybrid-S	1
Fybrid	0	0	0	2	0	0	0	0	0	1	0	0	0	0	4	0	2	0	0	0	0	0	Fybrid	0
Hybrid	0	5	0	6	0	0	0	0	0	1	0	0	0	0	19	4	3	2	0	0	0	0	Hybrid	2
M4	0	1	0	0	0	0	0	0	0	0	0	0	0	0	0	1	0	0	0	0	0	0	M4	0
M5	0	76	3	11	0	16	4	0	2	14	12	3	0	58	19	30	15	3	0	0	0	0	M5	12
M6	0	2	0	0	0	0	6	0	0	0	0	0	0	19	16	3	2	0	0	0	0	0	M6	2
M7	0	0	0	2	0	0	27	0	0	0	0	0	0	14	27	5	0	0	0	0	0	0	M7	3
M8	0	0	0	0	0	0	54	0	0	0	0	0	0	0	0	0	0	0	0	0	0	0	M8	2
M9	0	0	0	0	0	0	8	0	0	0	0	0	0	0	0	0	0	0	0	0	0	0	M9	0
unoccupied	0	0	0	0	0	0	0	0	0	0	0	0	0	0	0	0	0	0	0	0	0	0	unoccupied	0
**Categorized**																								
Complex-S	28	1	11	28	69	19	0	7	43	9	0	17	100	0	0	18	22	90	11	0	70	0	Complex-S	25
Complex	72	15	86	49	31	71	0	93	55	74	88	80	0	10	7	25	62	5	89	100	15	100	Complex	51
Hybrid-S	0	0	0	3	0	0	0	0	0	0	0	0	0	0	8	14	1	0	0	0	0	0	Hybrid-S	1
Hybrid	0	5	0	8	0	0	0	0	0	2	0	0	0	0	23	4	4	2	0	0	15	0	Hybrid	3
Oligomannose	0	78	3	12	0	10	100	0	2	15	12	3	0	90	63	39	11	3	0	0	0	0	Oligomannose	20
Sialylation	28	1	11	32	69	19	0	7	43	9	0	17	100	0	8	32	23	90	11	0	70	0	Sialylation	26
Fucosylation	100	3	97	55	0	84	0	100	98	84	88	92	0	10	11	29	78	29	100	100	100	100	Fucosylation	62
Unoccupied	0	0	0	0	0	0	0	0	0	0	0	0	0	0	0	0	0	0	0	0	0	0	Unoccupied	0

[a] The N-glycans was defined and categorized as described *(2)*. [b] The *N*-glycan types and percentages on each glycosite were determined by using LC-MS/MS. [c] The percentages of *N*-glycans on S protein, without considering individual glycosites, was calculated by dividing the amount of *N*-glycans on all site by the number of glycosylation sites (22 for S proteins) *(2)*.
